# Earliest Example of a Giant Monitor Lizard (*Varanus*, Varanidae, Squamata)

**DOI:** 10.1371/journal.pone.0041767

**Published:** 2012-08-10

**Authors:** Jack L. Conrad, Ana M. Balcarcel, Carl M. Mehling

**Affiliations:** 1 Anatomy Department, New York College of Osteopathic Medicine, Old Westbury, New York, United States of America; 2 Department of Vertebrate Paleontology, American Museum of Natural History, New York, New York, United States of America; Monash University, Australia

## Abstract

**Background:**

Varanidae is a clade of tiny (<20 mm pre-caudal length [PCL]) to giant (>600 mm PCL) lizards first appearing in the Cretaceous. True monitor lizards (*Varanus*) are known from diagnostic remains beginning in the early Miocene (*Varanus rusingensis*), although extremely fragmentary remains have been suggested as indicating earlier *Varanus*. The paleobiogeographic history of *Varanus* and timing for origin of its gigantism remain uncertain.

**Methodology/Principal Findings:**

A new *Varanus* from the Mytilini Formation (Turolian, Miocene) of Samos, Greece is described. The holotype consists of a partial skull roof, right side of a braincase, partial posterior mandible, fragment of clavicle, and parts of six vertebrae. A cladistic analysis including 83 taxa coded for 5733 molecular and 489 morphological characters (71 previously unincluded) demonstrates that the new fossil is a nested member of an otherwise exclusively East Asian *Varanus* clade. The new species is the earliest-known giant (>600 mm PCL) terrestrial lizard. Importantly, this species co-existed with a diverse continental mammalian fauna.

**Conclusions/Significance:**

The new monitor is larger (longer) than 99% of known fossil and living lizards. *Varanus* includes, by far, the largest limbed squamates today. The only extant non-snake squamates that approach monitors in maximum size are the glass-snake *Pseudopus* and the worm-lizard *Amphisbaena*. Mosasauroids were larger, but exclusively marine, and occurred only during the Late Cretaceous. Large, extant, non-*Varanus*, lizards are limbless and/or largely isolated from mammalian competitors. By contrast, our new *Varanus* achieved gigantism in a continental environment populated by diverse eutherian mammal competitors.

## Introduction

Monitor lizards (Varanidae) are known to have originated by the Late Cretaceous [1]–[4]. Although varanids are best popularly known by the Komodo Dragon (*Varanus komodoensis*), and are famous for reaching large body sizes, the earliest monitors were small- to medium-sized lizards (e.g., the Late Cretaceous forms *Ovoo gurvel*, *Aiolosaurus oriens*, and *Telmasaurus grangeri*) [4]–[6]. Large varanids are noticeably absent from Cretaceous deposits, despite (or, possibly, because of) the apparent ability of monstersaurs to grow to large sizes during those times (e.g., *Estesia mongoliensis* and *Palaeosaniwa canadensis*) [1], [7]–[9]. Indeed, large varanids do not appear in the fossil record until the Eocene taxon *Saniwa ensidens* [10]–[12], and the earliest example of a described giant monitor lizard is from the Pleistocene (*Varanus priscus*) [13]–[16].

More than 70 extant true monitor lizard (*Varanus*) species are known from across Africa, Eurasia, and Australia [17]–[20], ranging in size from tiny (approximately 100 mm in pre-caudal length [PCL]) to giant (multiple species exceeding 600 mm in PCL) [21], [22] (Tables 1, 2), encompassing more than three orders of magnitude in mass [23], and exploiting a broad diversity of ecological specializations (e.g., arboreal, semi-aquatic, xeric terrestrial) [17], [22], [23]. However, the *Varanus* fossil record is sparse and most fossil species are represented only by isolated vertebrae [16], [24]. The poor fossil record leaves open many questions about the evolution of the group, including: When and where did monitors originate and diversify? When did they first achieve giant sizes? Can they achieve gigantism in the presence of mammalian competitors [25]–[27]?

**Table 1:** Principal data from the measurement of 28 specimens of *Varanus* for comparison of fossils forms and size predictions.

specimen #	*Varanus* sp.	BCL	DVL	PCL
AMNH R-47725	*Albigularis*	37	17	630
AMNH R 141072	*Beccarii*	18.5	8	313
AMNH R 29932	*Bengalensis*	25.28	14	510
AMNH R 118713	*Bengalensis*	30.23	17	660
AMNH R 140804	*exanthematicus*	28	16	470
AMNH R 77646	*Flavescens*	21.72	10.05	339
UF 64743	*Flavescens*	19	9.5	350
AMNH R 82819	*Gouldii*	28	14.5	650
AMNH R 74810	*Griseus*	20.5	9.81	423
AMNH R 142617	*Indicus*	12	5.5	212
AMNH R 142623	*Indicus*	14.58	6.93	244.44
AMNH R 37908	*Komodoensis*	44	19	718
AMNH R 37909	*Komodoensis*	87	36	1478
AMNH R 7252	*Niloticus*	23	9	360
AMNH R 137116	*niloticus*	45	25	832
AMNH R 10499	*Ornatus*	29.5	9	393
AMNH R 104683	*Prasinus*	16	8	283
AMNH R 141071	*Rudicollis*	13.5	6	196
AMNH R 49230	*salvator?*	36.5	17	660
UF 99317	*Timorensis*	10.5	4.5	174
UF 45363	*Timorensis*	10	4.5	180

Abbreviations: BCL, linear length of the braincase from the anterior tip of the basipterygoid process of the parabasisphenoid to the tip of the paroccipital process of the otooccipital; DVL, length of a posterior dorsal vertebra; PCL, pre-caudal length of the animal (an osteological skeletal length similar to the snout-vent length often used for extant taxa. All measurements in millimeters [mm]).

**Table 2:** SIZES OF EXTANT VARANIDAE.

Genus	species/subspecies	SVL (mm)	TL (mm)	sex	comment
*Lanthanotus*	*borneensis*	400			max
*Varanus*	*eremius*	59	150		approx
*Varanus*	*brevicauda*	70	136		approx
*Varanus*	*kingorum*	98.4	326		mean
*Varanus*	*gilleni*	113	235		
*Varanus*	*brevicauda*	118	230		
*Varanus*	*caudolineatus*	118		F	
*Varanus*	*primordius*	120			approx
*Varanus*	*caudolineatus*	125		M	
*Varanus*	*storri*	132	300		approx
*Varanus*	*eremius*	160	400		approx
*Varanus*	*pilbarensis*	169			max
*Varanus*	*baritji*	171	468		mean
*Varanus*	*glauerti*	180	324	F	approx
*Varanus*	*gilleni*	186			
*Varanus*	*glauerti*	215	387	M	approx
*Varanus*	*glebopalma*	245	409.2	F	approx
*Varanus*	*scalaris*	250	600		max
*Varanus*	*semiremex*	250	600		
*Varanus*	*griseus koniecznyi*	255	620		
*Varanus*	*keithhornei*	260	650		
*Varanus*	*kordensis*	270	580		max field
*Varanus*	*glebopalma*	290	484.3	M	approx
*Varanus*	*dumerilii*	292	900		
*Varanus*	*prasinus*	295	845		max
*Varanus*	*tristis*	305	800		large
*Varanus*	*macrei*	313	912	F	
*Varanus*	*flavescens*	315	699		
*Varanus*	*exanthematicus*	320	640	M	mean
*Varanus*	*exanthematicus*	320	640	F	mean
*Varanus*	*mitchelli*	320			max
*Varanus*	*rudicollis*	336			mean
*Varanus*	*macrei*	340	1000	M	
*Varanus*	*griseus griseus*	341	830		
*Varanus*	*marmoratus*	342		F	
*Varanus*	*salvadorii*	350	1160		
*Varanus*	*macrei*	360	1110	M	
*Varanus*	*salvadorii*	360	1110		
*Varanus*	*gouldii*	361	931	F	
*Varanus*	*caerulivirensis*	375	985	F	max
*Varanus*	*marmoratus*	391		M	
*Varanus*	*salvator*	397	982		
*Varanus*	*caerulivirensis*	400	1040	M	max
*Varanus*	*melinus*	420	1150	M	
*Varanus*	*cumingi*	431	1142		mean
*Varanus*	*griseus caspius*	432	1050		
*Varanus*	*jobiensis*	445	1195	M	
*Varanus*	*jobiensis*	450	1185	F	
*Varanus*	*salvator*	453	1198		
*Varanus*	*yemenensis*	458	999		
*Varanus*	*bengalensis*	460	1200	F	
*Varanus*	*doreanus*	460	1255		voucher
*Varanus*	*rosenbergi*	470			max
*Varanus*	*salvadorii*	478	1486		
*Varanus*	*mertensi*	480	1300		max
*Varanus*	*albigularis*	500	1111	F	
*Varanus*	*albigularis*	500	1111	M	
*Varanus*	*olivaceus*	509		F	
*Varanus*	*niloticus*	523	1308	F	
*Varanus*	*mabitang*	527	1268	F	type
*Varanus*	*yuwonoi*	532	1877	F	holotype
*Varanus*	*spenceri*	550	1250		large
*Varanus*	*bengalensis*	580	1500	M	
*Varanus*	*indicus*	580			max
*Varanus*	*gouldii*	590	1410	M	
*Varanus*	*rudicollis*	590	1460		max
*Varanus*	*mabitang*	640	1750		
*Varanus*	*niloticus*	644	1610	M	
*Varanus*	*giganteus*	645	1494		mean
*Varanus*	*olivaceus*	650		M	
*Varanus*	*giganteus*	736	1690	M	
*Varanus*	*panoptes*	740			max
*Varanus*	*salvadorii*	745	2240		
*Varanus*	*ornatus*	760	1900		max
*Varanus*	*varius*	765	1920		large
*Varanus*	*komodoensis*	775	1550		Auffenberg approx
*Varanus*	*komodoensis*	840	1680		Auffenberg approx
*Varanus*	*komodoensis*	850	1700		mean
*Varanus*	*salvadorii*	850	2550		
*Varanus*	*salvadorii*	863	2650		
*Varanus*	*giganteus*	880	1940		approx
*Varanus*	*komodoensis*	1540	3020		max field
*Varanus*	*beccarii*		950		
*Varanus*	*melinus*		950	F	

Lengths of 52 species of Varanidae (51 species of *Varanus* and *Lanthanotus borneensis*) based on published data [21], [79]. These data were used to reconstruct the lineage sizes in Figure 5. Note that not all measurements and/or data are available for all included species. Abbreviations: SVL, snout-to-vent length of the animal; TL, total length of the animal (snout to tail tip); approx, approximate length based on published data [21]; Auffenberg approx, approximate dimensions based on data presented for wild-caught specimens in Auffenberg's study on Komodo Dragons [79]; F, female; holotype/type/voucher, measurements based on the type specimen—these data are usually reported in the case of species wherein there are few available specimens; M, male; max, reported maximum measurement; max field, reported maximum measurement of wild-caught specimens—these data are usually included when the species in question is popular in the pet trade.

**Figure 1:**
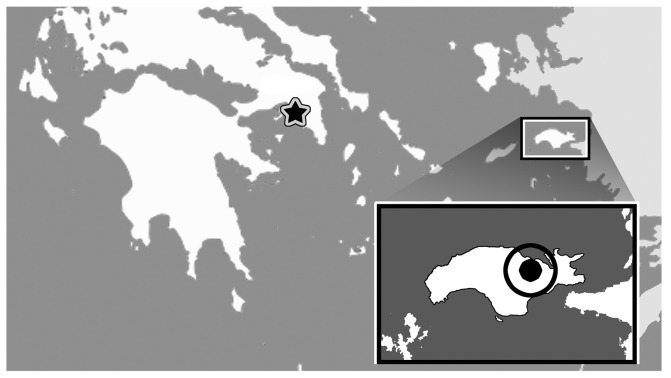
Holotype locality (circle) for *Varanus* (*Varaneades*) *amnhophilis* nov. taxon, on Samos, Greece. Star indicates Athens, Greece.

Here, we describe a new, large-bodied, *Varanus* from the Turolian Miocene of Samos, Greece (Fig. 1). The specimen was collected by Barnum Brown from his locality Q1, in association with fossil mammal remains. It remained undetected in the fossil mammal collections at the American Museum of Natural History until it was brought to the attention of one of us (CMM) by Nikos Solounias who recognized its reptilian affinities in 2009.

In addition to being diagnostic as a new species, the new taxon is a nested member of an otherwise exclusively Asian *Varanus* radiation. This new species was a giant despite occurring in a time and place (Miocene of Greece) known for its mammalian diversity and its combination of European, Asian, and African faunal influences [28]–[30].

### Institutional Abbreviations

AMNH, American Museum of Natural History (New York, NY.); BMNH PR, Natural History Museum, London (London, Great Britain); GM, Geiseltal Museum of the Martin-Luther Universität in Halle/Saale (Germany); UF, University of Florida, Florida State Museum (Gainesville, FL; ZPAL, Zoological Institute of Paleobiology, Polish Academy of Sciences, Warsaw (Poland).

### Nomenclatural Acts

The electronic version of this document does not represent a published work according to the International Code of Zoological Nomenclature (ICZN), and hence the nomenclatural acts contained in the electronic version are not available under that Code from the electronic edition. Therefore, a separate edition of this document was produced by a method that assures numerous identical and durable copies, and those copies were simultaneously obtainable (from the publication date noted on the first page of this article) for the purpose of providing a public and permanent scientific record, in accordance with Article 8.1 of the Code. The separate print-only edition is available on request from PLoS by sending a request to *PLoS One*, 185 Berry Street, Suite 3100, San Francisco, CA 94107, USA along with a check for $10 (to cover printing and postage) payable to “Public Library of Science.”

In addition, this published work and the nomenclatural acts it contains have been registered in ZooBank, the proposed online registration system for the ICZN. The ZooBank LSIDs (Life Science Identifiers) can be resolved and the associated information viewed through any standard web browser by appending the LSID to the prefix http://zoobank.org/. The LSID for this publication is urn:lsid:zoobank.org:pub:2E46BE4B-A001-4879-BD9D-4F203743D1DB.

Clade names follow recent phylogenetic definitions [2], [3].

### Systematic Paleontology

Squamata Oppel 1811

Anguimorpha Fürbringer 1900

Varanidae Gray 1827

*Varanus* White 1790

*Varanus* (*Varaneades*) *amnhophilis*, gen. et sp. nov.

(*Varaneades*) subgen. nov.

ZooBank LSID urn:lsid:zoobank.org:act:FCD0FAC3-D8C5-4308-912D-7B0DBA0C8837

*Varanus* (*Varaneades*) *amnhophilis* sp. nov.

ZooBank LSID urn:lsid:zoobank.org:act:65B2D84B-B7FB-48A0-80F2-5E268C6E1E5D

Figures 2–3.

**Figure 2:**
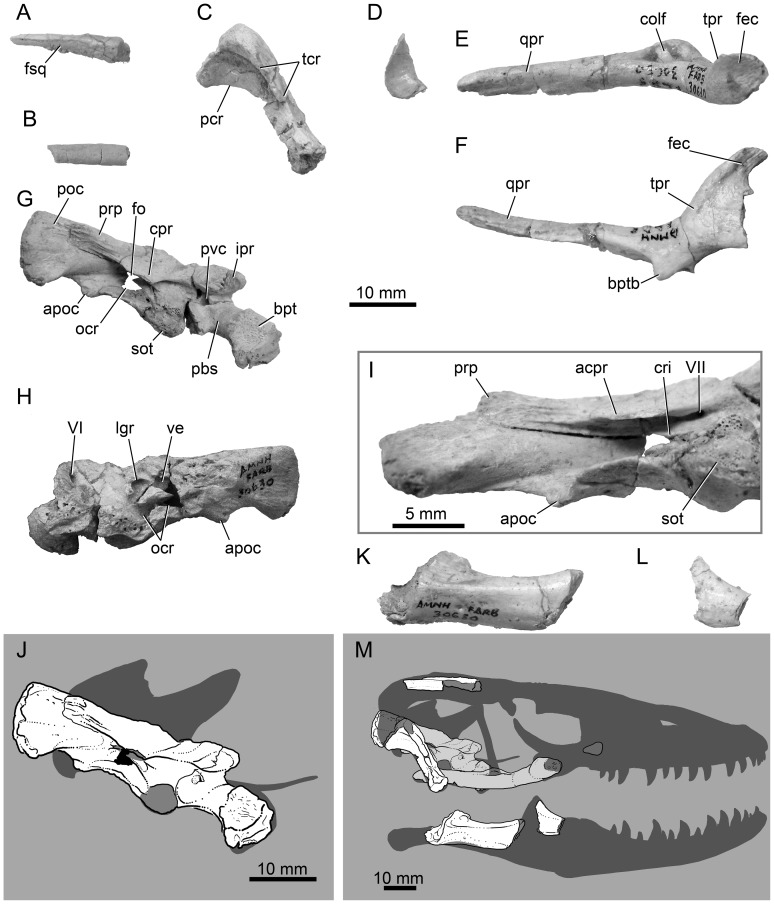
Holotype (AMNH FR 30630) skull elements for *Varanus* (*Varaneades*) *amnhophilis* nov. taxon. Fragmentary right postorbital (A) and squamosal (B) in lateral view. (C) Right quadrate in lateral view. (D) Fragmentary palatine in ventral view. Right pterygoid in lateral (E) and ventral (F) view. Note the absence of pterygoid teeth. Right side of the braincase (parabasisphenoid, prootic, basioccipital, and otooccipital) in lateral view (G) and medial view (H). (I) Otic region of the braincase in ventral view showing the base of the crista interfenestralis and single opening to the facial foramen. (J) Reconstruction of the braincase in right lateral view with reconstructed areas appearing as semi-opaque shadows. (K) Partial right surangular-prearticular/articular complex in lateral view. (L) Partial right coronoid in lateral view. (M) Reconstruction of the cranium and mandible in right lateral view with reconstructed areas appearing as semi-opaque shadows. All scale bars 10 mm, except in (I) wherein the scale bar is 5 mm. Abbreviations: apoc, paroccipital tuberosity; acpr, anterostapedial process of the prootic crest; bpt, basipterygoid process; bptb, basipterygoid buttress; colf, columellar fossa; cpr, prootic crest (crista prootica); cri, crista interfenestralis; fec, ectopterygoid facet; fo, fenestra ovalis; fsq, squamosal facet (on postorbital); ipr, inferior process; pbs, parabasisphenoid; pcr, posterior crest; ped, hypapophyseal pedicel; poc, otooccipital paroccipital process; prp, prootic paroccipital process; poz, postzygapophysis; pvc, posterior opening of the vidian canal; qpr, quadrate process; sot, spheno-occipital tubercle; syn, synapophysis; tcr, tympanic crest; tpr, transverse process; I–XII, cranial nerves.

**Figure 3:**
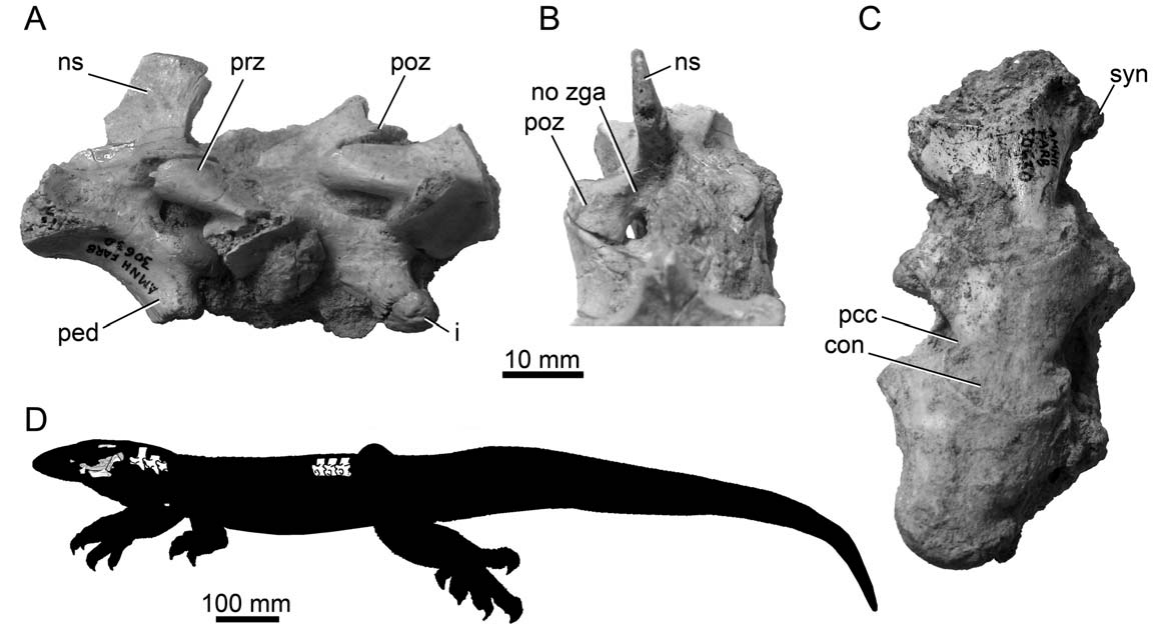
Holotype (AMNH FR 30630) vertebrae for *Varanus* (*Varaneades*) *amnhophilis* nov. taxon. (A) Cervical vertebrae 3, 4, and part of 5 in left lateral view. (B) Cervical vertebrae 3 and 4 in posterodorsal view showing the absence of zygosphenes/zygantra and/or pseudozygosphenes/pseudozygantra. (C) Three posterior dorsal vertebrae in ventral view. (D) Reconstruction of AMNH FR 30630 in left lateral view with known parts illustrated on a hypothetical black silhouette for the outline of the animal as a whole. Abbreviations: con, condyle; i, intercentrum; ns, neural spine; pcc, area of precondylar constriction; ped, hypapophyseal pedicel; poz, postzygapophysis; prz, prezygapophysis; syn, synapophysis; zga, zygantrum/pseudozygantrum.

#### Holotype

AMNH FR 30630; right side of braincase (prootic, parabasisphenoid, otooccipital, basioccipital), right quadrate, partial right coronoid, partial palate, skull roof fragments, glenoid region of right mandible, a small fragment of the right clavicle, and five-and-a-half presacral vertebrae (Figs. 2, 3).

#### Etymology

*Varaneades* (subgenus) from “*Varanus*” modern true monitor lizards, and “Neades” a group of mythical beasts from Samos, Greece. *Amnhophilis* from Greek “amnos” (“*αμνόζ*”) or “*amnhos*,” archaic Greek for “lamb;” and “-*philis*” (“*φιλiζ*”), meaning “a lover of.” The name alludes to the propensity of large-bodied *Varanus* to take mammalian prey. Note, also, that the American Museum of Natural History (AMNH) is the repository for the specimen.

#### Locality and age

Mytilini Formation (Turolian, Miocene) of Samos, Greece; dated at 6.9–7.6 million years old [28] (Fig. 1).

#### Diagnosis

*Varanus* (*Varaneades*) *amnhophilis* is diagnosed by the presence of the following combination of apomorphies: Pterygoid teeth absent (Fig. 2F); Vidian canal without prootic contribution (Fig. 2G, J, M); entocarotid fossa absent (Fig. 2G, J); anterostapedial process of prootic crest distinct; undivided external facial foramen (Fig. 2I); absence of tuberal flanges of parabasisphenoid (Fig. 2G, J); crista interfenestralis extends posterolaterally, partly hiding the occipital recess in lateral view (Fig. 2G, I, J); accessory ventromedial lip on the paroccipital process present (Fig. 2G–J); dorsal and ventral tips of the parocciptial process terminate at the same mediolateral level (Fig. 2G, H, J, M); large quadrate tympanic crest (Fig. 2C); vertebrae with strong precondylar constriction (Fig. 3C).

### Description

#### Skull morphology

Only the supratemporal arch part of the right postorbitofrontal and squamosal are represented in the dorsal skull roof (Fig. 2A, B). The preserved part of each element is elongate. The squamosal bears a broadly concave postorbital facet. The postorbital is sub-ovate in cross section.

The right quadrate is well preserved and lacks only the ventral half of the tympanic crest (Fig. 2C). The straight quadrate possesses a strongly developed posterior process that quickly tapers ventrally. This process is slightly longer than the lateral development of the tympanic crest. This condition, wherein the tympanic and posterior crests are of subequal lengths, differs from the condition seen in *Varanus albigularis*, *Varanus bengalensis*, *Varanus eremeius*, *Varanus flavescens*, *Varanus gouldii*, observed *Varanus indicus*, *Varanus komodoensis*, *Varanus kordensis*, *Varanus salvadorii*, *Varanus tristis*, and *Varanus varius* among observed taxa. Those mentioned taxa possess a relatively short tympanic crest.

The saddle-shaped articular condyle is well preserved, but the epiphysis is missing from the dorsal quadrate head. There is no development of a pterygoid lappet like those seen in helodermatids and many non-anguimorph squamates. A large posterior opening of the quadrate canal is located about one-third of the way from the dorsal tip of the quadrate.

Only a small part of the right palatine (Fig. 2D) is preserved and it comes from the maxilla-palatine contact. The fragment includes most of the maxillary process, but lacks the posterior part. It preserves the short choanal groove, but not the posterior margin of the infraorbital canal, the vomerine process, or the pterygoid process.

The main body and the proximal parts of the transverse and quadrate processes of the right pterygoid are preserved. These parts confirm the presence of a columellar fossa, and a well developed and anterolaterally oriented transverse process (Fig. 2E, F). Importantly, this element confirms the absence of pterygoid teeth (Fig. 2F). Absence of pterygoid teeth is a derived condition of *Varanus* within Varanidae.

The lateral margin of the pterygoid transverse process describes a lateral curve. The anterior margin is concave. The transverse process is thickest posterolaterally. Distally, it bears posteriorly attenuated ectopterygoid facets on the dorsal and ventral surfaces. Posteromedial to the confluence of the palatine and transverse processes is a distinct basipterygoid buttress (Fig. 2F). The quadrate process is medially concave (Fig. 2E).

Most of the right prootic is preserved, but the auditory bulla and the alar crest are missing (Fig. 2G, H, J). The specimen preserves the contacts with the parabasisphenoid, basioccipital, and otooccipital. It also preserves the single (undivided) facial foramen (Fig. 2I) and the dorsal margin of the fenestra ovalis. The prootic inferior process dorsolaterally overlies the inferior process of the parabasisphenoid. The prootic-parabasisphenoid suture extends anteroventrally along the lateral surface of the inferior process. From that point, the prootic-parabasisphenoid suture extends posteriorly to the prootic-basioccipital contact, just anterior to the spheno-occipital process from which point it extends posteriorly to a point just anterior to the ventral margin of the crista interfenestralis (interfenestral crest; separating the fenestra ovalus from the occipital recess). The crista interfenestralis is developed posteroventrally rather than extending mediolaterally. The posteroventral extension of this crest partly overlaps the occipital recess, hiding its deeper parts in lateral view.

Absence of a division of the facial foramen is unusual in *Varanus* [31]. Among the 18 species for which we were able to see the facial foramen, only *Varanus acanthurus*, some *Varanus dumerilii*, *Varanus prasinus*, *Varanus rudicollis*, and *Varanus salvadorii* possess an undivided facial foramen. Noteworthy is the presence of a divided facial foramen in *Lanthanotus borneensis* [32], [33] and some *Shinisaurus crocodilurus* [34]. This character was recently discussed at some length in a description of the braincase of *Varanus priscus* [35].

**Figure 4:**
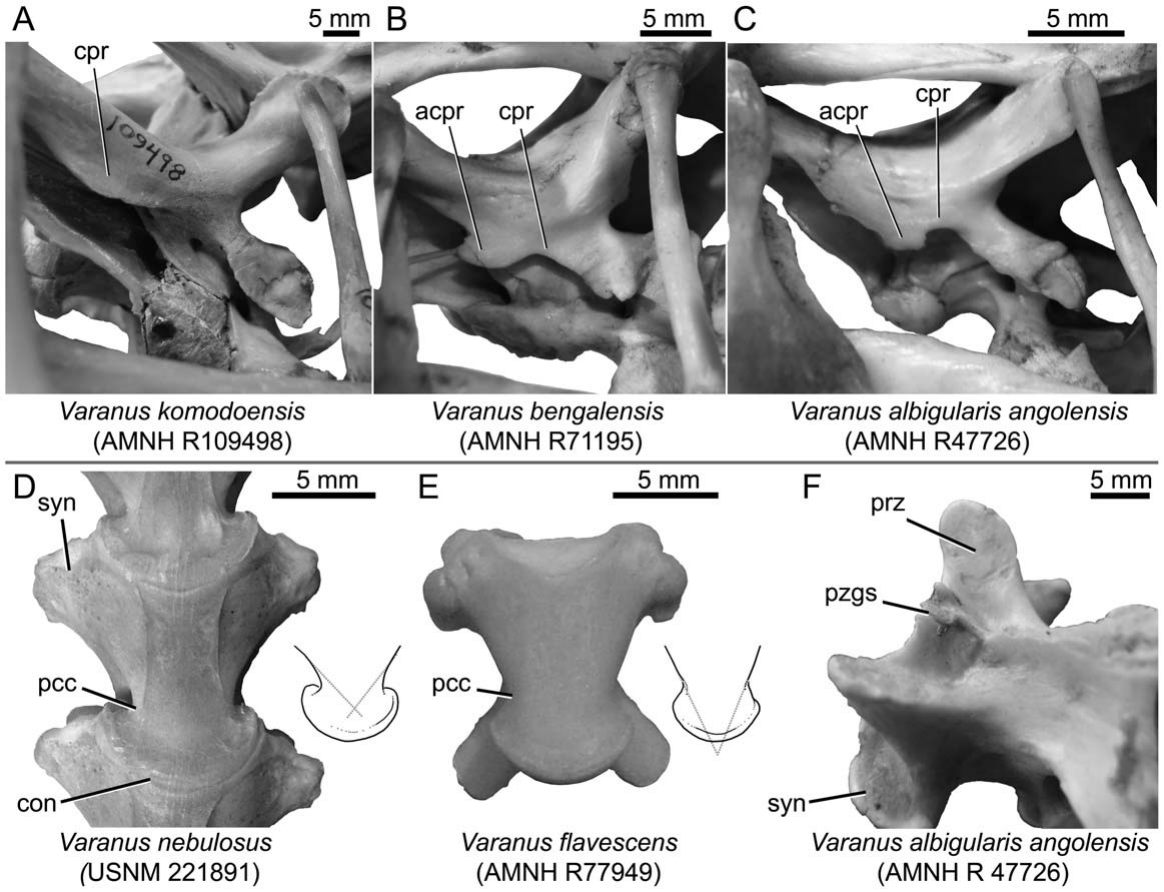
Comparative material of modern *Varanus* for anatomical comparisons with *Varanus amnhophilis*. Braincases of *Varanus komodoensis* (Australasian clade) (A), *Varanus bengalensis* (*Varanus* [*Indovaranus*] group) (B), and *Varanus albigularis* (*Varanus* [*Polydaedalus*]) group) (C). Ventral views of vertebrae of *Varanus nebulosus* (*Varanus* [*Indovaranus*] group) (D), and *Varanus flavescens* (E). (F) *Varanus albigularis* cervical vertebra in left dorsolateral view showing the pseudozygosphene. *Varanus komodoensis* (A) lacks an accessory prootic crest, *Varanus bengalensis* (B) possesses a hook-like accessory prootic crest, and *Varanus albigularis* (C) has a tabular accessory prootic crest. Insets with (D) and (E) show the strong and intermediate precondylar constrictions, respectively. Dotted gray lines show the intersection of hypothetical extensions of the ventrolateral surfaces. With a strong precondylar constriction, the lines intersect anterior to the vertebral condyle, but the intersection occurs beyond the level of the condyle in taxa with weak precondylar constriction. Abbreviations: acpr, anterostapedial process of the prootic crest; con, condyle; cpr, prootic crest (crista prootica); pcc, area of precondylar constriction; prz, prezygapophysis; pzgs, pseudozygosphene; syn, synapophysis.

The prootic crest (crista prootica) is relatively well developed anteriorly and posteriorly, but is very weakly developed at the level of the trigeminal notch. More posteriorly, near the level of the posterior margin of the spheno-occiptial tubercle and extending to a level near the posterior margin of the fenestra ovalis, the prootic crest possesses a pronounced ventrolateral flange. This process, here termed the anterostapedial process, is absent in some *Varanus* (e.g., *Varanus acanthurus*, *Varanus dumerilii*, *Varanus griseus*, and *Varanus komodoensis* [Fig. 4A], among others). When it is present, it may be expressed as a hook-like flange defining a narrow posterior concavity (e.g., in *Varanus bengalensis* [Fig. 4B]), or as an anteroposteriorly elongate tab that is laterally/ventrolaterally directed (e.g., in *Varanus albigularis* [Fig. 4C]). Because it is broken near its base, the shape of the flange is uncertain in *Varanus amnhophilis*, but the broken base attests to its original presence (Fig. 2G, I, J).

Near the posterior base of anterostapedial process, the prootic is developed into a robust paroccipital process that is strongly sutured to, but not fused with, the paroccipital process of the otooccipital (Fig. 2G, I, J). The prootic paroccipital process covers more than one-half of otooccipital paroccipital process.

Some features of the otic capsule and surrounding anatomical structures are visible on the medial view of the prootic (Fig. 2H). The medial view of the occipital recess does not preserve the medial opening to the brain cavity (the recessus scala tympani). However, much of the division between the occipital recess and the lagenar recess and vestibule is preserved, as is the lateral and much of the ventral wall of the lagenar recess. The narrow posterior connection between the lagenar recess and the preserved (lateral) part of the vestibule is preserved; the lagenar recess and the vestibule describe a “figure 8,” as preserved (Fig. 2H).

We refer to the compound structure formed by fusion of the dermal parasphenoid and the endochondral basisphenoid as the parabasisphenoid, following some recent usage [9], but differing from others in which this structure has been called the sphenoid [36], [37]. The anguimorph parabasisphenoid is often pentaradiate in ventral view with the points being formed by the parasphenoid rostrum, the basipterygoid processes, and posterolateral flanges that laterally overlie the basioccipital. However, *Varanus amnhophilis* lacks the posterolateral flanges of the parabasisphenoid (Fig. 2G, J); a condition also seen in many Indo-Asian and Indo-Australian *Varanus* (although not in members of the *Varanus prasinus*-group, *Varanus olivaceus*, or *Varanus marmoratus* and *Varanus cumingi* from the *Varanus salvator*-group). The right basipterygoid process is well preserved and extends anterolaterally as in most squamates. It is narrowest at its base with a gentle distal expansion. The expansive pterygoid facet and faces dorsolaterally.

A robust crista sellaris extends directly mediolaterally in anterior view, rather than being dorsally concave. The anterior openings of the abducens, carotid, and Vidian canals are typical of those in other *Varanus* [31], [32], [38], [39] and in *Shinisaurus crocodilurus* [32], [34], [37], [40]. Abducens canal pierces the parabasisphenoid at the base of the crista sellaris (Fig. 2H), the carotid canal lies in a deep retractor pit, and the anterior opening of the Vidian canal lies ventral to and slightly medial to the anterior opening of the abducens canal. The anterior opening of the Vidian canal lies just ventral to the level of the anterior opening of the carotid canal at the base of the basipterygoid process. A very weakly developed ridge partly divides the retractor pit. The posterior opening of the Vidian canal occurs within the body of the parabasisphenoid, just ventromedial to the anterior part of the prootic crest at the posterior base of the inferior process (Fig. 2G, J). There is no development of the entocarotid fossa associated with the recessus vena jugularis.

The inferior process of the parabasisphenoid is largely overlaid by the complementary process of the prootic (Fig. 2G, H, J, M). The prootic-parabasisphenoid suture extends posteroventrally on the inferior process, and then extends posteroventrally, just dorsal to the level of the posterior opening of the Vidian canal toward the spheno-occipital tubercle. At the base of the spheno-occipital tubercle and at an anteroposterior level lying between the trigeminal notch and the anterior facial nerve opening, the prootic-parabasisphenoid suture turns ventrally and becomes the parabasisphenoid-basioccipital suture. The ventral parabasisphenoid-basioccipital suture is mediolaterally oriented.

The right lateral part of the basioccipital is preserved. Half of the parabasisphenoid contact is preserved, as are the contacts with the right prootic, and the lateral and ventral contacts with the right otooccipital (Fig. 2G, I, J). No remnant of the occipital condyle remains. Presence of an unfinished bone surface on the spheno-occipital tubercle suggests the original presence of unfused epiphyses (Fig. 2G, I). The spheno-occipital tubercle is anteriorly located. Its posterior margin lies anterior to the anterior margin of the fenestra ovalis (Fig. 2G, J). Medial to the spheno-occipital tubercles and near the midline, the basioccipital is very robust and thickly developed. The posterior, basioccipital-otooccipital suture extends anteromedially from the spheno-occipital tubercle except where it nears the midline and is posteriorly expanded. However, the state of preservation does not allow further characterization of that suture, or of the bone.

The fused exoccipital-opisthotic unit is here referred to as the otooccipital, following recent usage [34], [37], [41]. The mostly complete right otooccipital is preserved, lacking only the occipital condyle, the margins of cranial nerves X–XII, and more medially occurring structures (Fig. 2G–J).

The otooccipital constitutes the dorsal part of the posterolateral braincase walls. Anteriorly, it preserves most of the crista interfenestralis, which separates the fenestra ovalus from the occipital recess and demonstrates that this crest possesses the posterolateral expansion described above. The contact between the prootic and the otooccipital is vertical and extends from the spheno-occipital tubercle dorsally to the crista interfenestralis. The crista interfenestralis extends posteriorly and slightly posterodorsally from a point dorsal to the posterior margin of the spheno-occipital tubercle to the paroccipital process (Fig. 2I).

The crista tuberalis extends posterodorsally from the spheno-occipital tubercle as in other *Varanus*. As in many *Varanus* (but not *Varanus griseus* and *Varanus acanthurus*), the crista tuberalis possesses a distinct, ventrolateral, paroccipital tuberosity (Fig. 2G–J). The paroccipital tuberosity of *Varanus amnhophilis* is not developed into a finger-like flange as it is in *Varanus bengalensis*. It is located near the base of the paroccipital process and is directed ventrally/ventrolaterally.

#### Mandibular morphology

Preserved mandibular remains for *Varanus amnhophilis* consist of parts of the right coronoid, surangular, and prearticular-articular complex (Fig. 2K–M). The coronoid has a short and gently ventrally concave coronoid eminence similar to that of other *Varanus*. Similarly, the preserved part of the surangular and prearticular-articular preserve no autapomorphic characteristics and are similar to those elements in most other *Varanus*.

#### Vertebrae

Parts of three cervical and three complete dorsal vertebrae are known (Fig. 3). Based on comparisons of form between the synapophyses and hypapophyses of *Varanus amnhophilis* and extant forms, we interpret the preserved cervical vertebrae as cervicals 3, 4, and the anterior part of 5. Comparisons of the shapes of the synapophyses between *Varanus amnhophilis* and extant forms suggest that the preserved dorsal vertebrae are posterior dorsals; the first of the three is probably presacral vertebra 23, 24, or 25.

The cervical vertebrae have relatively tall and anteroposteriorly narrow neural spines and well-developed hypapophyseal pedicles capped by hemispherical hypapophyses (intercentra) (Fig. 3A). The dorsal vertebrae are also of a typical *Varanus* form in the short and broad neural spines, absence of zygosphenes-zygantra and pseudozygosphenes (Fig. 3B), and the presence of precondylar constriction (Fig. 3C). The precondylar constriction is noteworthy in that it is pronounced as compared to the more moderately constricted centra of taxa such as members of the *Varanus* (*Polydaedalus*) clade and *Varanus flavescens* (Fig. 4E). The minimum precondylar centrum width is approximately 76.5 percent of the maximum condylar diameter in the second preserved dorsal vertebra and approximately 75 percent of the maximum condylar diameter in the first preserved dorsal vertebra.

Zygosphenes-zygantra or structures similar to them (pseudozygosphenes) are characteristic of some varanids, such as *Saniwa ensidens* [11], [12]. However, pseudozygosphenes also occur in *Varanus exanthematicus* and *Varanus albigularis* (Fig. 4F).

## Materials and Methods

### Phylogenetics

Our phylogenetic analysis includes a subset anguimorph species from a recent combined-evidence of Anguimorpha [3] and includes *Shinisaurus crocodilurus* as a representative shinisaur outgroup (Dataset S1). Five mosasaurs (*Adriosaurus suessi*, *Aigialosaurus dalmaticus*, *Coniasaurus crassidens*, *Dolichosaurus longicollis*, and *Pontosaurus lesinensis*) were chosen to represent the basal condition in that clade, based on their position in recent phylogenetic analyses [2], [3], [42]–[46]. Other taxa were included because they are more closely related to *Varanus* than to *Shinisaurus crocodilurus*. These taxa included *Paravaranus angustifrons*, *Proplatynotia longirostrata*, *Lanthanotus borneensis*, *Cherminotus longifrons*, *Aiolosaurus oriens*, *Ovoo gurvel*, and the necrosaurs ‘*Saniwa*’ *feisti*, *Necrosaurus cayluxi*, *Necrosaurus eucarinatus*, *Saniwides mongoliensis*, and *Telmasaurus grangeri*. A full list of comparative material can be found with the (Text S1).

In addition to taxa included in the earlier analysis [3], we added some relevant fossils. These included a partial skull referred to ‘*Varanus*’ *marathonensis* [12], [16], [47] and the original holotype *Varanus marathonensis* vertebra [12], both from Europe. We have treated the partial skull separate from the presumed holotypic vertebra because there is no clear reason to associate them. We added data from the incompletely known, subfossil *Varanus hooijeri* from Flores. In his review of varanids and ‘megalanids’, de Fejérváry [48] described a fossil he believed to be close to *Varanus bengalensis*, which appears here as *Varanus* cf. *bengalensis*. We also include the recently described Birket Qarun ‘*Varanus*’ [49], and a putative basal *Varanus* or *Varanus* outgroup from the Jebel Qatrani Formation (the Yale Quarry ‘varanid’) [24].

Characters, character states, and character state codings are largely from Conrad et al. [3], with some modifications to *Varanus amnhophilis* (AMNH FR 30630) based on additional preparation of the specimen and further study of the material (see the description above). We provide the morphological codings for the matrix below in the (Dataset S1). The molecular character coding is exactly as it was in the earlier study [3] and is derived largely from the work of Ast [50], [51].

In addition to the morphological character matrix used by Conrad et al. [3], we add 71 morphological characters (Text S2; characters 423–493). Note that, as with the Conrad et al. [3] and other earlier iterations of this data matrix [2], [4], [52], the biogeography character (character 364) was not used in phylogenetic hypothesis reconstruction. Note, also, that as in Conrad et al. [3], characters 413 and 414 replace character 236 (deactivated here) and character 415 replaces character 242 (deactivated here). Thus, of the 493 non-molecular characters in the matrix, only 489 were used in the tree searches. We performed a phylogenetic analysis of 83 species scored for 489 morphological and 5733 molecular characters.

We used *NEXUS Data Editor* (*NDE*) [53] to assemble and manage the data matrix. We performed an analysis using the New Technology Search in the computer program *T.N.T: Tree analysis using new technology* [54] (1000 replicates) with “ratchet” and “drift” options employed.

### Paleobiogeography

After tree construction and consensusing, we mapped biogeographic data in the computer program *Mesquite* [55] and reconstructed the ancestral distributions in a basic parsimony analysis.

### Size estimation/reconstruction

**Figure 5:**
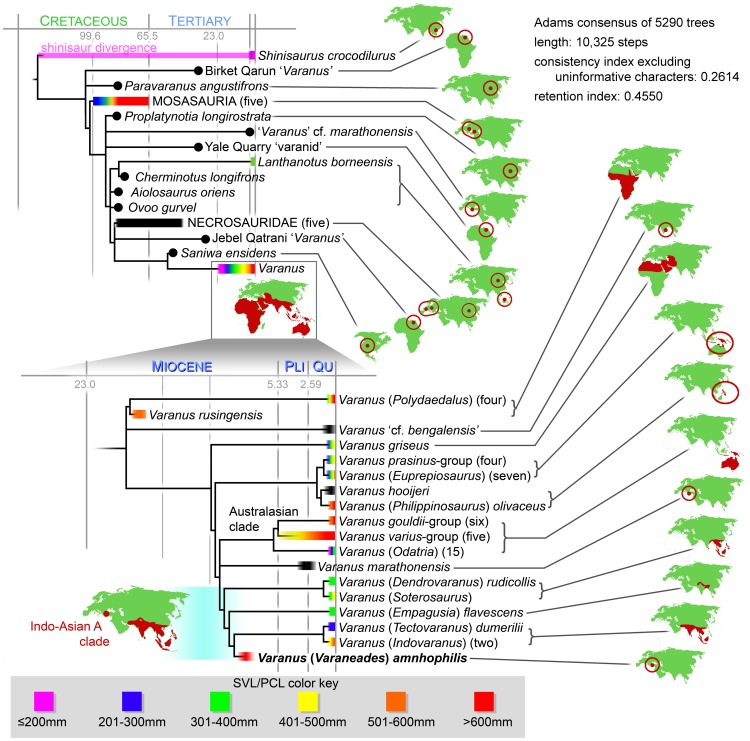
Temporally calibrated phylogeny of varanids and their outgroups. Size data are indicated by color included on the known temporal ranges are derived from published accounts [21], ranges in black indicate taxa without reliable size data. Extant *Shinisaurus* was used as an outgroup for tree reconstruction, but the shinisaur clade is homogenous in size and extends into the Cretaceous [3]. Some nodes collapsed for space considerations, but the number of included species is in parentheses next to the taxon name (Dataset S1). Maps present the known distributions of the indicated taxa in red. Mosasaur distribution is based on the five basal taxa included in the analysis. *Varanus amnhophilis* is a nested member of the Indo-Asian A clade and the discordant distribution of that taxon with respect to other Indo-Asian A taxa is illustrated by the map on the lower left.

The holotype of *Varanus amnhophilis* consists of cranial, mandibular, and vertebral elements, along with one pectoral fragment (Figs. 2, 3). Having associated skull and postcranial is unusual among fossil varanids, which are often known from isolated vertebrae or long bone ends [12], [16], [24], [49]. Because the AMNH FR 30630 is known from a relatively complete braincase and from cervical and dorsal vertebrae, we measured those elements in 20 specimens of 15 extant species of *Varanus* (Table 1) representing all of the major *Varanus* clades (see Fig. 5). The measured dorsal vertebrae come from just anterior to the lumbar vertebra in each specimen (posterior pelvic dorsal vertebrae of recent usage [56]).

We ran regressions of these data against precaudal lengths in those specimens and calculated standard deviations for these regressions. We assumed that a lower standard deviation implied a better correlation between any principal measurement and the precaudal length.

Precaudal lengths (PCL) was used as a proxy for body size because the tails of monitor lizards make up a variable amount of their total body length; that is, some monitors (e.g., *Varanus komodoensis*) have relatively short tails compared to their overall lengths, whereas others (e.g., *Varanus salvator*) have relatively long tails [21], [23]. Precaudal length is similar to snout-vent length (SVL), a measurement often used as a proxy for reptile size. Precaudal length was used instead of SVL here because SVL data are not always available for skeletonized specimens and are not available for most fossil taxa. We measured skeletons from the tip of the snout to the midline tip of the ventral centrum surface (not including the articular surface of the condyle) to determine the PCL of the extant specimens.

We also took measurements of lateral braincase length (BCL) and length of posterior dorsal vertebrae (DVL). Lateral braincase length is measured as the length from the anterior tip of the basipterygoid process to the posterolateral tip of the paroccipital process. Dorsal vertebra length was measured from the anterior limit of the centrum at the midline (thus, the middle of the middle of the ventral cotylar lip, a gentle posterior embayment at the anterior end of the centrum) to the posteromedial condylar lip (not extending onto the articular surface of the condyle).

For estimating sizes based on dorsal vertebrae, we used the data in Table 1 along with measurements DVL and BCL of *Varanus amnhophilis* and *Varanus priscus* (designated here as “measurement of interest”; measurements in Results, below). The equation is as follows:

measurement of interest * (average PCL for relevant extant taxa/average measurement of interest for relevant extant taxa)

For the purposes of our study, we include only non-snake squamates (‘lizards’) for size comparison. Snakes (Serpentes) are, of course, just one of many radiations of ‘lizards’ that show extreme limb reduction and limblessness. However, snakes are much more speciose and diverse than any other single group of limbless squamates. It is noteworthy that snakes include some of the longest extant reptiles—certainly the longest extant squamates, suggesting that they (or some sub-group of them) may have evolutionary innovations causing them to operate under a slightly different set of biological pressures. Although there is the lingering idea that snakes may be closely related to varaniform squamates [57] and, consequently, may share this such innovations with them, still this connection is tenuous [2], [58]–[61] and the innovation(s) (if any) remain unknown. For these reasons, snakes are excluded, hereafter, from comparisons with ‘lizards’ for the remainder of this paper.

## Results

### Phylogeny

The shortest tree-length recovered by the analysis had a length of 10,325 steps, consistency index excluding uninformative characters of 0.2614, and a retention index of 0.4550 (Fig. 5). The analysis found 5290 trees of that length and none shorter.

Because we included some very fragmentary taxa, a strict consensus of our analysis recovered little resolution (Text S3). Following some recent studies [2], [3], [62]–[64], we report the Adams consensus (Fig. 5) because it shows the relationships that are common to all trees and collapses volatile taxa to the level of their least inclusive node. Although species-level coding was used for all of the included taxa, some clades were collapsed in the interest of brevity in the figure (Fig. 5). Full strict and Adams consensus trees are available with the (Text S3).

Our analysis recovers a *Saniwa*-*Varanus* clade exclusive of the Eocene-Oligocene varanids from Egypt (see below and Fig. 5). Of the seven unambiguous *Varanus* synapomorphies recovered in this analysis, *Varanus amnhophilis* may be demonstrated to possess absence of pterygoid teeth (Fig. 2F) and absence of accessory zygosphene-like vertebral processes (Fig. 3B). The clade referred to as Indo-Asian A [50] and the Australasian clade are united (Fig. 5) by one morphological (presence of strong precondylar constriction of the presacral vertebrae) and 18 molecular unambiguous character states. Indo-Asian A, including *Varanus amnhophilis*, is united by a Vidian canal completely housed by the parabasisphenoid (Fig. 2G) and 28 unambiguous molecular synapomorphies. Members of Indo-Asian A exclusive of the *salvator*/*rudicollis*-group are united by absence of posterolateral parabasisphenoid flanges and 12 unambiguous molecular synapomorphies. The latter clade, exclusive of *Varanus flavescens*, is united by presence of separate ossifications on the spheno-occipital epiphyses. *Varanus dumerilii* and *Varanus* (*Indovaranus*) spp. are united to the exclusion of *Varanus amnhophilis* by the presence of a prootic contribution to the posterior Vidian canal opening and a ventrolaterally expanded paroccipital process tubercle.

“*Varanus*” *sivalensis* is a poorly known taxon represented only by a fragmentary humerus and two vertebrae [65], the latter lacking precondylar constriction. This taxon is usually considered to represent an early monitor lizard [12], [27], [65]. Although these remains represent a very large squamate, possibly a varaniform, they show no special similarity with *Varanus* and were not included in the current analysis.

### Paleobiogeography

An African origin for *Varanus* has been suggested based, in part and most recently, on some Egyptian Paleogene fossils attributed to *Varanus* or its ‘stem’ (i.e. Varaninae) [24], [49]. However, these fossils were not previously included in a phylogenetic analysis, and our analysis suggests that there is no compelling evidence supporting their inclusion within the *Saniwa*-*Varanus* dichotomy (Fig. 5). Earlier studies suggested an Asian origin for *Varanus* based on known biogeographic distributions of fossils and phylogeny of extant taxa based on karyological data [66]–[68] and 12S rRNA [69].

Many of the most proximal *Varanus*-outgroups come from across Eurasia or North America [2], [70] (Fig. 5), leaving the biogeographic position of the *Varanus* outgroup unresolved. *Varanus rusingensis* and the extant clade *Varanus* (*Polydaedalus*) spp. together constitute the basal-most *Varanus* radiation and occur in Africa. *Varanus griseus* is recovered as a phylogenetic intermediate between the *Polydaedalus*-group and other *Varanus*, and is known from Africa and western Asia. Other *Varanus* come from eastern Asia and Australia [21] (Fig. 5). Thus, we reconstruct the ancestral biogeographic region of *Varanus* as Asia.

### Body size estimation

The average PCL/BCL ratio for all *Varanus* in which those measurements were available is 17.68 with a standard deviation (SD) of 2.322 (y intercept [yi] = 17.16; x intercept [xi] = 14.11; R^2^ = 0.956). The PCL/BCL for *Varanus komodoensis* is 16.76 (SD of 0.474) (note, only two data points; yi = 17.67; xi = −59.67), and for the Indo-Asian A clade it is 18.57 (SD of 2.73; yi = 22.08; xi = −85.53; R^2^ = 0.918).

The average PCL/DVL for all measured *Varanus* is 37.84 (SD of 3.791; yi = 39.93; xi = −24.20; R^2^ = 0.975). The average PCL/DVL for *Varanus komodoensis* is 39.93 (SD of 2.309; yi = 44.71; xi = 131.4) and for the Indo-Asian A clade it is 36.91 (SD of 2.562; yi = 42.21; xi = −64.96; R^2^ = 0.994).

The DVL of *Varanus amnhophilis* is 18 mm; its BCL is 38.38 mm. The DVL of *Varanus priscus* (AMNH FR 1486) is 53 mm. The BCL of *Varanus priscus* (BMNH 39965) is estimated at 106 mm based on an incomplete braincase [35].

**Figure 6:**
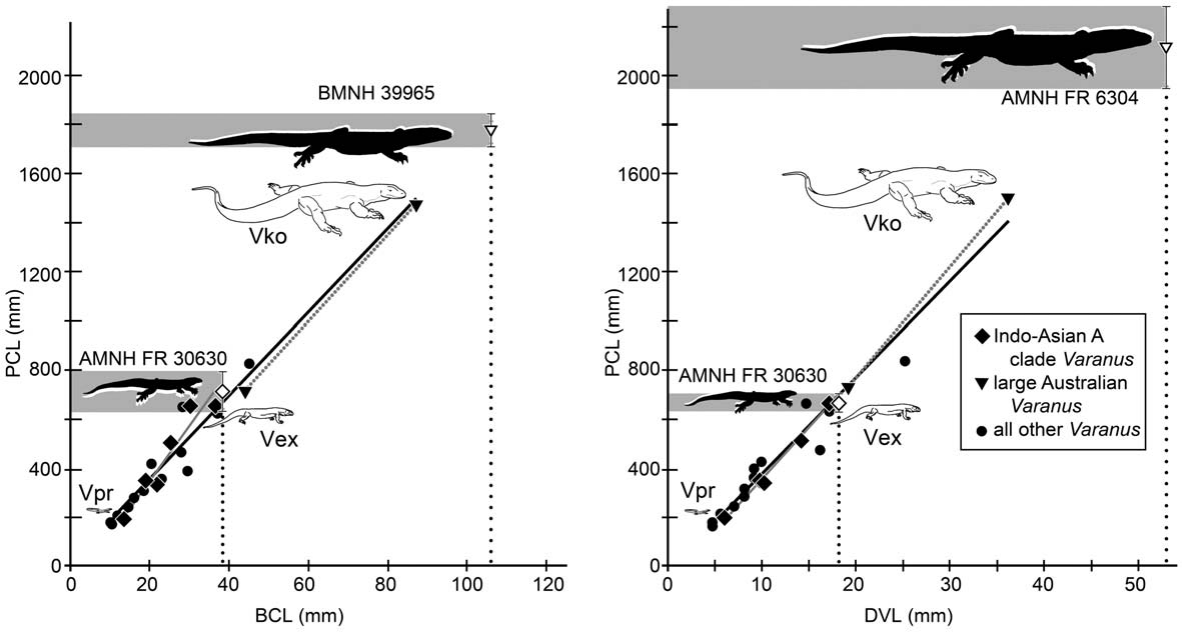
Size estimates for *Varanus* (*Varaneades*) *amnhophilis* (AMNH FR 30630) and Megalania (*Varanus priscus*, BMNH 39965 and AMNH FR 6304) based on comparisons of lateral braincase length (BCL) and dorsal vertebral length (DVL). The open diamonds indicate *Varanus* (*Varaneades*) *amnhophilis* and the open triangles indicate *Varanus priscus*. The dotted trend lines was calculated using *Varanus komodoensis* (PCL/BCL, y intercept [yi] = 17.67, x intercept [xi] = −59.67; PCL/DVL, yi = 44.71, xi = 131.4). The solid gray trend line was calculated using extant species from the Indo-Asian A clade of *Varanus* (PCL/BCL, yi = 22.08, xi = −85.53, R^2^ = 0.918; PCL/DVL, yi = 42.21, xi = −64–96, R^2^ = 0.994). Solid black trendline was calculated using all the data (PCL/BCL, yi = 17.16, xi = 14.11, R^2^ = 0.956; PCL/DVL, yi = 39.93, xi = −24.20, R^2^ = 0.975). Open line drawing represents *Varanus prasinus* (Vpr), the medium represents *Varanus* (*Polydaedalus*) *exanthematicus* (Vex), and the large represents *Varanus komodoensis* (Vko)—all to scale. Gray bars indicate predictive size range within 95 percent confidence interval. See text and (Text S1 and Text S3).

Correlations between morphological dimensions and body size within extant *Varanus* allow us to estimate PCL in our new fossil (Tables 1, 2, 3). We found strong correlations between BCL and PCL, and between DVL and PCL in modern *Varanus* (Table 1). Using these metrics and supposing that *Varanus amnhophilis* scaled similarly to other “Indo-Asian A”-clade *Varanus*, we estimate its PCL at approximately 712.6 mm (±83.96 mm with a 95 percent confidence interval) based on braincase length, or approximately 664.5 mm (±36.89 mm) based on dorsal vertebra length (Fig. 6) (Table 3). Based on compiled size data for modern *Varanus* [21], *Varanus amnhophilis* is a giant among monitor lizards, being larger than approximately 85% of known *Varanus* (Table 2) and, therefore, the vast majority of known lizards (below).

**Table 3:** FOSSIL BODY SIZE ESTIMATES.

taxon	comparisons	meas.	L (mm)	PCL est. (mm)	±
*Saniwa ensidens*	all *Varanus*	DVL	10.9	412	17.66
*V. amnhophilis*	all *Varanus*	BCL	38.38	678.64	38.12
*V. amnhophilis*	IA-A	BCL	38.38	712.6	83.96
*V. amnhophilis*	all *Varanus*	DVL	18	681.1	29.19
*V. amnhophilis*	IA-A	DVL	18	664.5	36.89
*V. priscus* A	all *Varanus*	BCL	106	1874	105.3
*V. priscus* A	*Vko* and *Vgo*	BCL	106	1897	456.1
*V. priscus* A	*V. komodoensis*	BCL	106	1777	69.63
*V. priscus* B	all *Varanus*	DVL	53	2005	85.94
*V. priscus* B	*Vko* and *Vgo*	DVL	53	2170	211.2
*V. priscus* B	*V. komodoensis*	DVL	53	2116	169.6

Body size estimates for fossil varanids (*Saniwa ensidens*, *Varanus amnhophilis*, and two specimens of *Varanus priscus*). Estimates derived from comparisons with the data presented in Table 1, as described in the text. Headings/abbreviations: taxon, fossil taxon whose size is predicted; comparisons, taxon group used for making the length estimation; meas., measured element; L, observed length of the measured element in mm; PCL est., estimated precaudal length of the fossil taxon; ±, the difference between the estimated PCL length and the maximum or minimum length falling within a 95 percent confidence interval; BCL, lateral braincase length (see text); DVL, dorsal vertebra length (see text), *V.*, *Varanus*; *Vko*, *Varanus komodoensis*; *Vgo*, *Varanus gouldii*. Underlined measurements indicate those which were deemed most pertinent based on taxonomic comparisons and phylogenetic placement (Fig. 5) and illustrated in Figure 6.

Among extant non-*Varanus* lizards, only *Amphisbaena alba* reaches giant sizes and appears to average over 600 mm PCL or SVL [71]. However, the body size of *Amphisbaena alba* and the large, limbless anguid, *Pseudopus apodus* (which may exceed 500 mm SVL [72], [73]) is smaller than a *Varanus* with a similar SVL. Among living, limbed lizards, *Cyclura* (which may exceed 500 mm SVL [74]) includes the largest species after *Varanus*. Iguanidae includes several other large-bodied species belonging to the clades *Amblyrhynchus*, *Conolophus*, *Ctenosaura*, *Cyclura*, *Iguana*, and the fossil taxon *Lapitiguana* that approach or exceed 500 mm SVL [75], [76]. Limbed lizards, particularly the partly- to heavily-herbivorous iguanids, are thicker-bodied than the elongate limbless forms, and may, therefore, have greater masses than said limbless forms or the mostly predatory *Varanus*.

Polyglyphanodontidae includes some of the largest fossil lizards (AMNH, USNM, and YPM collections include specimens with PCLs of more than 410 mm [pers obs. of the authors]). However, except for the mosasaurs, which were marine and could reach total lengths of as much as 17 m [77], no other clade of lizards exhibit giant average sizes (>600 mm) common to several species of *Varanus* (Figs. 5, 6; Tables 1, 2).

*Varanus amnhophilis* has a PCL greater than approximately 99% of known, non-snake, squamates ever.

We also estimated the size of *Varanus priscus* ( = *Megalania prisca*; Megalania [35]). Given that Megalania is a nested member of the large-bodied Australasian *Varanus* clade, the largest specimen of *Varanus priscus* available to us [AMNH FR 6304 (cast); dorsal vertebra length = 52.97 mm], we estimate its PCL at 2116 mm (±169.6 mm) (Fig. 6A). A recently redescribed braincase [35] comes from an individual whose PCL we estimate at 1777 mm (±69.63 mm) (Table 3) (Fig. 6B). By employing the size-reconstruction metrics of a earlier analysis [13], a recent study suggested that *Varanus priscus* would have had an average total length of approximately 3450 mm [25] (presumably suggesting a SVL of 1725 mm or less given the scaling of *Varanus komodoensis* and *Varanus varius*). Our metric and the available *Varanus priscus* data suggest that at least AMNH FR 6304 was much larger than that, but considerably smaller than many historical estimates [13], [15], [78]. It is, however, noteworthy that the largest-known wild specimens of some species may far exceed the mean or average size. For example, very large *Varanus exanthematicus* may be 151 percent of the mean, large *Varanus rudicollis* may be 175 percent of the mean, *Varanus komodoensis* may be 181 percent of the mean, and the largest *Varanus salvator salvator* measured nearly 225 percent of the mean size for the subspecies [21], [79] (Table 2), suggesting that exceptionally large specimens of *Varanus priscus* might have exceeded 3000 mm PCL. This is similar to some of the larger body-size estimates for the species. It is also possible that Megalania is a species-complex, not a species [27], but that little influences our size estimates.

These metrics are also applicable to proximal *Varanus* outgroups. The posteriormost 10 dorsal vertebrae of a specimen of *Saniwa ensidens* with an SVL of 420 mm, average 10.89 mm in length [11]. Assuming *Saniwa ensidens* scales similarly to *Varanus* generally, our model predicts a PCL of approximately 412.0 mm (±17.66 mm)—within two percent of the reported SVL [11]. Consequently, we believe that our method for estimating varanine sizes from isolated vertebrae has considerable predictive power for varanines generally, at least within the *Varanus*-*Saniwa* dichotomy.

## Discussion

*Varanus rusingensis* (the Rusinga Monitor) and *Varanus amnhophilis* (the Samos Dragon) are the earliest-known demonstrable *Varanus*. *Varanus amnhophilis* offers the first evidence of a Miocene divergence for major *Varanus* clades beyond the basal dichotomy between the African *Polydaedalus* and all other true monitors (Fig. 5). Given the phylogenetic hypothesis recovered here, no fewer than eight major *Varanus* lineages must have been present by the Turolian. These radiations include groups of Indo-Asian A monitors (the *Empagusia*, *Indovaranus*, and *Tectovaranus* clades), the *Dendrovaranus*-*Soterosaurus* clade, the Australasian clade, the Indo-Asian B clade, the lineage including *Varanus griseus*, and the *Polydaedalus* clade.

In addition to the morphological diversity suggested by the presence of these clades, we hypothesize a broad distribution for *Varanus* across Africa, Arabia, Eastern Europe, and Asia by the late Miocene. This is further supported by fragmentary material of uncertain phylogenetic affinity, such as *Varanus* ‘cf. *bengalensis*’, *Varanus marathonensis*, and others [12], [16], [24], [47]–[49]. As opposed to the recently favored out-of-Africa hypothesis [24], [49], we suggest a Laurasian origin for *Varanus* with subsequent major diversification perhaps occurring in southern Asia.

As with some recent analyses of *Varanus* [3], [50], [51], [80], [81], our data support a basal dichotomy, perhaps representing a vicariant split, between the *Polydaedalus* clade and all other *Varanus*. However, our analysis differs from some recent molecular analyses [50], [51], [80], [81] in that *Varanus griseus* is not found to be a part of *Polydaedalus* radiation. Given this hypothesis, the distribution of *Varanus griseus* requires some further attention. One interpretation would be that *Varanus griseus*, like all non-*Polydaedalus* monitors, is of a primarily Asian origin with a later invasion of northern Africa. Aridification during the Miocene, which probably made the Sahara a more significant biogeographic barrier [82], [83], led to the eventual isolation of the southern African clade. Occurrence of *Varanus yemenensis* (which was not included in our phylogenetic analysis, but is very similar to *Varanus albigularis* and *Varanus exanthematicus*) on the Arabian Peninsula would represent a dispersal across the Red Sea, perhaps at its southern end where Arabia approaches Africa and the Indian Ocean.

An alternate interpretation would be that the out-of-Africa hypothesis is correct. If *Varanus* is of African origin and subsequently dispersed northward and eastward to its present range, *Varanus griseus* and its lineage might represent an early emigration. However, this would require complete extirpation of non-*Polydaedalus Varanus* from Africa, with no remnant of their presence. Our data more strongly support the Eurasian origin for *Varanus*.

Varanidae originated in the Cretaceous and soon exploited small body-size ecomorphs [4], something they still do today with no fewer than nine species averaging below 150 mm in SVL [21], but it is only since the late Miocene that there is any evidence of giant varanids (Fig. 5). Lizard gigantism and dwarfism is thought to be influenced by a complex set of pressures and is often associated with island endemism [26], [84], [85]. Today, the largest lizards have a variety of ways of dealing with mammalian competitors. The only giant, non-*Varanus* lizards alive today (*Amphisbaena alba*) are limbless and have adopted some degree of fossoriality. *Cyclura* evolved large (if not giant) sizes on isolated islands. This is similar to some of the larger varanids, including *Varanus komodoensis*, *Varanus salvator*, and *Varanus mabitang*.

*Varanus* is unique in that it contains giant lizards that co-occur with endemic mammalian competitors. Numerous species of *Varanus* appear alongside marsupials in Australia [21], [23], [86] and this clade likely developed gigantism on mainland Asia [27]. However, the largest remaining members of the clade today live on isolated islands and on Australia where eutherians are human-introduced newcomers. It is only in Africa that giant *Varanus* (*Varanus* niloticus and *Varanus ornatus*) co-occur with numerous eutherian mammals similar to the competitors *Varanus amnhophilis* would have encountered [29], [30].

Boidae and Pythonidae include the longest and heaviest extant squamate species [22], [87], [88]. Locomotion, hunting, and feeding in these snakes is different from that of ‘lizards,’ even limbless lizards, perhaps offering a different set of parameters for producing giants. Boids reached their largest known sizes long before varanids, but they seem to have done so in the absence of significant eutherian competitors [87]. Even so, modern boids and pythonids reach great size in the presence of eutherian competitors in the tropics worldwide today [22], [88]. Without the benefit of mammalian-style parental protection, young and small members of large-bodied lizard and snake species must use camouflage, water, burrows, and/or trees to escape predators, and/or grow to adulthood relatively quickly [22], [79].

Geological data from Samos suggest that *Varanus amnhophilis* lived in an environment including mixed forest and open areas with freshwater streams, and possibly strong seasonality including some flooding [29], [30]. Modern members of the larger clade to which *Varanus amnhophilis* belongs (also including *Varanus dumerilii*, *Varanus flavescens*, *Varanus rudicollis*, the *Indovaranus*-group, and the *Soterosaurus*-group) are largely terrestrial, but are often strong climbers and swimmers [21].

The Samos Dragon, *Varanus amnhophilis*, shared its environment with mustelids (e.g., *Promeles*, *Parataxidea*, and *Promephitis*), hyaenids (e.g., *Ictitherium* and *Hyaenicitis*, among others), and suids (*Microstonyx*) [29], [30]. Each of these mammals would have posed significant threats to a monitor lizard and/or would have been predators on *Varanus* eggs. Despite this, *Varanus* (*Varaneades*) *amnhophilis* is the earliest-known giant limbed lizard and the first to establish the upper one-percent of lizard sizes on land.

## Supporting Information

DATASET S1 **MORPHOLOGICAL CHARACTER-BY-TAXON MATRIX.** Here, we include the full morphological data matrix, including the character scorings for those characters described in earlier analyses. Note that some taxa were coded for only molecular characters (see text). Those taxa have a “?” for each coding but are included here for ease of the reader should she or he desire to reproduce our matrix.
(DOC)

TEXT S1 **COMPARATIVE MATERIAL.** Observations on the following specimens were used for this study. Institutional abbreviations: AMNH, American Museum of Natural History; BSP, Bayerische Staatssammlung für Paläontologie und Geologie; BMNH PR, Natural History Museum, London (Great Britain); GM, Geiseltal Museum of the Martin-Luther Universität in Halle/Saale (Germany); UF, University of Florida, Florida State Museum; ZPAL, Zoological Institute of Paleobiology, Polish Academy of Sciences, Warsaw (Poland).
(DOC)

TEXT S2 **CHARACTERS USED IN THE PHYLOGENETIC ANALYSIS.** Morphological characters and character-states used in the phylogenetic analysis.
(DOC)

TEXT S3 **FULL CONSENSUS TREES FOR THE ANALYSES.** Below we include the strict and the Adams consensus results for the results of the TNT [54], [89], [90] analyses as read out by PAUP* [91]. Note that the strict consensus lacks resolution because of the volatile nature of some taxa (e.g., Jebel Qatrani ‘*Varanus*’).
(DOC)

## References

[1] Gao K-Q, Norell MA (2000) Taxonomic composition and systematics of Late Cretaceous lizard assemblages from Ukhaa Tolgod and adjacent localities, Mongolian Gobi Desert. Bulletin of the American Museum of Natural History 249: 1–118.

[2] Conrad JL (2008) Phylogeny and systematics of Squamata (Reptilia) based on morphology. Bulletin of the American Museum of Natural History 310: 1–182.

[3] Conrad JL, Ast JC, Montanari S, Norell MA (2011) A combined evidence phylogenetic analysis of Anguimorpha (Reptilia: Squamata). Cladistics 27: 230–277. 10.1111/j.1096-0031.2010.00330.x 34875778

[4] Norell MA, Gao K-Q, Conrad JL (2008) A new platynotan lizard (Diapsida: Squamata) from the Late Cretaceous Gobi Desert (Ömnögov), Mongolia. American Museum Novitates 3605: 1–25.

[5] Gao K-Q, Fox RC (1996) Taxonomy and evolution of Late Cretaceous lizards (Reptilia: Squamata) from western Canada. Bulletin of Carnegie Museum of Natural History 33: 1–107.

[6] Conrad JL (2004) Re-analysis of anguimorph (Squamata: Reptilia) phylogeny with comments on some problematic taxa. Journal of Vertebrate Paleontology 24 (suppl. 3) 47A.

[7] Balsai MJ (2001) The phylogenetic position of *Palaeosaniwa* and the early evolution of platynotan (varanoid) anguimorphs [Ph.D. dissertation]. Philadelphia: University of Pennsylvania. 253 p.

[8] Norell MA, Gao K-Q (1997) Braincase and phylogenetic relationships of *Estesia mongoliensis* from the Late Cretaceous of the Gobi Desert and the recognition of a new clade of lizards. American Museum Novitates 3211: 1–25.

[9] Conrad JL, Rieppel O, Gauthier JA, Norell MA (2011) Osteology of *Gobiderma pulchrum* (Monstersauria, Lepidosauria, Reptilia). Bulletin of the American Museum of Natural History 362: 1–89.

[10] Gilmore CW (1928) Fossil lizards of North America. Memoirs of the National Academy of Sciences 22: 1–197.

[11] Rieppel O, Grande L (2007) The anatomy of the fossil varanid lizard *Saniwa ensidens* Leidy, 1870, based on a newly discovered complete skeleton. Journal of Paleontology 81: 643–665.

[12] Estes R (1983) Sauria terrestria, Amphisbaenia. New York: Gustav Fischer Verlag. 249 p.

[13] Hecht MK (1975) The morphology and relationships of the largest known terrestrial lizard, *Megalania prisca* Owen, from the Pleistocene of Australia. Proceedings of the Royal Society of Victoria 87: 239–249.

[14] Molnar RE (1990) New cranial elements of a giant varanid from Queensland. Memoirs of the Queensland Museum 29: 437–444.

[15] Molnar RE (2004) Dragons in the dust: the paleobiology of the giant monitor lizard *Megalania*; Farlow JO, editor. Bloomington: Indiana University Press. 211 p.

[16] Molnar RE (2004) The long and honorable history of monitors and their kin. In: Pianka ER, King DR, King RA, editors. Varanoid Lizards of the World. Bloomington, IN: Indiana University Press. pp. 10–67.

[17] Pianka ER (2004) Introduction. In: Pianka ER, King DR, editors. Varanoid Lizards of the World. Bloomington, IN: Indiana University Press. pp. 3–9.

[18] Molnar RE, Pianka ER (2004) Biogeography and phylogeny of varanoids. In: Pianka ER, King DR, King RA, editors. Varanoid Lizards of the World. Bloomington, IN: Indiana University Press. pp. 68–76.

[19] Welton LJ, Siler CD, Bennett D, Diesmos A, Duya MR, et al (2010) A spectacular new Philippine monitor lizard reveals a hidden biogeographic boundary and a novel flagship species for conservation. Biology Letters published online 7 April 2010: 1–5. 10.1098/rsbl.2010.0119 PMC2936141 20375042

[20] Koch A, Auliya M, Ziegler T (2010) Updated checklist of living monitor lizards of the world (Squamata: Varanidae). Bonn zoological Bulletin 57: 127–136.

[21] Pianka ER, King DR, King RA, editors (2004) Varanoid Lizards of the World. Bloomington and Indianapolis, Indiana Indiana University Press. 588 p.

[22] Pianka ER, Vitt LJ (2003) Lizards: windows to the evolution of Diversity. Berkeley: University of California Press. 346 p.

[23] Pianka ER (1995) Evolution of body size: varanid lizards as a model system. American Naturalist 146: 398–414.

[24] Smith KT, Bhullar B-AS, Holroyd PA (2008) Earliest African record of the *Varanus* stem-clade (Squamata: Varanidae) from the Early Oligocene of Egypt. Journal of Vertebrate Paleontology 28: 909–913.

[25] Wroe S (2002) A review of terrestrial mammalian and reptilian carnivore ecology in Australian fossil faunas, and factors influencing their diversity: the myth of reptilian domination and its broader ramifications. Australian Journal of Zoology 50: 1–24.

[26] Van Valen L (1973) A new evolutionary law. Evolutionary Theory 1: 1–30.

[27] Hocknull SA, Piper PJ, van den Bergh GD, Due RA, Morwood MJ, et al (2009) Dragon's Paradise Lost: palaeobiogeography, evolution and extinction of the largest-ever terrestrial lizards (Varanidae). Public Library of Science [PLoS] One 4: e7241 7241–7215. 10.1371/journal.pone.0007241 PMC2748693 19789642

[28] Soulinias N (2007) Samos Island, Part II: ancient hisory of the Samos fossils and the record of earthquakes. In: Lister G, Forster M, Ring U, editors. Inside the Aegean Metamorphic Core Complexes: Journal of the virtual explorer, electronic edition.

[29] Soulinias N (1981) The Turolian fauna from the island of Samos, Greece. Contributions to Vertebrate Evolution 6: 1–232.

[30] Solounias N (1981) Mammalian fossils of Samos and Pikermi, part 2. Resurrection of a classic Turolian fauna. Annals of the Carnegie Museum 50: 231–270.

[31] Rieppel O, Zaher H (2000) The braincases of mosasaurs and *Varanus*, and the relationships of snakes. Zoological Journal of the Linnean Society 129: 489–514.

[32] Rieppel O (1980) The phylogeny of anguinimorph lizards. Basel: Naturforschenden Gesellshaft. 86 p.

[33] Rieppel O (1983) A comparison of the skull of *Lanthanotus boreensis* (Reptilia: Varanoidea) with the skull of primitive snakes. Zeitschrift für zoologische Systematik und Evolutionsforschung 21: 142–153.

[34] Conrad JL (2004) Skull, mandible, and hyoid of *Shinisaurus crocodilurus* Ahl (Squamata, Anguimorpha). Zoological Journal of the Linnean Society 141: 399–434.

[35] Head JJ, Barrett PM, Rayfield EJ (2009) Neurocranial osteology and systematic relationships of *Varanus* (*Megalania*) *prisca* Owen, 1859 (Squamata: Varanidae). Zoological Journal of the Linnean Society 155: 445–457.

[36] Conrad JL, Norell MA (2006) High-resolution x-ray computed tomography of an Early Cretaceous gekkonomorph (Squamata) from Öösh (Övörkhangai; Mongolia). Historical Biology 18: 405–431.

[37] Bever GS, Bell CJ, Maisano JA (2005) The ossified braincase and cephalic osteoderms of *Shinisaurus crocodilurus* (Squamata, Shinisauridae). Palaeontologia Electronica 8: 1–36.

[38] Mertens R (1942) Die Familie der Warane (Varanidae). Zweiter Teil: Der Schädel. Abhandlungen der Senckenbergischen Naturforschenden Gesellschaft 465: 117–234.

[39] Evans SE (2008) The skull of lizards and tuatara. In: Gans C, Gaunt AS, Adler K, editors. Biology of the Reptilia, volume 20: Morphology H, the skull of Lepidosauria. Ithica, New York: Society for the Study of Amphibians and Reptiles. pp. 1–344.

[40] Bever GS, Bell CJ, Maisano JA (2005) *Shinisaurus crocodilurus*. Austin: Digital Morphology.

[41] Maisano JA (2001) A survey of state of ossification in neonatal squamates. Herpetological Monographs 15: 135–157.

[42] Bell GL, Polcyn MJ (2005) *Dallasaurus turneri*, a new primitive mosasauroid from the Middle Turonian of Texas and comments on the phylogeny of Mosasauridae (Squamata). Netherlands Journal of Geosciences 84: 177–194.

[43] Haber A, Polcyn MJ (2005) A new marine varanoid from the Cenomanian of the Middle East. Netherlands Journal of Geosciences 84: 247–255.

[44] Caldwell MW (2000) On the aquatic squamate *Dolichosaurus longicollis* Owen, 1850 (Cenomanian, Upper Cretaceous), and the evolution of elongate necks in squamates. Journal of Vertebrate Paleontology 20: 720–735.

[45] Lee MSY, Caldwell MW (2000) *Adriosaurus* and the affinities of mosasaurs, dolichosaurs, and snakes. Journal of Paleontology 74: 915–937.

[46] Polcyn MJ, Bell GL Jr (2005) *Russellosaurus coheni* n. gen., n. sp., a 92 million-year-old mosasaur from Texas (USA), and the definition of the parafamily Russellosaurina. Netherlands Journal of Geosciences 84: 321–333.

[47] Weithofer A (1888) Beiträge zur Kenntniss der Fauna von Pikermi bei Athen. Beiträge zur Paläontologie Oesterreich-Ungarns 6: 225–292.

[48] de Ferjérváry GJ (1918) Contributions to a monography on fossil Varanidae and Megalanidae. Annales Historico-Naturales Musei Nationalis Hungarici, pars zoologie 16: 341–467.

[49] Holmes RB, Murray AM, Attia YS, Simons EL, Chatrath P (2010) Oldest known *Varanus* (Squamata: Varanidae) from the Upper Eocene and Lower Oligocene of Egypt: support for an African origin of the genus. Palaeontology 53: 1099–1110.

[50] Ast JC (2001) Mitochondrial DNA evidence and evolution in Varanoidea (Squamata). Cladistics 17: 211–226. 10.1111/j.1096-0031.2001.tb00118.x 34911248

[51] Ast JC (2002) Evolution in Squamata (Reptilia) [Ph.D. dissertation]. Ann Arbor: University of Michigan. 276 p.

[52] Conrad JL, Norell MA (2008) The braincases of two glyptosaurines (Anguidae, Squamata) and anguid phylogeny. American Museum Novitates 3613: 1–24.

[53] Page RDM (2001) NDE: Nexus Data Editor for Windows. Glasgow: Page, R. D. M.

[54] Goloboff PA, Farris JS, Nixon KC (2003) T.N.T.: Tree Analysis Using New Technology. 1.0 ed. www.zmuc.dk/public/phylogeny: Goloboff, Farris, and Nixon.

[55] Maddison WP, Maddison DR (2006) Mesquite: A modular system for evolutionary analysis, version 1.11. 1.11 ed. http://mesquiteproject.org: Maddison, W. P. and Maddison, D. R.

[56] Burnell A, Collins S, Young BA (2012) Vertebral morphometrics in *Varanus* . Bulletin Societie Géologique de France 183: 151–158.

[57] Palci A, Caldwell MW (2007) Vestigial forelimbs and axial elongation in a 95 million-year-old non-snake squamate. Journal of Vertebrate Paleontology 27: 1–7.

[58] Rieppel O, Zaher H (2001) Re-building the bridge between mosasaurs and snakes. Neues Jahrbuch für Geologie und Paläontologie, Abhandlungen 221: 111–132.

[59] Townsend TM, Larson A, Louis E, Macey JR (2004) Molecular phylogenetics of Squamata: the position of snakes, amphisbaenians, and dibamids, and the root of the squamate tree. Systematic Biology 53: 735–757. 15545252 10.1080/10635150490522340

[60] Vidal N, Hedges SB (2005) The phylogeny of squamate reptiles (lizards, snakes, and amphisbaenians) inferred from nine nuclear protein-coding genes. Comptes rendus Biologies 328: 1000–1008. 16286089 10.1016/j.crvi.2005.10.001

[61] Vidal N, Hedges SB (2009) The molecular evolutionary tree of lizards, snakes, and amphisbaenians. Comptes Rendus Biologies 332: 129–139. 19281946 10.1016/j.crvi.2008.07.010

[62] Kearney M, Clark JM (2003) Problems due to missing data in phylogenetic analyses including fossils: a critical review. Journal of Vertebrate Paleontology 23: 263–274.

[63] Norell MA, Clark JM, Turner AH, Makovicky PJ, Barsbold R, et al (2006) A new dromaeosaurid theropod from Ukhaa Tolgod (Ömnögov, Mongolia). American Museum Novitates 3545: 1–51.

[64] Turner AH, Hwang SH, Norell MA (2007) A small derived theropod from Öösh, Early Cretaceous, Baykhangor Mongolia. American Museum Novitates 3557: 1–27.

[65] Falconer HP (1868) Paleontological Memoirs and Notes of the Late Hugh Falconer. London, UK: Robert Hardwicke. 465 p.

[66] King D, Fuller S, Baverstock P (1999) The biogeographic origins of varanid lizards. In: Horn H-G, Böhme W, editors. Mertensiella: advances in monitor research II. Rhine Brook: Deutcsche Gesellschaft für Herpetologie und Terrarienkunde. pp. 43–49.

[67] King M (1990) Chromosomal and immunogenetic data: a new perspective on the origin of Australia's reptiles. In: Olmo E, editor. Cytogenetics of amphibians and reptiles. Basel: Birkhauser. pp. 153–180.

[68] King M, King DR (1975) Chromosomal evolution in the lizard genus *Varanus* (Reptilia). Australian Journal of Biological Sciences 28: 89–108. 1164258

[69] Fuller S, Baverstock P, King D (1998) Biogeographic origins of goannas (Varanidae): a molecular perspective. Molecular Phylogenetics and Evolution 9: 294–307. 9562987 10.1006/mpev.1997.0476

[70] Conrad JL, Rieppel O, Grande L (2008) Re-assessment of varanid evolution based on new data from *Saniwa ensidens* Leidy, 1870 (Squamata, Reptilia). American Museum Novitates 3630: 1–15.

[71] Colli GR, Zamboni DS (1999) Ecology of the worm-lizard *Amphisbaena alba* in the Cerrado of Central Brazil. Copeia 1999: 733–742.

[72] Meiri S (2010) Length-weight allometries in lizards. Journal of Zoology 2010: 1–0.

[73] Zug GR, Vitt LJ, Caldwell JP (2001) Herpetology: an introductory biology of amphibians and reptiles. London, United Kingdom: Academic Press. 630 p.

[74] Schwartz A, Henderson RW (1991) Amphibians and Reptiles of the West Indies: descriptions, distributions, and natural history. Gainesville, FL: University Press of Florida. 714 p.

[75] Espinoza RE, Wiens JJ, Tracy CR (2004) Recurrent evolution in small, cold-climate lizards: Breakig the ecophysiological rules of reptilian herbivory. Proceedings of the National Academy of Sciences 101: 16819–16824. 10.1073/pnas.0401226101 PMC534712 15550549

[76] Pregill GK, Worthy TH (2003) A new iguanid lizard (Squamata, Iguanidae) from the Late Quaternary of Fiji, southwest Pacific. Herpetologica 59: 57–67.

[77] Lingham-Soliar T (1995) Anatomy and functional morphology of the largest marine reptile known, *Mosasaurus hoffmanni* (Mosasauridae, Reptilia) from the Upper Cretaceous, Upper Maastrichtian of The Netherlands. Philosophical Transactions of the Royal Society of London, Series B: Biological Sciences 347: 155–180.

[78] Bakker RT (1986) The dinosaur heresies. New York: William Morrow and Company, Inc. 481 p.

[79] Auffenberg W (1981) The behavioral ecology of the Komodo monitor. Gainesville: University Presses of Florida. 406 p.

[80] Pepin DJ (1999) The origin of monitor lizards based on a review of the fossil evidence. In: Horn H-G, Böhme W, editors. Mertensiella: advances in monitor research II. Rhine Brook: Deutcsche Gesellschaft für Herpetologie und Terrarienkunde. pp. 11–42.

[81] Pepin DJ (2001) Natural history of monitor (family Varanidae) with evidence from phylogeny, ecology, life history and morphology [Ph.D. dissertation]. Saint Louis: Washington University. 234 p.

[82] Flower BP, Kennett JP (1994) The middle Miocene climatic transition: East Antarctic ice sheet development, deep ocean circulation and global carbon cycling. Palaeogeography, Palaeoclimatology, Palaeoecology 108: 537–555.

[83] Douady CJ, Catzeflis F, Raman J, Springer MS, Stanhope MJ (2003) The Sahara as a vicariant agent, and the role of Miocene climatic events, in the diversification of the mammalian order Macroscelidea (elephant shrews). Proceedings of the National Academy of Sciences 100: 8325–8330. 10.1073/pnas.0832467100 PMC166228 12821774

[84] Case TJ (1978) A general explanation for insular body size trends in terrestrial vertebrates. Ecology 59: 1–18.

[85] Case TJ (1982) Ecology and evolution of the insular giant chuckwallas, *Sauromalus hispidus* and *Sauromalus varius*. In: Burghardt GM, Rand AS, editors. Iguanas of the world: Their behaviour, ecology and conservation. Park Ridge, New Jersey: Noyes Publications. pp. 184–212.

[86] Pianka ER (1994) Comparative ecology of *Varanus* in the Great Victoria Desert. Australian Journal of Ecology 19: 395–408.

[87] Head JJ, Bloch JI, Hastings AK, Bourque JR, Cadena EA, et al (2009) Giant boid snake from the Palaeocene neotropics reveals hotter past equatorial temperatures. Nature 457: 715–718. 19194448 10.1038/nature07671

[88] Murphy JC, Henderson RW (1997) Tales of Giant Snakes: A historical natural history of anacondas and pythons. Malabar, FL: Krieger.

[89] Goloboff PA, Farris JS, Nixon KC (2008) TNT, a free program for phylogenetic analysis. Cladistics 24: 774–786.

[90] Goloboff PA, Farris JS, Nixon KC (2010) T.N.T.: Tree Analysis Using New Technology, Willi Hennig Society Edition. 1.1 ed. www.zmuc.dk/public/phylogeny: Goloboff, Farris, and Nixon.

[91] Swofford DL (2001) PAUP*: Phylogenetic Analysis Using Parsimony. 4.0b10 ed. Washington, DC: Smithsonian Institution.

