# Supplementary material for: Earliest Example of a Giant Monitor Lizard (Varanus, Varanidae, Squamata)
Source: PLoS One. 2012 Aug 10;7(8):e41767. doi: 10.1371/journal.pone.0041767 (PMC3416840; doi:10.1371/journal.pone.0041767)
Supplement: DATASET S1 — MORPHOLOGICAL CHARACTER-BY-TAXON MATRIX. Here, we include the full morphological data matrix, including the character scorings for those characters described in earlier analyses. Note that some taxa were coded for only molecular characters (see text). Those taxa have a “?” for each coding but are included here for ease of the reader should she or he desire to reproduce our matrix. (DOC) [file pone.0041767.s001.doc]

**Earliest example of a giant monitor lizard (*Varanus*, Varanidae, Squamata)**

Jack L. Conrad^1^

Ana M. Balcarcel^2^

Carl M. Mehling^2^

^1^ Anatomy Department, New York College of Osteopathic Medicine, Old Westbury, NY

^2^ Department of Vertebrate Paleontology, American Museum of Natural History, New York, NY

**SUPPORTING INFORMATION**

**DATASET S1: Morphological Character-by-Taxon Matrix**

Here, we include the full morphological data matrix, including the character scorings for those characters described in earlier analyses. Note that some taxa were coded for only molecular characters (see text). Those taxa have a “?” for each coding but are included here for ease of the reader should she or he desire to reproduce our matrix.

*Shinisaurus crocodilurus* 1101000000 011000?000 01010001[01]1 100000000[01] 0101102000 0?0?010011 0111010000 0110000110 0011011001 1000100110 0010101101 ?010010000 00000021?1 [01]010011000 0010000011 2100000000 2000000000 01[01]1100011 0110100000 000?000101 0200001000 1010001101 20010[12]2?11 0011010000 0000000000 1100001010 0001000001 0000101000 21000021?1 0000100000 0100032110 0???0????? 00?0?00001 00?0100??? ?????????? ???8000000 10001110?0 ?00?01?0?1 100000?1?0 ?01?02[01]0?0 00[89]?600??? ???10?0?01 06?0?01000 ??0?0????1 00[45]010???? ?????????? ???????0?? ????00000? 0??

‘*Saniwa*’ *feisti* 10?????0?0 11??00??0? ???????01? ??000?0??? 10??001000 01?110200? ????1????1 011?0?0?00 0?0000011? ???????0?? 1????????? ?????????? ?????????? ?????????? ?????????? ?????????? ?????00??? ??????01?0 ?????????? ?????????? ??0?000??? 120?00?100 ???000???1 2?1?122??? ????010000 02??00???0 1???0??0?? ????00?00? ?00????000 ?1?????1?1 001?1?1??0 01???????? ?????????? ?????????? ?????????? ?????????? ???4?????? ??0??????? ?????????? ?????????? ????0????0 ?????????? ?????????? ?????????? ?????????? ?????????? ?????????? ?????????? ?????????? ???

*Necrosaurus cayluxi* ?????0?000 1?0??0??0? 0?30???01? ?0?????0?? ?????????? ????102000 10??1??001 0111110000 ??0000???? ???1?????? 1????????? ????2??1?? ???0?????? ?????????? ?????????? ?????????? ??????0??? 1??????10? 010???000? ?????????? ?????????? 1202001100 20??00???1 201?1????? 1?1??1??00 0211?????? ?????????? ???????0?? ?????????? ?????????? 0?0??0??00 01???????? ?????????? ?????????? ?????????? ?????????? ???4?????? ???????0?? ?????????? ???00????? ????0????? ?????????? ?????????? ?????????? ?????????? ?????????? ?????????? ?????????? ?????????? ???

*Necrosaurus eucarinatus* ?????00??1 ?????????? ??30???01? ?01??????? ?????????? ????0?200? 100????001 ?110010000 ??0000???? ?????????? ?????????? ?????????? ?????????? ?????????? ?????????? ?????????? ??????0??1 1??????00? 01?22??0?? ?????????? ?????????? 1202001100 ????00???? ?????????? ?????1???? ?????????? ?????????? ?????????? ?????????? ???????1?? 0?1?1????0 01???????? ?????????? ?????????? ?????????? ?????????? ???4?????? ???????0?? ?????????? ?????????? ????0????? ?????????? ?????????? ?????????? ?????????? ?????????? ?????????? ?????????? ?????????? ???

*Proplatynotia longirostrata* 1100?10011 1101001000 0030011110 1010000000 1000001101 010?002000 0?0?11?011 011001000? 000000??10 00100????? ?000?00110 1100100011 0010010??? ???????0?? ????0010?? ?????????? ?????00000 ??????0100 0112120001 0?11?1???? ?00??????? 1200001000 201000???? ?????????? ?????????? ?????????? ?????????? ?????????? ?????????? ?????????? ?????????? ?????????? ?????????? ?????????? ?????????? ?????????? ???8?0?0?0 ??001110?0 101001?0?? ??0??????? ?01?0310?? ?????????? ?????????? ?????????? ?????????? ?????????? ?????????? ?????????? ?????????? ???

*Paravaranus angustifrons* ?????10001 1?0?0??001 ?130???110 ?010000110 100?00110? 01??102100 0?0?000011 0111010?00 001000??10 02000????? ????1011?0 1100101011 0000000100 100?00?000 ?010?0?000 00100???1? ??????000? ??????01?? 01????001? 0????????? ?00?100??1 1200?00000 ???000???1 2????????? ?????????? ?????????? ?????????? ?????????? ?????????? ?????????? ?????????? ?????????? ?????????? ?????????? ?????????? ?????????? ???80?0??0 ??0??100?0 10??00???? ??000??1?? ????0????? ?????????? ?????????? ?????????? ?????????? ?????????? ?????????? ?????????? ?????????? ???

*Saniwides mongoliensis* 1101?10111 1201000000 013001?11? 10101?00?0 10[01]?001100 010100200? 100?110001 ?110010?00 0?0000??10 0?1102100? ????1?1110 110?0?1011 0001000000 000??021?0 ?01???1000 00100???11 ?100?0?00? ???????100 01120?0001 0011110001 000?100101 1202001100 201000???? ?????????? ?????????? ?????????? ?????????? ?????????? ?????????0 ?1?????1?? ?????????? ?????????? ?????????? ?????????? ?????????? ?????????? ???800???? ??0??????? 0?????0??? ???00????0 ?0??0??0?? ?????????? ?????????? ?????????? ?????????? ?????????? ?????????? ?????????? ?????????? ???

*Cherminotus longifrons* 111??1?000 110100000? 0?300??110 ?110001110 1?00001100 01??000000 10??0100?1 011?010?0? ?010000?1? 00100????? ????100110 11101011?1 0?0???0?00 0000??200? ??1?0??000 ??100????1 ?000???00? ?01??????? ?????????? ?11??1?001 ?????????? 1????????? ?????????? ?????????? ?????????? ?????????? ?????????? ?????????? ?????????? ?????????? ?????????? ?????????? ?????????? ?????????? ?????????? ?????????? ???80?0??? 1?00011??0 10??02?0?1 ???000???0 ?????????? ?????????? ?????????? ?????????? ?????????? ?????????? ?????????? ?????????? ?????????? ???

*Lanthanotus borneensis* 101?01?000 1101000001 012001?11? 1110001100 1010001100 0101001000 100?010001 011?013000 001?000110 001?1??011 1000100111 1110101111 0001010000 0000002001 1000011010 1010000011 2100000001 ?010000000 01120?0001 0101110001 000?100101 1202001100 2010001101 2012122?01 02[12]1010000 0211000000 11[01]1003020 0002000000 0001101000 210000?1?1 0000101001 0100034111 01?10001?1 1101?00012 0011?1?0?? 11???????? ???8000001 10010120?0 ?01002?0?1 000000?100 ?01?020000 00?671?210 00?0??0?00 00?0??0001 0?00100?01 21?01011?? ?????????? ?????????? ??[34]100010? 0??

*Ovoo gurvel* 211??1?000 1101001000 0130011110 0010001110 100000???? 110?002000 110?01?001 0????????? ????????10 00110?1??? 10?1101110 111020101? ?0?101???? ?????????? ?????????? ?????????? ?????????? ?????????? ?????????? ?????????? ?????????? 1202001100 101000???? ?????????? ?????????? ?????????? ?????????? ?????????? ?????????? ?????????? ?????????? ?????????? ?????????? ?????????? ?????????? ?????????? ???8?????0 2?0?0111?0 2?1002???? ??000????? ????0510?? ?????????? ?????????? ?????????? ?????????? ?????????? ?????????? ?????????? ?????????? ???

*Aiolosaurus oriens* 111??1001? 1201001000 01???1?110 ??100??0?0 1000?0???? ?1?1?0???? ???????0?1 ?????????? ??????01?0 ???????001 1000?????? ?1???????? ?????????? ?????????? ?????????? ?????????? ??00?00??? ?011000000 01?20?0000 0000?1??01 ?00?100??1 1202001000 ?0100????? ??1??????? ?????????? ?????????? ?????????? ?????????? ?????????? ?????????? ?????????? ?????????? ?????????? ?????????? ?????????? ?????????? ???8?0?0?? ??0?01?1?? ??100????1 ?????????? ??1??310?? ?????????? ?????????? ?????????? ?????????? ?????????? ?????????? ?????????? ?????????? ???

*Telmasaurus grangeri* ???0???000 ?????????? ?????????? ??100?0??0 10100?1100 01?1[01]?211? 10???10001 0110010000 01?0000110 002[01]001001 ?????????0 1100001011 000100?000 000??02001 ??1??11000 00100???11 ?100?????? ????????0? ?????????? ?????????? ?????????? 1?02??1?00 2???0????1 2?021?2??? ????010??0 ????00???? ????0????? ????0?00?? ???1100000 ?1???????? ?????????? ?????????? ?????????? ?????????? ?????????? ?????????? ???80????? ??0??????? ?????????? ???00????0 ?????????? 0????????? ?????????? ?????????? ?????????? ?????????? ?????????? ?????????? ?????????? ???

*Dolichosaurus longicollis* ?????????? ?????????? ?????????? ?????????? ?????????? ?????????? ?????????? ?????????? ?????????? ?????????? ?????????? ?????????? ?????????? ?????????? ?????????? ?10??????? ?????????? ???????10? 1??????0?1 11???1???? ?0???????? ?????????? ?????????1 2001222??? 03??01???? 01??000??? ?000???1?? 0?1?00002? ?????????? ?????????? ?????????? ?????????? ?????????? ?????????? ?????????? ?????????? ???4?????? ?????????? ?????????? ?????????? ?????????0 ?0???????? ?????????? ?????????? ?????????? ?????????? ?????????? ?????????? ?????????? ???

*Coniasaurus crassidens* ?????????? ?????????? ?????????? ?????????? ?????????? ?????????? ?????????? ?????????? ?????????? ?????????? ?????????? ?????????? ?????????? ?????????? ?????????? ?????????? ???????10? ?01000?100 11020?0011 1??101???? ?00??????? 1312?00000 1???00???? ?????????? ?????????? ?????????? ?????????? ?????????? ?????????? ?????????? ?????????? ?????????? ?????????? ?????????? ?????????? ?????????? ???4?????? ?????????? ?????????? ?????????? ??0?0????? ?????????? ?????????? ?????????? ?????????? ?????????? ?????????? ?????????? ?????????? ???

*Adriosaurus suessi* 110??1?000 1????????0 0030???110 ??000?00?? ????0????? ????00010? ????????11 011?010000 000??0011? 001??????? ????1????? ?????????? ?????????? ?????????? ?????????? ????????1? ?????0??0? ??????0??? ?????????? ?????????? ????101??? ?????????? ?????????? ????222??? 03??1?0?11 0212000??? ?00??????? ????0??00? 00012??100 ?0???????? ?????????? ?????????? ?????????? ?????????? ?????????? ?????????? ???40????? ??0??1???0 10???????? ?????????? ?????????? ?0???????? ?????????? ?????????? ?????????? ?????????? ?????????? ?????????? ?????????? ???

*Pontosaurus lesinensis* 2100?1?000 120?000000 1130?11110 ??001?0110 ????001100 010110010? ????0??1?1 021?1?0?00 ???0?00?10 0??00?1001 ?????????? ??????12?? ?????????? ???00????? ??????11?0 0???????1? ?011?0110? ?1??001000 0102??1011 111101??11 000?101?01 1202001000 ???000???1 2001?22??? 0311110011 0211000?0? ?10?10?0?? ????00200? ?00??????? ?????????? ?????????? ?????????? ?????????? ?????????? ?????????? ?????????? ???40??00? ??0??1???0 100???0??1 ?????????0 0?1?0????0 ?0??9????? ?????????? ?????????? ?????????? ?????????? ?????????? ?????????? ?????????? ???

*Aigialosaurus dalmaticus* 2100?1?000 ??1?000??? ???????110 ??101?01?0 1???011100 01011?200? ???????111 0110010?00 000000??10 0011021001 ?????????? ?????????? ??????0??0 ??0??????? ??1???1??? ????????11 ?01010?10? ?1????1100 01?2??1011 1?1101??01 000?100101 1202001000 1??000???1 2001222??? 0?11010?11 0201000??? ?????????? ????00?00? 1001101000 ?????????? ?????????? ?????????? ?????????? ?????????? ?????????? ?????????? ???4?????? ??0??????? ??????0??? ?????????0 ????0????0 0????????? ?????????? ?????????? ?????????? ?????????? ?????????? ?????????? ?????????? ???

*Saniwa ensidens* 1100?10001 110?000001 ?130?1?110 ?100000??0 1111001100 ?1?1002000 ????0??001 0110010?00 0100000110 00110?1001 10??101110 0100201111 0001010??? ??000020?? ??10???0?0 ??1?????1? ??00?0?001 1?????0000 01????00?? ?1?111??01 000?101101 1202001100 2??000???1 2012122?01 12110?0?00 0211001000 111?002??? 0?0200000? 1001??0000 00???0???? 0?0?0?0?0? 0????????? ?????????? ?????????? ?????????? ?????????? ???3000??0 1?0??111?0 101001?0?1 11100????? ????0??000 00?37????? ???11????? ?????????? ?????????? ?????????? ?????????? ?????????? ?????????? ???

*Varanus acanthurus* 1100010001 1100001000 0130011110 1110000100 1111001100 12?1002100 110?000011 0211010000 0000000110 0011021001 100?101110 0100101211 0001010000 0000002001 0010011010 1010000011 ?000000001 1010000000 01120?0011 0101110001 000?101101 1202001100 2010001101 2022022?01 1211010000 0211001000 1111002000 010200000? 1001200000 01??00?1?1 0000?????? 01000????? ?????????? ?????????? ?????????? ?????????? ???9000000 1100111100 201101?0?1 111??0?000 ?01?0?10?0 00??700??? ???0?00?11 12?0??0010 ??01101001 1000101010 01100????? 0100000022 ??0000000? 020

*Varanus baritji* ?????????? ?????????? ?????????? ?????????? ?????????? ?????????? ?????????? ?????????? ?????????? ?????????? ?????????? ?????????? ?????????? ?????????? ?????????? ?????????? ?????????? ?????????? ?????????? ?????????? ?????????? ?????????? ?????????? ?????????? ?????????? ?????????? ?????????? ?????????? ?????????? ?????????? ?????????? ?????????? ?????????? ?????????? ?????????? ?????????? ???9?????? ?1???????? ?????????? ?????????? ?????????? ?????1???? ??????1011 12?0??0??? ?????????? ?????????? ?????????? ?????????? ????00000? 020

*Varanus beccarii* 210001?000 120?001001 1130010110 1110000100 1111001100 02?1002000 110?000011 0211010?00 0000000110 0011011001 100?101110 0100101211 0001010000 0000002001 ?010011010 10100?0011 ?0000????? ?????????? ?????????? ?????????? ?????????? 1202001100 201000???1 2012022?01 1211010000 0211001000 111?002??? 0?0200000? 1??1100000 ???????1?1 0000????0? 01000????? 0????????? ?????????? ?????????? ?????????? ???80?0??0 1100?11110 201101?0?1 102??10010 ????0??0?0 00?171???? ??01100?00 00?1??0000 ??21101[01]01 10[01]0101010 112101[01]??1 011111000? 02[23]20000?? ???

*Varanus* cf. *bengalensis* ?????????? ???????100 ??1??????? ?????????? ?????????? ?????????? ?????????? ?????????? ?????????? ?????????? ?????????? ?????????? ?????????? ?????????? ?????????? ?????????? ?????????? ?????????? ?????????? ?????????? 151200???0 ???0?0???1 2??20?2??? ?????????? ?????????? ?????????? ?????????? ?????????? ?????????? ?????????? ?????????? ?????????? ?????????? ?????????? ?????????? ???7?????? ?????????? ??10?????? ?????????? ????0????? ?????????? ?????????? ?????????? ?????????? ?????????? ?????????? ?????????? ?????????? ???

*Varanus bengalensis* 310001?000 1200001001 0130011100 1010000100 1111001100 12?1002000 110?000011 0210010000 0[01]00000110 0010011001 100?101111 1100101211 0001010[01]00 0000002001 1000011010 1010000011 ?10000000? ?000000000 01020?0011 011111000? ?00?101101 1202001100 2010001101 2022022?00 1211010000 0211001000 1110002000 000200000? 1001100000 00??00?1?1 0000?????? 00000????? 0????????? ?????????? ?????????? ?????????? ???[78]000000 1100?10110 001[01]01?0?1 11[02]??10110 ?01?0?1010 00?17[01]0300 1111211100 01?0??0100 ??01101[23]11 10[23]0101011 [12]11001[12]1[12]1 0200111022 22[01]010010? 02?

*Varanus bivittatus* ?????????? ?????????? ?????????? ?????????? ?????????? ?????????? ?????????? ?????????? ?????????? ?????????? ?????????? ?????????? ?????????? ?????????? ?????????? ?????????? ?????????? ?????????? ?????????? ?????????? ?????????? ?????????? ?????????? ?????????? ?????????? ?????????? ?????????? ?????????? ?????????? ?????????? ?????????? ?????????? ?????????? ?????????? ?????????? ?????????? ???8?????? ?1???????? ?????????? ?????????? ?????????? ?????00??? ?????????? ???0?????? ?????????? ?????????? ?????????? ?????????? ????????0? 02?

*Varanus brevicauda* ?????????? ?????????? ?????????? ?????????? ?????????? ?????????? ?????????? ?????????? ?????????? ?????????? ?????????? ?????????? ?????????? ?????????? ?????????? ?????????? ?????????? ?????????? ?????????? ?????????? ?????????? ?????????? ?????????? ?????????? ?????????? ?????????? ?????????? ?????????? ?????????? ?????????? ?????????? ?????????? ?????????? ?????????? ?????????? ?????????? ???9?????? ?1???????? ?????????? ?????????? ?????????? ?0???1???? ??????0?01 12?0??0??? ?????????? ?????????? ?????????? ?????????? ????00000? 003

*Varanus cumingi* 2100010001 120?001001 1130011110 10100001?0 111100110? 12?1002000 110?000011 0111010?00 0000000110 0011021001 100?101110 0100101211 0001010100 0000002001 ??10001010 1??00???11 ?000?00??? ?00???0000 01?20?0??? ?11111?00? ?00?1001?? 1202001100 201000???? ?????????? ?????????? ?????????? ?????????? ?????????? ?????????? ?????????? ?????????? ?????????? ?????????? ?????????? ?????????? ?????????? ???8?????? ?1????1??? ?????????? ?????????? ?????????? ?0???00??? ??0???1111 14?0?01100 ??01101[01]01 10[01]0121010 [12]11101[01]1[01]1 0101010?02 ?2??000011 02?

*Varanus doreanus* 210001?000 1100001001 1130011110 1110000100 1111001100 11?1002100 110?000011 0111010000 001000???? ?????????? ?????????? ?????????? ?????????? ?????????? ?????????? ?????????? ?????????? ?????????? ?????????? ?????????? ?????????? ?????????? ?????????? ?????????? ?????????? ?????????? ?????????? ?????????? ?????????? ?????????? ?????????? ?????????? ?????????? ?????????? ?????????? ?????????? ???9?????? ?1?1?1???0 ?0???????1 ?????????? ??1??????? 00??7[01]0??? ??????1100 02?1??0200 ??21101[12]01 10[12]0121011 021201[01]1[01]1 01010201?2 ??[56]200000? 003

*Varanus dumerilii* 2100010001 1100001001 0120011100 1010000100 1111001100 12?1002000 110?000011 0110010000 0011010110 0021031001 100?101111 1100101211 0001010100 0000002000 [01]000011010 1010000011 ?00000000? ?000000000 01120?0011 011111000? ?00?101101 1202001100 201000???1 2022022?01 1211010000 0211001000 1111002000 010200000? 1001200000 00??00?1?1 0000?????? 01000????? 0????????? ?????????? ?????????? ?????????? ???8000000 ?100112000 ?00?02?0?1 ?02??0?1?0 ?01?0??0?0 00??700??? ??0??10?11 16?0?01000 ??01111001 10[01]010?010 ?11??????? ???????022 ??[01]0100111 022

*Varanus eremius* 110001?000 1200001000 0130011100 10100001?0 111100110? 12?1002000 110?000011 0211010000 0000020110 0011021001 100?101110 0100101211 0001010?00 0000002?0? ?0?0011010 1010000011 ?100000001 1010000000 01120?0001 0111110001 000?100101 1202001100 201000???1 20?2022??? ?2?10100?0 021100?000 11??002000 0??200000? 100???0000 ??0000?1?1 0000?0?00? 00000????? 0????????? ?????????? ?????????? ?????????? ???90000?0 ?10001?100 201100?0?1 112????0?0 ?01?0??0?0 00??71???? ??????1101 05?0000??? ?????????? ?????????? ?????????? ?????????? ????00110? 103

*Varanus flavescens* 210001?000 1200000001 0130010110 1010000100 11110011[01]0 11?1002000 110?000011 0211010000 0000000110 0011021001 100?101110 1100101211 0001010000 0000002001 1000001010 10100???11 ?100000001 1010000000 01120?0011 0111110001 ?00?100101 1202001100 201000???1 2012022?01 1211010000 0211001000 ?11?00?000 ????00000? 1001100000 ?1??00?1?1 0000?????? 00000????? 0????????? ?????????? ?????????? ?????????? ???70000?0 1100011110 201001?0?1 111??10?00 ?01?0??0?0 00?1700300 11????0?01 0300[01]01010 ??01111[23]01 00[23]0101000 [12]33001[23]1[23]1 01101112?? 220010010? 00?

*Varanus giganteus* ?????????? ?????????? ?????????? ?????????? ?????????? ?????????? ?????????? ?????????? ?????????? ?????????? ?????????? ?????????? ?????????? ?????????? ?????????? ?????????? ?????????? ?????????? ?????????? ?????????? ?????????? ?????????? ?????????? ?????????? ?????????? ?????????? ?????????? ?????????? ?????????? ?????????? ?????????? ??0??????? ?????????? ?????????? ?????????? ?????????? ???9?????? ?????????? ?????????? ?????????? ?????????? ?0???????? ??????1100 0300010100 ??01101[12]01 00[23]011?011 211001[23]1[23]0 0000220222 ??0000000? 011

*Varanus gilleni* 1100010001 120000100? ??30011100 10100001?0 111100110? 12?1002000 110?000011 0?11010000 0000020110 0011021001 100?101110 1100101211 0001010?00 0000002?0? ?0?00?1010 1010000011 ?100000001 1010000000 01120?0001 0111110001 000?100101 1202001100 201000???1 20?2022??? ?2?10100?0 021100?000 11??002000 0??200000? 100???0000 ??0000?1?1 0000?0?00? 00000????? 0????????? ?????????? ?????????? ?????????? ???90000?0 ?10001?100 10110??0?1 112????0?0 ?01?0??0?0 00??71???? ??0???0?11 05?0010010 ??01101[01]00 00[12]0101110 00?011[12]1[12]0 0100000??? ??0000000? 013

*Varanus glebopalma* ?????????? ?????????? ?????????? ?????????? ?????????? ?????????? ?????????? ?????????? ?????????? ?????????? ?????????? ?????????? ?????????? ?????????? ?????????? ?????????? ?????????? ?????????? ?????????? ?????????? ?????????? ?????????? ?????????? ?????????? ?????????? ?????????? ?????????? ?????????? ?????????? ?????????? ?????????? ??0??????? ?????????? ?????????? ?????????? ?????????? ?????????? ?????????? ?????????? ?????????? ?????????? ?0???????? ??????1111 1310100??? ?????????? ?????????? ???001[34]1[34]1 0100010??? ?0??00000? 001

*Varanus gouldii* 3100010001 1200001001 0130011110 1010000100 111100110? 12?1002100 110?000011 0211110000 0100000110 0011021001 100?101110 0100101211 0001010000 0000002001 1000011010 1010000011 ?100000001 1010000000 01120?0011 0111110001 000?100101 1202001100 201000???1 2022022?01 1211010000 0211001000 1110002000 010200000? 1000200000 010000?1?1 0000?0?00? 01000????? 0????????? ?????????? ??????2??? ?????????? ???9000000 ?10[01]011100 201001?0?1 1?2??10000 ?01?0?10?0 00??700??? ??01101111 1300000000 ??01101[34]?1 00[34]0111001 [12]11001[12]1[12]0 0100000?22 ??[01]000110? 021

*Varanus* cf. *marathonensis* (skull) 10??1?00? 1????0???? ?????????? ???0?????? ?????????? ?????????? ?????????? ?????????? ?????????? ?????????? 1????????? ?????????? ?????????? ???????0?? ?????????? 1????????? ?????????? ?????????? ?????????? ?????????? ?????????? 120?00??00 ???0?????? ?????????? ?????????? ?????????? ?????????? ?????????? ?????????? ?????????? ?????????? ?????????? ?????????? ?????????? ?????????? ?????????? ???4??0??? ??0??1???? ??10?????? 1????0??1? ????0??0?? ?????????? ??0??????? ?????????? ?????????? ?????????? ?????????? ?????????? ?????????? ???

*Varanus griseus* 2100010001 1200001001 0130010100 10100001?0 1111001100 11?1002000 110?000011 0111010000 0100000110 0011021001 100?101110 1100101211 0001010100 0000002001 ?0100?1010 1010000011 2000000001 1010000000 01120?0011 010111?001 000?100101 1202001100 2010001101 20?2022?01 1211010000 0211001000 1110002000 0?0200000? 1001100000 000000?1?1 000010100? 00000?4111 010101???2 1101?00112 1?11112??? ?????????? ???60?0??0 ?201011100 201000?0?1 11???0?110 ?01?0??0?0 00??700??? ???0?11011 1[36]00000100 ??01101[12]01 10[12]0101011 041101[23]1[23]1 0000210??2 ?20010000? 022

*Varanus indicus* Australia ?????????? ?????????? ?????????? ?????????? ?????????? ?????????? ?????????? ?????????? ?????????? ?????????? ?????????? ?????????? ?????????? ?????????? ?????????? ?????????? ?????????? ?????????? ?????????? ?????????? ?????????? ?????????? ?????????? ?????????? ?????????? ?????????? ?????????? ?????????? ?????????? ?????????? ?????????? ??0??????? ?????????? ?????????? ?????????? ?????????? ???8?????? ?????????? ?????????? ?????????? ?????????? ?0???????? ?????????? ?????????? ?????????? ?????????? ?????????? ?????????? ?????????? ???

*Varanus indicus* Flores ?????????? ?????????? ?????????? ?????????? ?????????? ?????????? ?????????? ?????????? ?????????? ?????????? ?????????? ?????????? ?????????? ?????????? ?????????? ?????????? ?????????? ?????????? ?????????? ?????????? ?????????? ?????????? ?????????? ?????????? ?????????? ?????????? ?????????? ?????????? ?????????? ?????????? ?????????? ??0??????? ?????????? ?????????? ?????????? ?????????? ???8?????? ?????????? ?????????? ?????????? ?????????? ?0???????? ?????????? ?????????? ?????????? ?????????? ?????????? ?????????? ?????????? ???

*Varanus indicus* Solomon Islands 2100010001 11[01]000100[01] 0130011110 1[01]10000100 111100110? 12?1002100 110?000011 0211010000 0010000110 0011021001 100?101110 1100101211 0001010000 000000200? 10000?1010 1010000011 ?100000001 10[01]0000000 01020?0011 0111110001 000?100101 1202001100 201000???1 2012022?01 1211010000 021100?000 1111002000 000200000? 1001100000 000000?1?1 0000?0?00? 00000????? 0????????? ?????????? ??????2??? ?????????? ???8000010 110001[01]100 201001?0?1 100??[01]0100 ?01?0??0?0 00?1710300 110111???? ???????200 ??21101[01]01 00[01]012?020 012??????? ???????22? ??[56]2????0? 00?

*Varanus jobiensis* ?????????? ?????????? ?????????? ?????????? ?????????? ?????????? ?????????? ?????????? ?????????? ?????????? ?????????? ?????????? ?????????? ?????????? ?????????? ?????????? ?????????? ?????????? ?????????? ?????????? ?????????? ?????????? ?????????? ?????????? ?????????? ?????????? ?????????? ?????????? ?????????? ?????????? ?????????? ??0??????? ?????????? ?????????? ?????????? ?????????? ?????????? ?????????? ?????????? ?????????? ?????????? ?0???????? ??????0?00 01?0??0000 ??21101[01]01 10[01]0121010 112101?1?? 0000220?2? ??[78]200000? 013

*Varanus keithhornei* ?????????? ?????????? ?????????? ?????????? ?????????? ?????????? ?????????? ?????????? ?????????? ?????????? ?????????? ?????????? ?????????? ?????????? ?????????? ?????????? ?????????? ?????????? ?????????? ?????????? ?????????? ?????????? ?????????? ?????????? ?????????? ?????????? ?????????? ?????????? ?????????? ?????????? ?????????? ??0??????? ?????????? ?????????? ?????????? ?????????? ?????????? ?????????? ?????????? ?????????? ?????????? ?????????? ?????????? ?????????? ?????????? ?????????? ?????????? ???????0?? ????????0? 01?

*Varanus kingorum* ?????????? ?????????? ?????????? ?????????? ?????????? ?????????? ?????????? ?????????? ?????????? ?????????? ?????????? ?????????? ?????????? ?????????? ?????????? ?????????? ?????????? ?????????? ?????????? ?????????? ?????????? ?????????? ?????????? ?????????? ?????????? ?????????? ?????????? ?????????? ?????????? ?????????? ?????????? ??0??????? ?????????? ?????????? ?????????? ?????????? ?????????? ?1???????? ?????????? ?????????? ?????????? ?0???????? ??????0?00 07?0??0??? ?????????? ?????????? ?????????? ?????????? ????00000? 003

*Varanus komodoensis* 3110010001 1200001001 0130011110 1110000100 1111001100 1[12]?1002000 110?000011 0111010000 0100000110 0011021001 100?101110 0100101211 0001010000 0000002001 1000001010 1010000011 2100000001 1000000000 01120?0001 0111110001 000?100101 1202001100 2010001101 2022022?01 1211010000 021100100? ?11?002000 0???00000? 1001100000 ?0??00?1?1 0000?0?003 00000????? 0????????? ?????????? ?????????? ?????????? ???8000??0 ?000011100 201002?0?1 1?1??0?110 ?01?0?1000 00?0700??? ??01101111 10?0??0000 ??01100?01 00[23]0111001 [12]11201[12]1[12]0 0000000122 ???000000? 003

*Varanus marmoratus* 2100010001 1200001001 0130011110 10100001?0 111100110? 12?1002000 110?000011 0111010000 0110000110 0011021001 100?101110 1100101211 0001010000 000000200? ?010001010 10100?0011 ?00000000? ?000000000 01120?00?? ?111110001 000?100101 1202001100 201000???1 20?2022??? ?2?10100?0 021100?000 11??002000 0??200000? 100???0000 ??0000?1?1 0000?0?000 00000????? 0????????? ?????????? ?????????? ?????????? ???800?0?0 ?100011100 101001?0?1 112??10010 ?01?0??000 00??700??? ??0???1011 14?0111??? ?????????? ?????????? ?????????? ?????????? ????000011 02?

*Varanus melinus* ?????????? ?????????? ?????????? ?????????? ?????????? ?????????? ?????????? ?????????? ?????????? ?????????? ?????????? ?????????? ?????????? ?????????? ?????????? ?????????? ?????????? ?????????? ?????????? ?????????? ?????????? ?????????? ?????????? ?????????? ?????????? ?????????? ?????????? ?????????? ?????????? ?????????? ?????????? ??0??????? ?????????? ?????????? ?????????? ?????????? ???8?????? ?????????? ?????????? ?????????? ?????????? ?0???1???? ??????1001 1310??0??? ?????????? ?????????? ?????????? ?????????? ????00000? 00?

*Varanus mertensi* ?????????? ?????????? ?????????? ?????????? ?????????? ?????????? ?????????? ?????????? ?????????? ?????????? ?????????? ?????????? ?????????? ?????????? ?????????? ?????????? ?????????? ?????????? ?????????? ?????????? ?????????? ?????????? ?????????? ?????????? ?????????? ?????????? ?????????? ?????????? ?????????? ?????????? ?????????? ??0??????? ?????????? ?????????? ?????????? ?????????? ???9?????? ?????????? ?????????? ?????????? ?????????? ?0???1???? ??????1001 10?0??0000 ??01101[12]00 10[23]0111000 111011[23]1[23]1 0000110022 22[12]000000? 003

*Varanus mitchelli* ?????????? ?????????? ?????????? ?????????? ?????????? ?????????? ?????????? ?????????? ?????????? ?????????? ?????????? ?????????? ?????????? ?????????? ?????????? ?????????? ?????????? ?????????? ?????????? ?????????? ?????????? ?????????? ?????????? ?????????? ?????????? ?????????? ?????????? ?????????? ?????????? ?????????? ?????????? ??0??????? ?????????? ?????????? ?????????? ?????????? ???9?????? ?????????? ?????????? ?????????? ?????????? ?0???1???? ??????1011 11?0??0010 ??01101000 00[01]0101000 000??????? ?????????? ??0000000? 003

*Varanus nebulosus* 2????????? ?????????? ?????????? ?????????? ?????????? ?????????? ?????????? ?????????? ?????????? ?????????? ?????????? ?????????? ?????????? ?????????? ?????????? ?????????? ?????????? ?????????? ?????????? ?????????? ?????????? ?????????? ?????????? ?????????? ?????????? ?????????? ?????????? ?????????? ?????????? ?????????? ?????????? ??0??????? ?????????? ?????????? ?????????? ?????????? ???[89]?????? ?1???????? ?????????? ?????????? ?????????? ?0???1???? ??????1111 11?0??0000 ??01101[12]00 10[12]0101020 111??????? ????????22 ??[12]010000? ???

*Varanus niloticus* 21[01]0010001 1200001001 1130010110 1010000100 111100110? 11?1002000 110?000011 0211?10000 0100000110 0011021001 100?101110 1100101211 0001010100 0000002001 1010001010 1010000011 2000000001 1010000000 01120?0011 0111110001 000?100101 1512001100 2010001101 2012022?01 1211010000 0211001000 1110002000 000200000? 1001100000 010000?1?1 0000?0?00? 00000????? 0????????? ?????????? ?????????? ?????????? ???5000010 ?1010111[02]0 20??12?1?1 11???10110 ?01?0?1000 00??700300 1111011111 1300000010 ??00100?01 10?0101111 212000?0?1 011111021? 02[34]200010? 01?

*Varanus rudicollis* 3100010001 1100001001 0130011110 1010000100 1111001100 12?1002000 110?000011 0211010000 0010020110 0011021001 100?101110 1100101211 0001010000 0000002001 00100?1010 1010000011 2000000001 1010000000 01120?0011 0111110001 000?100101 1202001100 2010001101 2022022?11 1211010000 0211001000 1111002000 001200000? 1001110000 01?000?1?1 0000?0?000 01000????? 0????????? ?????????? ?????????? ?????????? ???[89]000010 ?10??1???0 ?0???????1 1????[01]0100 ?01?0??0?0 00??700??? ??01001101 1300?00100 ??01101[12]01 00[12]0101110 211001[01]1[01]1 0101120?02 0??0100112 003

*Varanus albigularis* 2100010001 1200001001 0130010110 1010000100 1111001100 11?1002100 110?000011 0211010000 0100000110 0011021001 100?101111 1100101211 0001010100 0000002001 1010001010 1010000011 2100000001 1010000000 01020?0011 0111110001 000?101101 1512001100 2010001101 2012122?01 1211010000 0211001000 1110002000 000200000? 1001100000 000000?1?1 0000?0?00? 00000????? 0????????? ?????????? ?????????? ?????????? ???5000010 220[01]010120 201012?1?1 002??11100 ?01?031010 00?[12]700??? ??21010?01 0300001100 ??01101[01]01 10[01]0101110 [12]11101[01]1[01]1 0101100[12]00 0?[12]000010? 02?

*Varanus exanthematicus* 2100010001 1200000001 1130010110 1010000100 1111001100 11?1002000 110?000011 0211010000 00[01]0000110 0011021001 100?101111 1100101211 0001010100 0000002001 1010001010 1010000011 2000000001 1010000000 01120?0011 0111110001 000?100101 1512001100 2010001101 2022122?02 1211010000 0211001000 1110002000 0?0200000? 1001100000 000000?1?1 0000?0?00? 00000????? 0????????? ?????????? ?????????? ?????????? ???5000010 ?201111120 201112?0?1 012??11100 ?01?03?0?0 00??700??? ??01010?01 0300001200 ??01101[12]01 10[12]0101011 211001[01]1[01]1 0110010200 ?2[01]200010? 003

*Varanus ornatus* 2100010001 1200001001 1130011110 1010000100 111100110? 11?1002000 110?000011 0211110?00 0100000110 0011021001 100?101110 0100101211 0001010100 0000002001 ?010011010 10100?0011 ?00000000? ?000000000 01?20?00?0 011111000? ?00?100101 1512001100 201000???? ?????????? ?????????? ?????????? ?????????? ?????????? ?????????? ?????????? ?????????? ??0??????? 0????????? ?????????? ?????????? ?????????? ???60000?0 1200011100 211012?1?1 ??2??10110 ?01?0310?? ?0???01??? ??01111111 1310001100 ??01101001 1000101111 1121110101 011111000? 12[45]000010? 01?

*Varanus rusingensis* ?????????? ?????????? ?????1?11? ?01??????? ?????????? ?????????? ?????????? 0210010000 ?10000???? ?????????? ?????????? ?????????? ?????????? ?????????? ?????????? ?????????? ?????00?0? ??????0000 01?20?0??? ????????01 000?100101 1502001100 ???000???1 20120?2??? 1???010000 021?00???? ?11?00???? ????0000?? ???1??0000 ?1???????? ?????????? ?????????? ?????????? ?????????? ?????????? ?????????? ???5?????? ?????????? ???????1?? ?????????? ??1?0????0 00???????? ?????????? ?????????? ?????????? ?????????? ?????????? ?????????? ?????????? ???

*Varanus olivaceus* 2100010001 1100001001 0130011110 1010000100 111100010? 11?1001000 110?000011 0211110000 0100000110 0011021001 100?101110 1100101211 0002010100 000000200? ?010011010 1010000011 ?00000000? ?010000000 01120?0011 0111110001 000?101101 1512001100 201000???1 20?2022??? ?2?10100?0 021100?000 11??002000 0??200000? 100???0000 ??0000?1?1 0000?0?00? 00000????? 0????????? ?????????? ?????????? ?????????? ???80?00?0 ?1000101[02]0 201012?0?1 102??101?0 ?01?0?10?0 00??700??? ??11001111 06?1?01000 ??01101[01]01 10[01]0101011 110101[01]1[01]0 010111000? 02[01]010010? 013

*Varanus hooijeri* ?1?????0?? ?????????? ?????1??1? ?????????? ?????????? ?????????? ?????????1 021?110000 000000???? ?????????? ?????????? ?????????? ?????????? ??0??02??? 1????????? ?????????? ??????0??? ???????00? 01???????? ?????????? ?????????? 1512001100 ???000???? ?????????? ?????????? ?????????? ?????????? ?????????? ?????????? ?????????? ?????????? ?????????? ?????????? ?????????? ?????????? ?????????? ???8?????? ?????????? ??10?????? ?????????? ?????????? ?????????? ??0??????? ?????????? ?????????? ?????????? ?????????? ?????????? ?????????? ???

*Varanus panoptes horni* ?????????? ?????????? ?????????? ?????????? ?????????? ?????????? ?????????? ?????????? ?????????? ?????????? ?????????? ?????????? ?????????? ?????????? ?????????? ?????????? ?????????? ?????????? ?????????? ?????????? ?????????? ?????????? ?????????? ?????????? ?????????? ?????????? ?????????? ?????????? ?????????? ?????????? ?????????? ??0??????? 0????????? ?????????? ?????????? ?????????? ???9?????? ?1???????? ?????????? ?????????? ?????????? ?0???1???? ?????????? ?????????? ?????????? ?????????? ?????????? ???????2?? ????????0? 011

*Varanus panoptes panoptes* ?????????? ?????????? ?????????? ?????????? ?????????? ?????????? ?????????? ?????????? ?????????? ?????????? ?????????? ?????????? ?????????? ?????????? ?????????? ?????????? ?????????? ?????????? ?????????? ?????????? ?????????? ?????????? ?????????? ?????????? ?????????? ?????????? ?????????? ?????????? ?????????? ?????????? ?????????? ??0??????? 0????????? ?????????? ?????????? ?????????? ???9?????? ?1???????? ?????????? ?????????? ?????????? ?0???1???? ??????1011 1300000??? ?????????? ?????????? ?????????? ???????2?? ????00000? 011

*Varanus pilbarensis* ?????????? ?????????? ?????????? ?????????? ?????????? ?????????? ?????????? ?????????? ?????????? ?????????? ?????????? ?????????? ?????????? ?????????? ?????????? ?????????? ?????????? ?????????? ?????????? ?????????? ?????????? ?????????? ?????????? ?????????? ?????????? ?????????? ?????????? ?????????? ?????????? ?????????? ?????????? ??0??????? 0????????? ?????????? ?????????? ?????????? ???9?????? ?1???????? ?????????? ?????????? ?????????? ?0???00??? ??????1011 1300100??? ?????????? ?????????? ?????????? ?????????? ????00110? 003

*Varanus prasinus* 2100010001 1100001000 1130011110 11100001?0 111100110? 12?1002000 110?000011 0111010000 0000000110 0011021001 100?101110 0100101211 0001010000 0000002001 0010011010 1010000011 ?100000001 1010000000 01120?00?? 011111?00? ?00?100101 1202001100 2010001??1 201202??00 1????????? ?????????0 111000???? ????00000? 10011000?? ?1??00?1?1 0000?????? 00000????? 0????????? ?????????? ?????????? ?????????? ???9000010 1100011?10 201101?0?1 112??10010 ?01?????00 00??700??? ??????1011 16?1?01?01 1?000???01 00?0101010 012??????? ???????02? ??[23]202000? 013

*Varanus kordensis* 2100010001 1100001000 1130011110 11100001?0 111100110? 12?1002000 110?000011 0111010000 0000000110 0011011001 100?101110 0100101211 0001010000 000000200? ?0100?1010 10100???11 ?100000001 1010??0000 01?20?00?? 0111110001 ?00?100101 1202001100 2010001??? ?????????? ?????????? ?????????? ?????????? ?????????? ?????????? ????00?1?1 0000?????? 01000????? 0????????? ?????????? ?????????? ?????????? ???90000?0 ?100011110 201101?0?1 112??10??0 ?01?0??0?? 00??700??? ??0???0?01 0310001??? ?????????? ?????????? ?????????? ?????????? ????00000? 00?

*Varanus primordius* ?????????? ?????????? ?????????? ?????????? ?????????? ?????????? ?????????? ?????????? ?????????? ?????????? ?????????? ?????????? ?????????? ?????????? ?????????? ?????????? ?????????? ?????????? ?????????? ?????????? ?????????? ?????????? ?????????? ?????????? ?????????? ?????????? ?????????? ?????????? ?????????? ?????????? ?????????? ??0??????? ?????????? ?????????? ?????????? ?????????? ???9?????? ?1???????? ?????????? ?????????? ?????????? ?0???1???? ??????1011 17?0000010 ??010???01 00[12]0101000 000??????? ?????????? ??0200000? 003

*Varanus priscus* ?11???00?1 ?????01??? ??3001?11? 1?????1??? ?????????? ????002120 110??00001 0111110000 ???0?0011? 0????????? ?????????? ???0?????? ??????010? 000?00?001 100000??10 10100????? ??????0??1 ???????00? 01???????? ?????????? ?????????? 1202001100 2??000???1 20220?2??? 1???010?00 021100???? ???????000 ????00?0?? ???1??0000 ?????????? 000??0???3 0????????? ?????????? ?????????? ?????????? ?????????? ???90?0??? ?????????0 ?01??????? ???????1?? ????0????0 0????????? ??111????? ?????????? ?????????? ?????????? ?????????? ?????????? ?????????? ???

*Varanus rosenbergi* ?????????? ?????????? ?????????? ?????????? ?????????? ?????????? ?????????? ?????????? ?????????? ?????????? ?????????? ?????????? ?????????? ?????????? ?????????? ?????????? ?????????? ?????????? ?????????? ?????????? ?????????? ?????????? ?????????? ?????????? ?????????? ?????????? ?????????? ?????????? ?????????? ?????????? ?????????? ??0??????? ?????????? ?????????? ?????????? ?????????? ???9?????? ?1???????? ?????????? ?????????? ?????????? ?0???00??? ??????1111 16?0???110 ??01101[01]01 10[01]0101011 011011[01]1[01]1 1?????0?00 ??[34]000000? 011

*Varanus salvadorii* 3110010001 1200001000 0130011110 1110000100 1111001100 12?10021[01]0 110?000001 0111010000 0[01]00000110 0011011001 100?101110 0100101211 0001010000 0000002001 0000011010 1010000011 2100000001 1000000000 01120?0001 0111110001 000?101101 1202001100 2010001101 20220???00 1??1??0??? ?????????? ?????????? ????00?0?? ???????000 ????00?1?1 0000?0?00? 00000????? 0????????? ?????????? ?????????? ?????????? ???90000?0 ?000110100 201101?0?1 ?12??10010 ?01?0310?0 00??700??? ??01001001 1310011000 ??01101[23]01 00[23]0101000 011111[56]1[56]0 ?000111021 00?000010? 002

*Varanus salvator* 2100010001 1200001001 1130011110 1010000100 111100110? 12?1002000 110?000011 0111010000 0110000110 0011021001 100?101110 1100101211 0001010000 0000002001 1000001010 10100???11 2000000001 1000000000 01120?0001 0111110001 000?100101 1202001100 201000???1 2022022?01 1211010000 0211001000 1110002000 010200000? 100???0000 010000?1?1 0000?0?00? 00000????? 0????????? ?????????? ??????2??? ?????????? ???80000?0 ?100011100 101001?0?1 112??10010 ?01?021000 00??700??? ??01101011 1310111100 ??01101[12]01 00[12]010??10 000??????? ???????2?? ??[12]100000? 02?

*Varanus scalaris* ?????????? ?????????? ?????????? ?????????? ?????????? ?????????? ?????????? ?????????? ?????????? ?????????? ?????????? ?????????? ?????????? ?????????? ?????????? ?????????? ?????????? ?????????? ?????????? ?????????? ?????????? ?????????? ?????????? ?????????? ?????????? ?????????? ?????????? ?????????? ?????????? ?????????? ?????????? ??0??????? ?????????? ?????????? ?????????? ?????????? ???9?????? ?1???????? ?????????? ?????????? ?????????? ?0???00??? ??????1101 1310000??? ?????????? ?????????? ?????????? ?????????? ????00100? 012

*Varanus semiremex* 2110010001 1100001000 0130011110 10100001?0 111100110? 12?1002000 110?000011 0111010000 0000000110 0011021001 100?101110 0100101211 0001010?00 0000002?0? ?0?00?1010 1010000011 ?100000001 1010000000 01120?0001 0111110001 000?100101 1202001100 201000???1 20?2022??? ?2?10100?0 021100?000 11??002000 0??200000? 100???0000 ??0000?1?1 0000?0?00? 01000????? 0????????? ?????????? ?????????? ?????????? ???90000?0 ?100?101?0 2011?????1 112????0?0 ?01?0??0?0 00??71???? ?????11?01 0??0???110 ??01101[01]01 10[01]0101000 000??????? ?????????? ??0000000? 003

*Varanus spenceri* ?????????? ?????????? ?????????? ?????????? ?????????? ?????????? ?????????? ?????????? ?????????? ?????????? ?????????? ?????????? ?????????? ?????????? ?????????? ?????????? ?????????? ?????????? ?????????? ?????????? ?????????? ?????????? ?????????? ?????????? ?????????? ?????????? ?????????? ?????????? ?????????? ?????????? ?????????? ??0??????? ?????????? ?????????? ?????????? ?????????? ???9?????? ?1???????? ?????????? ?????????? ?????????? ?0???00??? ??????1011 04?0?01100 ??01101001 1000101011 0110010100 1??????222 ??[34]100010? 013

*Varanus storri* ?????????? ?????????? ?????????? ?????????? ?????????? ?????????? ?????????? ?????????? ?????????? ?????????? ?????????? ?????????? ?????????? ?????????? ?????????? ?????????? ?????????? ?????????? ?????????? ?????????? ?????????? ?????????? ?????????? ?????????? ?????????? ?????????? ?????????? ?????????? ?????????? ?????????? ?????????? ??0??????? ?????????? ?????????? ?????????? ?????????? ???9?????? ?1???????? ?????????? ?????????? ?????????? ?0???1???? ??????1111 1310??0??? ?????????? ?????????? ?????????? ?????????? ????00000? 000

*Varanus timorensis* ?????????? ?????????? ?????????? ?????????? ?????????? ?????????? ?????????? ?????????? ?????????? ?????????? ?????????? ?????????? ?????????? ?????????? ?????????? ?????????? ?????????? ?????????? ?????????? ?????????? ?????????? ?????????? ?????????? ?????????? ?????????? ?????????? ?????????? ?????????? ?????????? ?????????? ?????????? ??0??????? ?????????? ?????????? ?????????? ?????????? ???9?????? ?1???????? ?????????? ?????????? ?????????? ?0???1???? ??011?1011 1510100110 ??01111[12]01 00[12]0101100 000??????? ?????????? ???000000? 003

*Varanus togianus* ?????????? ?????????? ?????????? ?????????? ?????????? ?????????? ?????????? ?????????? ?????????? ?????????? ?????????? ?????????? ?????????? ?????????? ?????????? ?????????? ?????????? ?????????? ?????????? ?????????? ?????????? ?????????? ?????????? ?????????? ?????????? ?????????? ?????????? ?????????? ?????????? ?????????? ?????????? ??0??????? ?????????? ?????????? ?????????? ?????????? ???8?????? ?1???????? ?????????? ?????????? ?????????? ?0???1???? ??????1100 01?0??0??? ?????????? ?????????? ?????????? ?????????? ????00000? 0??

*Varanus tristis* 2100010001 1110001000 1130011110 10100001?0 111100?10? 12?1002000 110?000011 0111010000 0000000110 0011011001 100?101110 0100101211 0001010?00 0000002?0? ?0?00?1010 1010000011 ?100000001 1010000000 01120?0011 0111110001 000?100101 1202001100 201000???1 20?2022??? ?2?10100?0 021100?000 11??002000 0??200000? 100???0000 ??0000?1?1 0000?0?00? 01000????? 0????????? ?????????? ?????????? ?????????? ???90000?0 ?100011100 201101?0?1 102????0?0 ?01?0??0?0 00??71???? ??????1111 1310100110 ??01101[01]01 10[01]0101110 000??????? ?????????? ??0000000? 0??

*Varanus varius* 3110010001 1200001001 0130011110 1010000100 111100110? 12?1002100 110?000011 0111010000 0000000110 0011021001 100?101111 1100101211 0001010000 0000002001 10000?1010 1010000011 2100000001 1010000000 01120?0001 0111110001 000?100101 1202001100 201000???? ?????????? ?????????? ?????????? ?????????? ????00000? 100????000 ?1??00?1?1 0000?????? 000?0????? 0????????? ?????????? ?????????? ?????????? ???90????? ?00?01?100 101100?0?1 ?12??02110 ?01?0??0?0 00??700??? ??11111011 1300101010 ??01100?01 00[12]010?000 011011[23]1[23]0 0001000[01]00 ??[01]000000? 013

*Varanus yuwonoi* ?????????? ?????????? ?????????? ?????????? ?????????? ?????????? ?????????? ?????????? ?????????? ?????????? ?????????? ?????????? ?????????? ?????????? ?????????? ?????????? ?????????? ?????????? ?????????? ?????????? ?????????? ?????????? ?????????? ?????????? ?????????? ?????????? ?????????? ?????????? ?????????? ?????????? ?????????? ??0??????? ?????????? ?????????? ?????????? ?????????? ???8?????? ?1???????? ?????????? ?????????? ?????????? ?0???1???? ??????1111 11?0??1??? ?????????? ?????????? ?????????? ?????????? ????00000? 013

Yale Quarry varanid ?????????? ?????????? ?????????? ?????????? ?????????? ?????????? ?????????? ?????????? ?????????? ?????????? ?????????? ?????????? ?????????? ?????????? ?????????? ?????????? ?????????? ?????????? ?????????? ?????????? ?????????? ?????????? ?????????1 2?111?2??? ?????????? ?????????? ?????????? ?????????? ?????????? ?????????? ?????????? ?????????? ?????????? ?????????? ?????????? ?????????? ???6?????? ?????????? ?????????? ?????????? ?????????? ?????????? ?????????? ?????????? ?????????? ?????????? ?????????? ?????????? ?????????? ???

Birket Qarun ‘*Varanus*’ ?????????? ?????????? ?????????? ?????????? ?????????? ?????????? ?????????? ?????????? ?????????? ?????????? ?????????? ?????????? ?????????? ?????????? ?????????? ?????????? ?????????? ?????????? ?????????? ?????????? ?????????? ?????????? ?????????1 2?010?2??? ???10????? ?????????? ?????????? ?????????? ?????????? ?????????? ?????????? ?????????? ?????????? ?????????? ?????????? ?????????? ???6?????? ?????????? ?????????? ?????????? ?????????? ?????????? ?????????? ?????????? ?????????? ?????????? ?????????? ?????????? ?????????? ???

Jebel Qatrani ‘*Varanus*’ ?????????? ?????????? ?????????? ?????????? ?????????? ?????????? ?????????? ?????????? ?????????? ?????????? ?????????? ?????????? ?????????? ?????????? ?????????? ?????????? ?????????? ?????????? ?????????? ?????????? ?????????? ?????????? ?????????1 20121?2??? 1?????0??0 0011?????? ?????????? ?????????? ?????????? ?????????? ?????????? ?????????? ?????????? ?????????? ?????????? ?????????? ???6?????? ?????????? ?????????? ?????????? ?????????? ?????????? ?????????? ?????????? ?????????? ?????????? ?????????? ?????????? ?????????? ???

*Varanus marathonensis* (holotype vertebra) ?????????? ?????????? ?????????? ?????????? ?????????? ?????????? ?????????? ?????????? ?????????? ?????????? ?????????? ?????????? ?????????? ?????????? ?????????? ?????????? ?????????? ?????????? ?????????? ?????????? ?????????? ?????????? ?????????1 2?220?2??? ?????????? ?????????? ?????????? ?????????? ?????????? ?????????? ?????????? ?????????? ?????????? ?????????? ?????????? ?????????? ???4?????? ?????????? ?????????? ?????????? ?????????? ?????????? ?????????? ?????????? ?????????? ?????????? ?????????? ?????????? ?????????? ???

*Varanus amnhophilis* ?????????? ?????????? ?????????? ?????????? ?????????? ?????????? ?????????? ?????????? ?????????? ????02?001 ?????????? ??00??12?? ??????010? ?0?????001 0?0000?010 1??00????1 ?000?0?00? ??????0??? ?????????? ??1??????? ??0??0???? ?????????? ?????????1 20220?2??? 1?1101???? ?????????0 1????????? ?????????? ?????????? ?????????? ?????????? ?????????? ?????????? ?????????? ?????????? ?????????? ???4??0??? ?????????? ?????????1 ?????1??10 ?????????? ?????????? ??0110???? ?????????? ?????????? ?????????? ?????????? ?????????? ?????????? ???
