# Supplementary material for: Earliest Example of a Giant Monitor Lizard (Varanus, Varanidae, Squamata)
Source: PLoS One. 2012 Aug 10;7(8):e41767. doi: 10.1371/journal.pone.0041767 (PMC3416840; doi:10.1371/journal.pone.0041767)
Supplement: TEXT S1 — COMPARATIVE MATERIAL. Observations on the following specimens were used for this study. Institutional abbreviations: AMNH, American Museum of Natural History; BSP, Bayerische Staatssammlung für Paläontologie und Geologie; BMNH PR, Natural History Museum, London (Great Britain); GM, Geiseltal Museum of the Martin-Luther Universität in Halle/Saale (Germany); UF, University of Florida, Florida State Museum; ZPAL, Zoological Institute of Paleobiology, Polish Academy of Sciences, Warsaw (Poland). (DOC) [file pone.0041767.s002.doc]

**Earliest example of a giant monitor lizard (*Varanus*, Varanidae, Squamata)**

Jack L. Conrad^1^

Ana M. Balcarcel^2^

Carl M. Mehling^2^

^1^ Anatomy Department, New York College of Osteopathic Medicine, Old Westbury, NY

^2^ Department of Vertebrate Paleontology, American Museum of Natural History, New York, NY

**SUPPORTING INFORMATION**

**TEXT S2: COMPARATIVE MATERIAL**

Observations on the following specimens were used for this study. Institutional abbreviations: AMNH, American Museum of Natural History; BSP, Bayerische Staatssammlung für Paläontologie und Geologie; BMNH PR, Natural History Museum, London (Great Britain); GM, Geiseltal Museum of the Martin-Luther Universität in Halle/Saale (Germany); UF, University of Florida, Florida State Museum; ZPAL, Zoological Institute of Paleobiology, Polish Academy of Sciences, Warsaw (Poland).

*Adriosaurus suessi* BMNH PR 2867; *Aigialosaurus dalmaticus*, BSP 1902 II 501; *Aiolosaurus oriens* IGM 3/171; *Bahndwivici ammoskius* FMNH PR 2260; *Cherminotus longifrons* ZPAL MgR-III/59, ZPAL MgR-III/67; *Clidastes propython* FMNH PR 38, FMNH P27324; *Clidastes* sp. AMNH FR14791; *Coniasaurus crassidens* BMNH PR 23421; *Coniasaurus gracilodens* BMNH PR 44141; *Dolichosaurus longicollis* BMNH PR 49002; *Eosaniwa koehni* GM XXXVIII/57; *Lanthanotus borneensis* FMNH 130981, FMNH 134711; *Necrosaurus cayluxi* BMNH PR 3486; *Paravaranus angustifrons* ZPAL MgR-I/67; *Proplatynotia longirostrata* ZPAL MgR-I/68; *Shinisaurus crocodilurus* FMNH 233130, FMNH 234242; UF 57112, UF 61149, UF 61685, UF 62315, UF 62316, UF 62497, UF 62536, UF 62578, UF 68203; *Telmasaurus grangeri* AMNH FR6643; *Varanus acanthurus* FMNH 218083, FMNH 98935; *Varanus albigularis* AMNH R47726, FMNH 17142, FMNH 22354; *Varanus beccari* AMNH R 141072; *Varanus bengalensis* FMNH 22495; *Varanus doreanus* YPM R 11061, YPM R 13989; *Varanus dumerilii* FMNH 223194, FMNH 228151; *Varanus exanthematicus* FMNH 212985; *Varanus flavescens* AMNH R 77646; *Varanus gouldii* FMNH 250434; *Varanus griseus* FMNH 31380; *Varanus indicus* AMNH R 58389, AMNH R 142623; *Varanus komodoensis* AMNH R37908, FMNH 22199, FMNH 22200; *Varanus niloticus* AMNH R10524, AMNH R74603, FMNH 12300, FMNH 17144, FMNH 17145, FMNH 17146, FMNH 22084, FMNH 22496, FMNH 45807; *Varanus oliveaceus* FMNH 223181; *Varanus ornatus* FMNH R 45087; *Varanus prasinus* FMNH 229907; *Varanus prisca* AMNH FR-1968, AMNH FR-6302, AMNH FR-6303, AMNH FR-6304; *Varanus rudicollis* AMNH R-141071; *Varanus salvadorii* AMNH R59873; *Varanus salvator* AMNH R142471 FMNH 22204, FMNH 31320, FMNH 31358
