# Supplementary material for: Earliest Example of a Giant Monitor Lizard (Varanus, Varanidae, Squamata)
Source: PLoS One. 2012 Aug 10;7(8):e41767. doi: 10.1371/journal.pone.0041767 (PMC3416840; doi:10.1371/journal.pone.0041767)
Supplement: TEXT S2 — CHARACTERS USED IN THE PHYLOGENETIC ANALYSIS. Morphological characters and character-states used in the phylogenetic analysis. (DOC) [file pone.0041767.s003.doc]

**Earliest example of a giant monitor lizard (*Varanus*, Varanidae, Squamata)**

Jack L. Conrad^1^

Ana M. Balcarcel^2^

Carl M. Mehling^2^

^1^ Anatomy Department, New York College of Osteopathic Medicine, Old Westbury, NY

^2^ Department of Vertebrate Paleontology, American Museum of Natural History, New York, NY

**SUPPORTING INFORMATION**

**TEXT S3: CHARACTERS USED IN THE PHYLOGENETIC ANALYSIS**

Morphological characters and character-states used in the phylogenetic analysis.

1. Skull, percentage of total length made up by antorbital snout (DeBraga and Carroll, 1993; character 2): (0) <30%; (1) = or >30%; (2) >45%; (3) >50%.

2. Skull, rostrum anterior to the septomaxilla (Conrad, 2008): (0) short, absent; (1) four tooth positions long or more.

3. Skull, muzzle shape (Norell and Gao, 1997; character 33): (0) tapering; (1) blunt and rounded; (2) squared added (Kearney, 2003; character 1).

4. Skull, canthal crest (McDowell and Bogert, 1956): (0) absent; (1) present, strongly developed.

5. Skull, interorbital septum (Kearney, 2003; character 27): (0) present; (1) absent.

6. Nares, posterior elongation (Estes et al., 1988; character 2): (0) absent; (1) present.

7. Dermal sculpturing (Estes et al., 1988; character 129): (0) "crawling" or "worm-like" sculpture; (1) pitted; (2) bumps/hornlets.

8. Dermal sculpturing, maxilla (Conrad and Norell, 2006; character 5): (0) absent; (1) present.

9. Dermal sculpturing, prefrontal (Conrad and Norell, 2006; character 6): (0) absent; (1) present.

10. Dermal sculpturing, parietal/frontal (Estes et al., 1988; character 129): (0) absent; (1) present on frontal and parietal.

11. Premaxilla, fusion into single element (Lee and Caldwell, 2000; character 1): (0) absent, paired premaxillae; (1) present.

12. Premaxilla, mediolateral breadth of nasal process (Conrad, 2008): (0) absent; (1) broad, widest plane; (2) narrow, narrowest plane; (3) narrow at base, spatulate posteriorly.

13. Premaxilla, external contact with frontal (Lee and Caldwell, 2000; character 2): (0) absent, connected by nasals; (1) present; (2) contact overlain by nasals.

14. Premaxilla, incisive process (Gao and Norell, 1998; character 46): (0) single; (1) bipartite; (2) absent.

15. Premaxilla, rostrum anterior to the premaxillary teeth (DeBraga and Carroll, 1993; character 4): (0) absent; (1) present, conical and extending for about 1 tooth position; (2) cylindrical and extending for about the length of two tooth positions.

16. Premaxilla, contact with maxilla (Rieppel and Zaher, 2000; character 3): (0) sutural; (1) non-sutural.

17. Premaxilla-maxilla aperture (Meszoely, 1970): (0) absent; (1) present.

18. Premaxilla, contact with the nasal (Frost and Etheridge, 1989; character 1): (0) premaxilla(e) overlaps the nasal(s); (1) nasal(s) over lap the premaxilla(e); (2) premaxilla(e) does not reach the nasal(s).

19. Nasals, presence as discrete elements (Lee and Caldwell, 2000; character 21): (0) present; (1) absent.

20. Nasals (Estes et al., 1988; character 3): (0) paired; (1) fused.

21. Nasals, internasal contact (Gao and Norell, 1998; character 2): (0) extensive; (1) less than 1/2 their length.

22. Nasal, anterior border (Conrad, 2008): (0) concave, forming the posterior border of the external naris; (1) lacking anterolateral narial process.

23. Nasofrontal suture, articulated shape in dorsal view (Conrad, 2008): (0) M-shaped; (1) frontal forms an anterior wedge; (2) transverse; (3) W-shaped; (4) V-shaped with nasals invading frontal suture; (5) contact primarily ligamentous; (6) U-shaped, with nasals arching posteriorly.

24. Nasofrontal fontanelle (Conrad, 2008): (0) absent; (1) present.

25. Maxilla, anteromedial process lying between vomers and premaxillae (Estes et al., 1988; Gekkota character 12): (0) absent; (1) present; (2) present and extensive, blocking premaxilla-vomer contact.

26. Maxilla, strong medial processes behind the nasal process of the premaxilla (Conrad, 2008): (0) absent; (1) present, separation subequal to the breadth of the nasal process of the premaxilla.

27. Maxillae, contact at midline behind nasal process of premaxilla (Frost and Etheridge, 1989; character 2): (0) absent; (1) present.

28. Maxilla, apex/middle of the nasal process (Rieppel and Zaher, 2000; character 5): (0) at or anterior to midpoint of maxilla; (1) posterior to midpoint of maxilla; (2) dorsal and ventral margins subparallel (Tchernov et al., 2000); (3) maxilla anteroposteriorly very short, absence of a nasal process implicit.

29. Maxilla, nasal process inclination (Conrad, 2008): (0) steeply inclined, posterior border of the naris distinct from ventral border; (1) weakly inclined, posterior border of the naris not distinct from the ventral border.

30. Maxilla, overlap of prefrontal (Conrad, 2008): (0) only anteriorly; does not include a supraorbital component; (1) extensive, extends beyond the lacrimal and/or lacrimal foramen.

31. Maxilla, contact with vomer posterior to the fenestra vomoeronasalis externa (Estes et al., 1988; character 42): (0) absent; (1) present.

32. Maxilla, palatine flange (Conrad, 2008): (0) medial flaring absent; (1) medially flared from the lateral border of the internal nares; (2) present, expanded posteromedially beyond the posterolateral process of the maxilla.

33. Maxilla, posterior extent of tooth row (Estes et al., 1988; character 27): (0) beyond anterior 1/4 of orbit; (1) terminates at anterior border of orbit.

34. Prefrontal, dorsolateral tuberosity (Conrad, 2008): (0) absent; (1) present.

35. Prefrontal, supraorbital ridge (DeBraga and Carroll, 1993; character 13): (0) absent; (1) present.

36. Prefrontal, pares frontales contact at midline (Grismer, 1988): (0) absent; (1) present.

37. Prefrontal, contact with postorbitofrontal (Estes et al., 1988; character 5): (0) absent; (1) present.

38. Prefrontal, blocks contact between maxilla and nasal (Gao and Norell, 1998; character 10): (0) absent; (1) present, extends anteriorly to the naris; (2) present, contacts the premaxilla.

39. Prefrontal, contact with jugal (Rieppel, 1980; character 24): (0) absent; (1) present.

40. Prefrontal, subpalpebral fossa (Conrad, 2008): (0) absent; (1) present.

41. Lacrimal (Estes et al., 1988; Gekkota character 28): (0) present, large and extending for more than 1/2 the distance to the external naris; (1) present, discrete, and limited to orbital margin; (2) present on orbital margin, but fused to the prefrontal; (3) absent; (4) present, but reduced to a tiny nubbin lying posterior to the lacrimal foramen.

42. Lacrimal, posterolateral flange (Conrad, 2008): (0) absent; (1) present.

43. Lacrimal, foramen (Rieppel and Zaher, 2000; character 10): (0) single; (1) double.

44. Lacrimal foramen, size (Frost and Etheridge, 1989; character 6): (0) small; similar in size to palatine foramen; (1) large; approximately 2 times the smallest diameter of the palatine foramen or more.

45. Jugal (Rieppel and Zaher, 2000; character 12): (0) present; (1) absent.

46. Jugal, anterior extension (Lee and Caldwell, 2000; character 18): (0) no further than if forming the anterior border of the orbit; (1) extends anteriorly beyond the margin of the orbit.

47. Jugal, shape (Gao and Norell, 1998; character 13): (0) angulated; (1) little angulation; curved; (2) reduced to a small splint barely extending beyond the posterior margin of the maxilla; (3) straight posterodorsal bar without a strong suborbital bar.

48. Jugal, posteroventral process (Gao and Norell, 1998; character 14): (0) present, well-defined; (1) absent.

49. Jugal, postorbital branch (McDowell and Bogert, 1956): (0) without anterior or posterior flanges; (1) dilated.

50. Jugal, postorbital process rugosities (Gao and Norell, 1998; character 17): (0) absent; (1) present.

51. Jugal, contact with postorbitofrontal (Estes et al., 1988; character 32): (0) present; (1) absent.

52. Jugal, relationship to maxilla (Conrad, 2006a; character 14): (0) mostly medially; (1) mostly dorsally; (2) mostly posterior to the maxilla.

53. Jugal -squamosal contact (Estes et al., 1988; character 18): (0) absent; (1) present.

54. Quadratojugal (Conrad, 2008): (0) present; (1) absent.

55. Frontals (Estes et al., 1988; character 6): (0) separate in adults; (1) fused in adults.

56. Frontal, anterior constricted neck (DeBraga and Carroll, 1993; character 19): (0) absent; (1) present.

57. Frontals, shape as a unit (Conrad and Norell, 2006; character 33): (0) anterior and posterior borders subequal in width; (1) trapezoid; (2) concave lateral margins, minimum width less than 3/5 posterior border width; (3) tapering posteriorly; (4) pentagonal with an anterior tapering point; (5) square posteromedially with large anterolateral acuminate projection.

58. Frontals, constriction between orbits (Estes et al., 1988; character 7): (0) absent, interorbital margin linear; (1) present, anterior portion of the frontal is hourglass shaped.

59. Frontal, dorsal keel (Bell, 1997; character 12): (0) absent; (1) low, poorly developed; (2) tall, well-developed.

60. Frontal, dorsoventral inflation (Conrad, 2008): (0) absent; (1) present.

61. Frontals, subolfactory processes (Estes et al., 1988; character 10): (0) ventral downgrowths; (1) partly surrounding the olfactory bulbs anteriorly; (2) partly surrounding the olfactory bulbs posteriorly; (3) contact the parabasisphenoid (Rieppel and Zaher, 2000; character 54).

62. Frontals, subolfactory processes contact at midline (Estes et al., 1988; character 10): (0) absent; (1) present.

63. Frontal, medial pillar of the frontal separating the olfactory tracks from one another (Rieppel, 1980): (0) absent; (1) present.

64. Frontals, contact of medial pillar and lateral subolfactory flanges ([1]): (0) absent; (1) present.

65. Frontals, contact the maxilla anteriorly ([2]; Scincidae character 4): (0) absent; (1) present.

66. Frontals, participation in the orbitonasal foramen ([3]; character 7): (0) absent, prefrontals with large contributions; (1) present, prefrontals blocked from the orbitonasal fenestra.

67. Frontal, contact with palatines (Gauthier, 1982; character 82): (0) absent; (1) present.

68. Frontal, invaded by external nares (Bell, 1997; character 5): (0) absent; (1) present.

69. Frontals, parietal tabs (Caldwell, 1999; character 9): (0) absent; (1) present; (2) present, elaborated into dorsomedial extensions on top of the parietals; (3) frontal and parietal fusion.

70. Frontoparietal suture, dorsal view (Evans and Barbadillo, 1998; character 131): (0) U-shaped, anteriorly arched; (1) transverse; (2) W shaped; (3) U-shaped, posteriorly arched.

71. Frontoparietal fontanelle (nov?): (0) absent; (1) present.

72. Parietal, lateral flange at the frontoparietal suture (Conrad, 2008): (0) no lateral flange whatsoever; (1) gently curved, laterally tapering; (2) with broad, squared, lateral tabs such that the postfrontal margin of the frontal is parallel with the postfrontal margin of the parietal.

73. Parietals: (0) paired; (1) fused.

74. Parietal, frontal tabs (Caldwell, 1999; character 17): (0) absent; (1) present within the contact or dorsally; (2) present on the ventral surface.

75. Parietal, median adductor crest expressed as a keel (Frost and Etheridge, 1989; character 10): (0) absent; flat parietal table extends to the posterior margin; (1) present.

76. Parietal, decensus parietalis (Estes et al., 1988; character 23): (0) weakly developed/absent; (1) present as anteroposteriorly elongate crest; (2) present, anteroposteriorly narrow ventral projection.

77. Parietal, pineal foramen (Estes et al., 1988; character 26): (0) within parietal; (1) within frontal; (2) at frontoparietal suture; (3) absent.

78. Parietal fossa, posterior margin (Conrad, 2008) : (0) open, crests extend posterolaterally; (1) closed, crests meet at midline; (2) absent.

79. Parietal, posterior flange (not associated with a sagittal, jaw adductor, crest) (Conrad, 2008): (0) absent; (1) present.

80. Parietal, supratemporal processes length from the level of the parietal notch compared to the parietal anterior to that point (Gauthier, 1982; character 30): (0) greater than 1/2; (1) less than 1/2; (2) absent.

81. Parietal, dorsal margin of the supratemporal process ([4]; character 33): (0) narrow, bladelike; (1) broad and flat.

82. Parietal, transverse posterior margin between the supratemporal processes (Conrad, 2008): (0) present, space between supratemporal processes greater the width of the supratemporal process base; (1) absent, no transverse posterior margin between the supratemporal processes.

83. Parietal, nuchal fossa (Gao and Norell, 2000): (0) absent; (1) present; (2) present and extending substantially onto the skull table.

84. Parietal, contact with supratemporal arch (Caldwell, 1999; character 15): (0) only at the anterior and posterior extremes; (1) increased contact anteriorly and posteriorly; (2) absent.

85. Parietal, contact with the supraoccipital (Tchernov, 2000; character 37): (0) absent or only via the processus ascendens tecti synotica; (1) present, extensive (posttemporal fenestra closed).

86. Parietal, attachment of jaw adductor musculature (Estes et al., 1988; character 54): (0) dorsally; (1) ventrally; (2) reduced; appear to attach only laterally.

87. Supratemporal (Estes et al., 1988; Gekkota character 10): (0) present; (1) absent.

88. Supratemporal, length relative to depth (Conrad, 2008): (0) short, not extending much beyond the jaw suspensorium; (1) elongate, extending well anterior to the jaw suspensorium (more than 3 times as long as deep).

89. Postfrontal/postorbital contact with the frontal/parietal, forking of medial surface (Rieppel and Zaher, 2000; character 25): (0) absent; (1) present.

90. Postorbital/postfrontal, tubercle (McGuire, 1996; character 7): (0) absent; (1) present; (2) present, elaborated into a horn.

91. Postfrontal (Conrad, 2008): (0) present; (1) absent.

92. Postfrontal, shape (Conrad, 2008): (0) anteroposteriorly elongate; not a strong lateral bar; (1) irregular or round and reduced in size; (2) mediolaterally developed bar bordering the supratemporal fenestra and orbit.

93. Postfrontal, contact with the parietal (Estes et al., 1988; character 15): (0) absent; (1) present; (2) present, for more than 1/2 the parietal length.

94. Postorbitofrontal, fusion (Estes et al., 1988; character 14): (0) absent; (1) present.

95. Postorbital (posterior ramus of postorbitofrontal) (Estes et al., 1988; character 16): (0) present; (1) absent.

96. Postorbital, posterior extent (Estes, 1983) : (0) less than 1/2 the length of the supratemporal fenestra; (1) more than 1/2 the length of the supratemporal fenestra; (2) more than 3/4 the length of the supratemporal fenestra; (3) contacts the supratemporal.

97. Postorbital, contribution to the postorbital bar (Estes et al., 1988; character 17): (0) 1/2 or more; (1) less than 1/2.

98. Squamosal (Rieppel and Zaher, 2000; character 40) : (0) present; (1) absent.

99. Squamosal, contact with postorbitofrontal (Rieppel and Zaher, 2000; character 38): (0) present; (1) absent.

100. Squamosal, dorsal process (Caldwell, 1999; character 25): (0) present; (1) absent.

101. Palpebral ossification(s) (Estes et al., 1988; character 36): (0) absent; (1) present, a single ossification articulating with or located near the prefrontal; (2) present, multiple ossifications running down the lateral surface of the prefrontal and frontal.

102. Septomaxilla (Estes et al., 1988; Chamaeleonidae character): (0) present; (1) absent.

103. Septomaxilla, medial flange ([5]; character 87): (0) short/absent; (1) long.

104. Septomaxilla, contact with the osseous nasal cavity roof ([6]; character 54): (0) absent; (1) present.

105. Palate, orientation of the ectopterygoid and the pterygoid transverse process ([7]): (0) mostly oriented mediolaterally; (1) oriented at >30 degree angle from the perpendicular to the sagittal.

106. Vomers, fusion (Conrad, 2008): (0) absent; (1) present.

107. Vomer, shape (Rieppel and Zaher, 2000; character 93): (0) platelike; (1) rod-like.

108. Vomer, contact with the palatine ([8]): (0) subequal in breadth to the contact with the maxilla; (1) about 1/2 the breadth of the contact with the maxilla; movable; (2) reduced into a narrow peg from the vomer contacting the palatine; (3) absent (TR47).

109. Vomers, teeth ([9]; character 22): (0) present; (1) absent.

110. Palatines, medial expansion anteriorly (Gauthier, 1982; character 84): (0) absent; (1) present.

111. Palatine, length (Gao and Norell, 1998; character 51): (0) longer than wide; (1) subequal in length and width; (2) extremely foreshortened, much deeper and broader than long.

112. Palatine, length relative to the vomer (Rieppel and Zaher, 2000; character 98): (0) as long as; (1) 2/3 as long as.

113. Palatines, choanal groove (Estes et al., 1988; character 44): (0) very short/absent; (1) distinct, elongate.

114. Palatine, secondary palate formed around choanal groove (Estes et al., 1988; character 43): (0) absent; (1) present, ventromedial fold partly hide the choanal groove; (2) present, ventromedial processes hide most or all of the dorsomedial processes; (3) contact at midline.

115. Palatine, teeth (Estes et al., 1988; character 82): (0) present, patches; (1) absent; (2) present, single line.

116. Pterygoid, contact with jugal (Gao and Norell, 1998; character 54): (0) absent; (1) present.

117. Pterygoid, ventromedial process (Gao and Norell, 1998; character 32): (0) absent; (1) present.

118. Pterygoid, teeth (Estes et al., 1988; character 83): (0) arranged in multiple rows or patches; (1) in a single line; (2) absent.

119. Pterygoid, contact with vomer: (0) present; (1) absent.

120. Interpterygoid vacuity, midline contact of vomers (Conrad, 2008): (0) present, contact for their length; (1) present, invaded by interpterygoid vacuity; (2) present, contact anteriorly and posteriorly; (3) absent.

121. Interpterygoid vacuity, midline contact of palatines (Conrad, 2008): (0) absent; (1) present; (2) vomer-pterygoid contact present.

122. Interpterygoid vacuity, midline contact of pterygoids (Conrad, 2008): (0) absent; (1) present.

123. Interpterygoid vacuity, broadest point vs distance from basicranial joint to quadrate (Conrad, 2008): (0) 2/5 or greater; (1) 1/3 or less; (2) closed.

124. Ectopterygoid, contact with palatine anterior to the suborbital fenestra (Estes et al., 1988; character 45): (0) absent; (1) present; (2) present, contact broader than suborbital fenestra; (3) present, closes suborbital fenestra (Kearney, 2003; character 99).

125. Ectopterygoid, contact with palatine posterior to the suborbital fenestra (Gao and Norell, 2000): (0) absent; (1) present.

126. Ectopterygoid, lateral exposure behind maxilla (McDowell and Bogert, 1956): (0) absent; (1) present.

127. Braincase, ventral sagittal ridge on the basisphenoid and basioccipital (Tchernov, 2000; character 77): (0) absent; (1) present.

128. Braincase, "X ossicle" present on the ventral surface (Norell and Gao, 1997; character 27): (0) absent; (1) present.

129. Braincase, fenestra ovalis location (Norell and Gao, 1997; character 25): (0) above/slightly posterior to the spheno-occipital tubercle; (1) anterior to the spheno-occipital tubercle.

130. Braincase, orbitosphenoid (Kearney, 2003; character 105): (0) absent; (1) present, azygous; (2) present, paired orbitosphenoid.

131. Braincase, occipital condyle (Estes et al., 1988; Gekkota character 9): (0) single unit made of basioccipital and exoccipitals; ovoid or subovoid; (1) bipartate, constructed of exoccipitals; (2) single unit made of basioccipital and exoccipitals; U-shaped; (3) formed only by the basioccipital.

132. Braincase, anterior extension of crista prootica (Estes et al., 1988; Gekkota character 52): (0) terminates at inferior process; (1) extends onto the basipterygoid process; (2) crista prootica absent.

133. Braincase closure (Kearney, 2003; character 29): (0) open; (1) parietal downgrowths; (2) parietal downgrowths and anterior extensions of prootics.

134. Epipterygoid (Estes et al., 1988; character 47): (0) present; (1) absent.

135. Supraoccipital, processus ascendens tecti synotici (Estes et al., 1988; Gekkota character 11): (0) present; (1) absent.

136. Prootic, supratrigeminal process (Estes et al., 1988; character 50): (0) absent or faint ridge; (1) distinct, anterior process visible in lateral view; (2) closes trigeminal foramen.

137. Prootic, crista alaris (Kearney, 2003; character 79): (0) absent; (1) present, short and not extending further anteriorly than the inferior process of the prootic; (2) present, elongate and overhanging the inferior process of the prootic.

138. Prootic, crista prootica (Rieppel and Zaher, 2000; character 66): (0) well-developed; lateral and ventral aspects; (1) reduced; extending mostly laterally.

139. Prootic, perforation of the crista prootica: (0) absent; (1) present.

140. Prootic, entocarotid fossa (Gao and Norell, 1998; character 30): (0) present as distinct fossa within the recessus vena jugularis; (1) reduced/absent.

141. Prootic, facial foramen (Norell and Gao, 1997; character 20): (0) single; (1) double.

142. Laterosphenoid (Tchernov, 2000; character 65): (0) absent; (1) present.

143. Sphenoid, posterolateral flanges laterally overlying basioccipital (B-B83): (0) absent; (1) present; (2) fusion of basioccipital to the basisphenoid.

144. Sphenoid, lateral head vein enclosure by the sphenoid (Estes et al., 1988; character 52): (0) absent/incomplete; (1) complete.

145. Sphenoid, anterior opening of Vidian canal (Norell and Gao, 1997; character 18): (0) ventral to dorsum sellae; (1) on the roof of the dorsum sellae; (2) on the ventral surface of the parabasisphenoid ([10]; character 45)

146. Sphenoid, relationship with the posterior opening of the Vidian canal (Estes et al., 1988; character 53): (0) houses it; (1) share it with the prootic; (2) at the prootic contact with the X-ossicle; (3) shared wtih parietal.

147. Parasphenoid, teeth (Gauthier, 1988; character 31): (0) present; (1) absent.

148. Basipterygoid processes, length (Rieppel and Zaher, 2000; character 74): (0) long, extending beyond main body of the basisphenoid; (1) short, expressed as short nubbins that are more than 2 times as wide as long and not extending anterior to the main body of the basisphenoid; (2) absent.

149. Basioccipital, crista tuberalis development (Rieppel and Zaher, 2000; character 79): (0) medially concave; (1) medially flat, inclusion in the paroccipital process more lateral than on the spheno-occipital tubercle; (2) expanded into a lateral process; (3) absent.

150. Basioccipital, spheno-occipital tubercle (Norell and Gao, 1997; character 23): (0) short and ventrally directed; (1) elongate and posterolaterally directed; (2) absent.

151. Basioccipital, location of the spheno-occipital tubercle (Rieppel and Zaher, 2000; character 76): (0) posteriorly, crista tuberalis nearly vertical; (1) anteriorly, crista tuberalis posterodorsally inclined.

152. Basioccipital, canal or groove for basalar artery (DeBraga and Carroll, 1993; character 34): (0) absent; (1) present.

153. Otooccipital, hypoglossal foramen (Rieppel and Zaher, 2000; character 69): (0) separated from vagus foramen; (1) internally subdivided or confluent.

154. Otooccipital, occipital recess closure (Rieppel and Zaher, 2000; character 70): (0) open; (1) closed.

155. Otooccipital, occipital recess (Conrad, 2008): (0) hidden by spheno-occipital tubercle in ventral view; (1) visible in ventral view.

156. Stapes, internal (quadrate) process (Estes et al., 1988; character 141): (0) absent; (1) present.

157. Stapes, shape of shaft (Tchernov, 2000; character 60): (0) straight; (1) angulated.

158. Extracolumella, anterior elongation (Ke03-82): (0) absent; (1) present.

159. Quadrate, suspension (Rieppel and Zaher, 2000; character 49): (0) monimostylic; (1) streptostylic; (2) suspended mainly from supratemporal; (3) suspended mainly from opisthotic; (4) suspended mainly from squamosal.

160. Quadrate, pterygoid lappet (Estes et al., 1988; character 37): (0) present; (1) absent.

161. Stapes, position of stapedial artery (Estes et al., 1988; character 145): (0) pierces stapes; (1) anteriorly; (2) posteriorly.

162. Quadrate, tympanic crest (Rieppel and Zaher, 2000; character 51): (0) >/= posterior crest; (1) shorter than posterior crest; (2) absent.

163. Quadrate, suprastapedial process (Carooll and DeBraga, 1992; character 2): (0) absent; (1) present.

164. Quadrate, infrastapedial arch (DeBraga and Carroll, 1993; character 41, 43): (0) absent; (1) present; (2) present, contacts the suprastapedial process; (3) fusion of the suprastapial process and the infrastapedial arch.

165. Extracolumellar tissue, calcification (Lee and Caldwell, 2000; character 65): (0) absent; (1) present.

166. Mandible; fusion of articular, prearticular, and surangular (Rieppel and Zaher, 2000; character 129): (0) absent; (1) articular-prearticular, surangular.

167. Mandible, symphysis (Rieppel and Zaher, 2000; character 110): (0) present; (1) absent.

168. Mandible, adductor fossa orientation (Rieppel and Zaher, 2000; character 137): (0) medial margin of low and rounded; (1) distinct vertical flange.

169. Mandible, adductor fossa expansion (Estes et al., 1988; character 81): (0) absent; (1) present.

170. Mandible, intramandibular septum (Rieppel and Zaher, 2000; character 116): (0) terminates more than two tooth widths anterior to the last tooth position; (1) terminates at, or nearly at, the posteriormost tooth position.

171. Mandible, intramandibular septum ventral margin (Meszoely, 1970): (0) absent; (1) posteroventral margin sutured; (2) posteroventral margin free.

172. Mandible, anterior surangular foramen (Conrad, 2008): (0) present; (1) absent.

173. Mandible, external border of the anterior surangular foramen (Conrad, 2008): (0) formed only by the surangular; (1) margin with coronoid contribution; (2) with dentary contribution; (3) with coronoid and dentary contribution; (4) anterior surangular foramen absent; (5) within coronoid.

174. Mandible, groove associated with anterior surangular foramen (Conrad, 2008): (0) absent; (1) present.

175. Mandible, posterior mylohyoid foramen (Conrad, 2008): (0) present; (1) absent.

176. Mandible, position of posterior mylohyoid foramen (Frost and Etheridge, 1989; character 24): (0) anterior to the coronoid apex; (1) posterior to the coronoid apex.

177. Mandible, glenoid (DeBraga and Caroll, 1993; character 54): (0) formed at least primarily by articular; (1) formed equally by articular and surangular.

178. Dentary, shape of long axis (DeBraga and Carroll, 1993; character 67): (0) ventrally convex; (1) straight.

179. Dentary, anteroventral surface (Gauthier, 1982; character 59): (0) narrow, depth greater than width; (1) broader than tall with splenial and Meckel's canal slightly visible laterally.

180. Dentary, posterior extent (Evans and Barbadillo, 1998; character 213): (0) to the level of the posterior margin of the coronoid process (eminence); (1) extends to the midpoint of mandible between the coronoid eminence and the articular condyle.

181. Dentary, Meckel's canal (Estes et al., 1988): (0) open; (1) partly closed; (2) closed and fused.

182. Dentary, subdental shelf (Estes et al., 1988; character 58-59): (0) present; (1) absent; (2) present, enlarged (e58).

183. Dentary, anterior inferior alveolar foramen (Estes, 1964): (0) does not contribute; (1) contributes to dorsal border; (2) forms anterior and dorsal border; (3) foramen indistinct from other medial mandibular foramina.

184. Dentary, posterodorsal coronoid process(es) (Rieppel and Zaher, 2000; character 113): (0) large; (1) small; (2) absent.

185. Dentary, sub-coronoid and surangular processes (Estes et al., 1988; character 63): (0) absent; (1) present; (2) intramandibular hinge.

186. Dentary, angular process compared to surangular process (Gauthier, 1982; character 41-): (0) terminate at about the same posterior level; (1) terminates anteriorly; (2) extends posteriorly.

187. Dentary, principle support (Rieppel and Zaher, 2000; character 119): (0) coronoid, surangular, and prearticular; (1) prearticular; (2) surangular.

188. Splenial (Kearney, 2003; character 125): (0) present; (1) absent; (2) present, fused to the postdentary bones (Greer, 1985).

189. Splenial, extent of anteromedial walling of Meckel's canal (Estes et al., 1988; character 67): (0) extends for less than 1/2 of the dentary; (1) extends for more than 2/3 of dentary; (2) extends anteriorly to contribute to the mandibular symphysis.

190. Splenial, posterior extent (Estes et al., 1988; character 66): (0) extends posterior to the apex of the coronoid; (1) terminates at, or anterior to, the coronoid apex.

191. Splenial, overlap with postdentary bones (Rieppel and Zaher, 2000; character 121): (0) overlap, no hinge with angular; (1) abutting, splenial receives angular; (2) abutting, angular receives splenial.

192. Coronoid, height of coronoid at process relative to the length of the mandible (Conrad, 2008): (0) short, broad; (1) tall, narrow; (2) absent.

193. Coronoid, posterior extent of the labial flange (Conrad, 2008): (0) absent; (1) extends mostly labially, does not overlap the posterior margin of the coronoid process in lateral view; (2) extends beyond the posterior margin of the coronoid process in lateral view.

194. Coronoid, anterior end (Estes et al., 1988; character 70): (0) clasps the dentary; (1) butts against dentary.

195. Coronoid, ventral margin (Rieppel and Zaher, 2000; character 128): (0) flat or concave; (1) convex.

196. Coronoid, long and low anterior process (Estes et al., 1988; character 69): (0) absent; (1) present.

197. Coronoid, contact with the anterior inferior alveolar foramen (Conrad, 2008): (0) absent; (1) present.

198. Coronoid, posterior overlap by surangular (Estes et al., 1988; character 71): (0) absent; (1) present.

199. Surangular, anterodorsal buttress of coronoid (DeBraga and Carroll, 1993; character 57): (0) absent; (1) present.

200. Surangular, anterior border when disarticulated (Estes et al., 1988; character 61): (0) tapering; (1) expanded anterodorsally with vertical anterior margin.

201. Surangular, anterior extension into mental canal (Ga-75): (0) absent; (1) present.

202. Angular (Rieppel and Zaher, 2000; character 132): (0) present; (1) absent.

203. Prearticular, crest (Estes et al., 1988; character 73-): (0) absent; (1) present.

204. Prearticular, crest with imbedded angular process (Estes et al., 1988; character 73-): (0) absent; (1) present.

205. Articular, retroarticular process (Estes et al., 1988; character 75): (0) posteriorly directed; (1) medially deflected; (2) retroarticular process absent.

206. Articular, medial offset of retroarticular process with lateral notch (Estes et al., 1988): (0) absent; (1) present.

207. Articular, retroarticular process with posterior broadening (Estes et al., 1988; character 78): (0) absent; (1) present.

208. Articular, retroarticular process pit (Estes et al., 1988; character 74): (0) present; (1) absent.

209. Articular, tubercle on the medial margin of retroarticular process (Estes et al., 1988; character 76; Gao and Norell, 2000): (0) absent; (1) present as a tubercle; (2) present, elaborated into a finger-like process.

210. Articular, torsion of retroarticular process (Gao and Norell, 1998; character 70): (0) absent; (1) present; (2) present and strongly twisted.

211. Dentition, spacing (Conrad, 2008): (0) closely spaced; (1) widely separated; spaces between tooth bases greater than 1/2 the width of a tooth shaft; (2) tightly packed with tooth shafts contacting for most of their height; ctenodont.

212. Dentition, form of teeth (Gauthier, 1982; character 34) : (0) straight, pointed; (1) triangular; (2) trenchant, curved; (3) incipient cusps on posterior teeth; (4) teeth with multiple crowns; (5) globidont; (6) squared dorsal margin; (7) mediolaterally expanded with transverse cutting edges.

213. Dentition, waist on marginal teeth (Conrad, 2008): (0) absent; (1) present.

214. Dentition, posterior marginal tooth implantation (Rieppel and Zaher, 2000; character 146): (0) labially pleurodont; (1) acrodont; (2) modified pleurodont; (3) enclosed by expanded interdental ridge; (4) subacrodont; (5) thecodont; (6) anteriorly pleurodont, posteriorly acrodont.

215. Dentition, caniniform teeth (Estes et al., 1988; Agamidae character 1): (0) absent; (1) present.

216. Dentition, anterior marginal teeth (DeBraga and Carroll, 1993; character 53): (0) generally perpendicular to the long axis of the jaw; (1) procumbent.

217. Dentition, expanded bases on marginal teeth (Rieppel and Zaher, 2000; character 149; state 2 added by me): (0) absent; (1) present, main shafts of teeth somewhat separated.

218. Dentition, plicidentine (Estes et al., 1988; character 86): (0) absent; (1) present.

219. Dentition, crown striations (Meszoely, 1970): (0) absent; (1) present.

220. Dentition, venom groove (Pregill et al., 1986; character 30): (0) absent; (1) present; (2) present, tubular fang.

221. Dentition, replacement (Estes et al., 1988; character 85): (0) develop lingually, large resorption pit; (1) posterolingually, resorption pit; (2) posterolingually, no resorption pit; (3) addition at the posterior end of the tooth row (Reynoso, 1996: 27).

222. Dentition, enlarged median premaxillary tooth more than half again the diameter of the other premaxillary teeth (Kearney, 2003; character 115): (0) absent; (1) present.

223. Dentition, premaxillary teeth compared to maxillary teeth (Rieppel and Zaher, 2000; character 156): (0) similar; (1) markedly smaller; (2) absent; (3) enlarged into fangs.

224. Dentition, maxillary teeth (Conrad, 2008): (0) present; (1) absent.

225. Dentition, dentary teeth (Conrad, 2008): (0) present; (1) absent.

226. Dentition, chisel shaped posterior teeth (Meszoely, 1970): (0) absent; (1) present.

227. Hyoid, second ceratobranchial (Estes et al., 1988; character 91): (0) present; (1) absent.

228. Hyoid, second epibranchials (Estes et al., 1988; character 90): (0) present; (1) absent.

229. Epihyal, shape (Estes et al., 1988; Gekkota character -20): (0) small, triangular; (1) large, wing-like.

230. Notochord, in adults (Kluge, 1987; character 2): (0) persistent; (1) obliterated.

231. Vertebrae, centrum morphology (Kluge, 1987; character 1): (0) amphiplatyan; (1) amphicoelous; (2) procoelous.

232. Vertebrae, neural spines (Gao and Norell, 1998; character 78/Kearney, 2003; character 135): (0) short and broad; (1) tall and narrow; (2) absent.

233. Vertebrae, precondylar constriction (Estes et al., 1988; character 94): (0) absent; (1) present, weakly constricted; (2) strongly constricted to less than 80% of the maximum condylar diameter.

234. Vertebrae, obliqueness of condyles (Estes et al., 1988; character 92): (0) absent/weak, posterior apex of condyle visible; (1) moderate, articulating condylar surface slightly visible in ventral view; (2) strong, articulating surface not visible in ventral view.

235. Vertebrae, zygosphene-zygantra (Rieppel and Zaher, 2000; character 169): (0) absent; (1) present, zygosphene articular surface faces dorsolaterally; (2) present, zygosphene articular surfaces face ventrolaterally.

236. Vertebrae, presacral number (Estes et al., 1988; character 105-106): (0) 25 or fewer; (1) 26; (2) 27 or more.

237. Dorsal vertebrae, transverse processes: (0) present; (1) present in anterior presacrals, absent in posterior ones; (2) absent as distinct processes; synapophyses present.

238. Presacral vertebrae, length of transverse processes: (0) short, subequal or shorter than centrum; (1) more than the length of the centrum.

239. Atlas, dorsal margin (Conrad, 2008): (0) horizontal; (1) posteroventrally inclined.

240. Atlas, lateral process (Conrad, 2008): (0) well defined with some posterior overlap of the axis; (1) small, a "hill-like" projection; (2) absent.

241. Cervical vertebrae, length relative to the dorsal vertebrae (L97-103): (0) subequal to or shorter than; (1) more elongate than.

242. Cervical vertebrae (Estes et al., 1988; character 107,108): (0) 8; (1) 7 or fewer; (2) 9; (3) 10 or more.

243. Cervical, intercentra (Estes et al., 1988; character 97): (0) intervertebral; (1) sutured to the posterior part of the preceding centrum; (2) fused to posterior part of preceding centrum; (3) fused to the succeeding centrum (Conrad, 2008); (4) absent.

244. Cervicals, hypapohyseal keel (Evans and Barbadillo, 1998; character 149): (0) absent; (1) present.

245. Dorsal vertebrae, pachyostosis (Lee and Caldwell, 2000; character 196): (0) absent; (1) present.

246. Dorsal vertebrae, intercentra (Evans and Barbadillo, 1998; character 86): (0) present; (1) absent.

247. Sacral vertebrae, functional (DeBraga and Carroll, 1993; character 72): (0) present; (1) absent.

248. Cloacal vertebrae, lymphapophyses (Lee and Caldwell, 2000; character 209): (0) absent; (1) present.

249. Caudal vertebrae, dorsoventral height (including the neural spines and chevrons) (Bell, 1997; character 9): (0) less than 3 times length of centrum; (1) greater than 3 times length of centrum.

250. Caudal vertebrae, zygapophyses and transverse processes (Carroll and DeBraga, 1992; character 5): (0) well developed, zygaphophyses extending more 1/4 the length of centrum; (1) reduced, creating greater flexibility of the trunk and tail.

251. Caudal vertebrae, transverse processes (Estes et al., 1988; character 100-102): (0) single; (1) double, diverging; (2) double converging; (3) absent.

252. Caudal vertebrae, autotomy planes (Estes et al., 1988; character 103): (0) present on transverse process(es); (1) present posterior to the transverse process(es); (2) absent; (3) present anterior to transverse processes.

253. Caudal vertebrae, pedestals for intercentra (Rieppel and Zaher, 2000; character 183): (0) bulge; (1) well-developed.

254. Chevrons, position (Rieppel and Zaher, 2000; character 185): (0) at the posteroventral margin of the centrum; (1) anterior to the posteroventral margin of the centrum; (2) fused to vertebrae (Gao and Norell, 1998; character 83).

255. Ribs, anterior pseudotuberculum (Lee and Caldwell, 2000; character 207): (0) absent; (1) present.

256. Ribs, posterior pseudotuberculum (Lee and Caldwell, 2000; character 208): (0) absent; (1) present.

257. Ribs, expansion and flattening of the anterior presacral ribs (Conrad, 2008): (0) absent; (1) present.

258. Ribs, postxiphisternal inscriptional ribs (Frost & Etheridge, 1989: 40): (0) contacting the dorsal ribs, not contacting at midline; (1) contacting dorsal ribs, one or more pairs confluent at midline; (2) free dorsally, confluent ventrally.

259. Postcloacal bones (Estes et al., 1988): (0) absent; (1) present.

260. Clavicles, shape (Rieppel and Zaher, 2000; character 196): (0) rodlike; (1) expanded proximally with notch or fenestra; (2) absent.

261. Clavicle, shape (Estes et al., 1988; character 116): (0) straight, without angulation; (1) strongly curved/angled.

262. Coracoid, anterior (primary) coracoid emargination (Estes et al., 1988; character 112): (0) absent; (1) present.

263. Coracoid, posterior emargination (Estes et al., 1988; character 113): (0) absent; (1) present.

264. Epicoracoid cartilage, contact with mesoscapula (Estes et al., 1988; character 114): (0) present; (1) absent.

265. Scapula, size relative to the coracoid (DeBraga and Carroll, 1993; character 95): (0) subequal to, or larger than; (1) smaller than; (2) scapula and coracoid absent.

266. Scapula, secondary scapular fenestra formed by a scapular epicoracoid bar (Estes et al., 1988; character 111): (0) absent; (1) present.

267. Sternum, rib attachments (Estes et al., 1988; character 109): (0) five; (1) four; (2) three; (3) two or fewer.

268. Interclavicle (Estes et al., 1988; character 118): (0) present; (1) absent.

269. Interclavicle, anterior process (Estes et al., 1988; character 120): (0) absent; (1) present, single; (2) present, double.

270. Interclavicle, lateral arms (Estes et al., 1988; character 119): (0) present; (1) absent.

271. Sternum (Kearney, 2003; character 147): (0) present, articulates with pectoral girdle; (1) present, reduced and does not articulate wtih pectoral girdle; (2) absent.

272. Sternum, proximity to the lateral arms of the interclavicle (Frost and Etheridge, 1989; character 33): (0) separated by more than 1/3 the posterior procress of the interclavicle; (1) separated by 1/3 or less the length of the posterior process; (2) posterior process of the interclavicle shorter than the one of the lateral arms.

273. Sternum, fontanelle (Estes et al., 1988; character 121): (0) absent; (1) present.

274. Xiphisternum, branching: (0) more than one branching; (1) one branching; (2) unbranched.

275. Humerus, shape (DeBraga and Carroll, 1993; character 104): (0) elongate, sub-cylindrical, distal ends at right angles to one another; (1) flattened; hourglass-shaped; (2) flattened; square, ends expanded but equal; (3) flattened; rhomboid, distal end more expanded; (4) absent.

276. Humerus, deltopectoral crest (DeBraga and Carroll, 1993; character 107): (0) single continuous projection; (1) separate, but joined by a lamina; (2) separate, not joined by a lamina.

277. Ectepicondylar foramen (Evans and Barbadillo, 1998; character 103): (0) foramen; (1) groove; (2) absent altogether.

278. Forelimb, zeugopodium : (0) present; (1) absent.

279. Radius, preaxial ridge (DeBraga and Carroll, 1993; character 113): (0) absent; (1) thin, rounded, lamina extending for more than 1/2 the anterior margin; (2) present, rounded and extending for less than 1/2 the anterior edge; (3) present, greatly expanded with a anteroproximal apex.

280. Ulna, articulation with intermedium (DeBraga and Carroll, 1993; character 111): (0) absent; (1) present, no facet; (2) present, with a facet.

281. Carpus, intermedium (Estes et al., 1988; Gekkota character 24): (0) present; (1) absent.

282. Manus, first metacarpal (DeBraga and Carroll, 1993; character 116): (0) similar in robustness to other metacarpals; (1) robust, nearly twice as wide as the other metacarpals.

283. Manus, orientation of the fifth digit relative to the others (DeBraga and Carroll, 1993; character 119): (0) fifth digit not greatly divergent; (1) at greater than 70 degrees from fourth digit.

284. Pelvis (Rieppel and Zaher, 2000; character 207): (0) fused into a single ossification; (1) strongly sutured; (2) nonsutural contacts.

285. Pubis, relative length of the symphysial portion compared to the tubercular portion (Estes et al., 1988; character 124): (0) shorter than; (1) subequal to, slightly longer than; (2) more than 1/2 again as long.

286. Pubis, distal shape (DeBraga and Carroll, 1993; character 125): (0) slender; (1) expanded.

287. Ilium, anterior process (Rieppel and Zaher, 2000; character 208): (0) present; (1) absent.

288. Femur, two distal condyles (DeBraga and Carroll, 1993; character 132): (0) separate; (1) confluent.

289. Femur, shape (DeBraga and Carroll, 1993; character 130): (0) cylindrical with moderately expanded proximal and distal ends; (1) flattened, breadth of distal end more than 1/3 the length of the bone; (2) femur absent.

290. Pes (Conrad, 2008): (0) present with digits and/or metatarsals; (1) absent; (2) tarsal element(s) only.

291. Pes, medial and lateral plantar tubercles on metatarsal V (Rieppel **): (0) even with one another or overlapping levels; (1) lateral tubercle distally placed; (2) lateral tubercle distally placed, approaching condyle; (3) greatly shortened metatarsal V precludes indentification; (4) absent.

292. Astragalus and calcaneum, fusion (-Lee98-215): (0) separate; (1) fused; (2) calcaneum unknown.

293. Egg teeth (Estes et al., 1988; Gekkota character 19): (0) single; (1) double; (2) absent.

294. Femoral/precloacal pores (Estes et al., 1988; character 144): (0) absent; (1) present.

295. Integument, gular fold with distinctive midventral squamation (Frost and Etheridge, 1989; character 47): (0) absent; (1) present.

296. Integument, annular rings (Kearney, 2003; character 3): (0) absent; (1) present on body posterior to head.

297. Integument, scale organ ornamentation (Frost and Etheridge, 1989; character 52/H1993): (0) absent; (1) spinules; (2) spikes.

298. Squamation, cephalic scales (Estes et al., 1988; character 147): (0) absent; (1) small and irregularly shaped; (2) enlarged plates.

299. Squamation, contact between frontoparietal scales (Meszoe70) : (0) absent; (1) present.

300. Squamation, elongate middorsal scales with apices (Estes et al., 1988; character 146): (0) present; (1) absent.

301. Squamation, cycloid scales (Estes et al., 1988; character 148): (0) absent; (1) present.

302. Squamation, cephalic scale fusion (Ke03-5): (0) absent; (1) present.

303. Squamation, imbrication: (0) absent; (1) present.

304. Squamation, lateral fold in body between armor dorsally and ventrally (McDB54): (0) absent; (1) present.

305. Osteoderms, dorsal surface of the body (Estes et al., 1988; character 127): (0) absent; (1) present.

306. Integument, dorsal compound osteoderms (Estes, 1983): (0) absent; (1) present.

307. Osteoderms, ventral surface of the body (Estes et al., 1988; character 126): (0) absent; (1) present.

308. Integument, ventral compound osteoderms (Estes, 1983): (0) absent; (1) present.

309. Osteoderms, grooves separating osteoderms on maxilla (Yatkola76-5): (0) absent; (1) present.

310. Squamation, osteodermal thickening (Conrad, 2008): (0) absent, osteoderms thin plates or non-calcified; (1) present, irregularly shaped; (2) present, polygonal mounds; (3) absent, osteoderms circular/vermiculate.

311. Squamation, bony tubercles (Conrad, 2008): (0) absent; (1) present as individual tubercular osteoderms; (2) present as osteoscutes covered with multiple tubercles.

312. Squamation, keeled scales/osteoderms on body: (0) absent; (1) present.

313. Eyeball (Ke00-10): (0) complete and exposed; (1) reduced, covered by a head scale; (2) reduced, not externally visible.

314. Eye, spectacle (fused eyelids) (Kearney, 2003; character 11): (0) absent; (1) present.

315. Eye, scleral ossicles (Kearney, 2003; character 12): (0) present; (1) absent.

316. Glossus, filamentous tongue papillae (Schwenk, 1988; characters 6-9): (0) absent; (1) reticular; (2) peg-like; (3) assymmetrical, forming points.

317. Glossus, division of foretongue (Estes et al., 1988; character 137): (0) absent; (1) notched more than 10% of length; (2) notched more than 20%; (3) notched more than 40%; (4) notched more than 50%; (5) notched more than 50% of length.

318. Glossus, foretongue retracts within hindtongue at zone of invagination (Estes et al., 1988; character 136): (0) absent; (1) present.

319. Glossus, cross-section of tongue (Estes et al., 1988; character 138): (0) rounded and glandular; (1) flattened foretongue; (2) keratinized and mushroom shaped foretongue cross-section.

320. Gland of Gabe (Gao and Norell, 1998; character 102): (0) absent; (1) present.

321. Ear, external ear opening (Kearney, 2003; character 13): (0) present; (1) absent.

322. Inner ear, thickening of the neural limbus of cochlear duct (Estes et al., 1988; Anguimorpha character 14): (0) absent; (1) present.

323. Inner ear, ciliary restraint for hair cells (Estes et al., 1988; character 140): (0) tectorial, lacking sallet systems; (1) tectorial and sallet; (2) more than 1/2 hair cells inertial.

324. Sero mucus glands in inferior labial glands (Estes et al., 1988; Anguimorpha character 16): (0) absent; (1) present.

325. Endolymphatic sacs, extension into the nuchal musculature (Kluge, 67; character C; Etheridge and deQueiroz, 1988; character 35): (0) absent; (1) present, exit through supraoccipital and parietal; (2) present, exit epiotic foramen; (3) present, exit through vagus foramen.

326. Hemipenis, symmetry (Branch, 1982): (0) present; (1) absent.

327. Hemipenis, sulcus (Branch, 1982): (0) simple; (1) divided.

328. Hemipenis, dorsal asulcal ornamentation (Branch, 1982): (0) absence; (1) simple flounces; (2) bifurcated flounces.

329. Hemipenis, m. retractor lateralis posterior substantial situation within hemipenial sheath (Frost and Etheridge, 1989; character 63): (0) absent; (1) present.

330. Hemipenis, horns (Branch, 1982): (0) absent; (1) present, simple; (2) present, multi-cusped.

331. Hemibacula (Gao and Norell, 1998; character 99): (0) absent; (1) present.

332. Neurology, ulnar nerve position (Estes et al., 1988; character 142): (0) "lacertid" style; (1) "varanid" style.

333. Neurology, dorsal leg muscles (Estes et al., 1988; character 143): (0) peroneal nerve present; (1) peroneal nerve absent, interosseus innervation.

334. MAME profundus origin (Gao and Norell, 1998; character 93): (0) supratemporal and parietal; (1) supratemporal only.

335. Meatal closure muscle (Kluge, 1987; character 12): (0) absent; (1) present, L-shaped; (2) present, O-shaped.

336. M. extracolumellaris (Estes et al., 1988; character 135): (0) absent; (1) present.

337. Myology, anterior extension of m. adductor mandibulae posterior (Estes et al., 1988; character 131): (0) at the posterior margin of Meckel's canal; (1) anterior to the posterior 1/4 of the dentary.

338. Myology, m. pseudotemporalis superficialis origin (Estes et al., 1988; character 132): (0) lateral and anterior margins of the supratemporal fossa; (1) also along the medial margin of the temporal fenestra.

339. M. levator pterygoidii, insertion (Gao and Norell, 1998; character 96): (0) extends posteriorly beyond the columellar fossa; (1) restricted anterior to the columellar fossa.

340. M. pseudotemporalis profundus, anterior head (Estes et al., 1988; character 133): (0) absent; (1) present, not expanded; (2) present, expanded.

341. M. pseudotemporalis superficialis, origin (Estes et al., 1988; character 132): (0) limited to the anterior 1/2 of the supratemporal fenestra; (1) extends far posteriorly, onto the posterior 1/3 of the supratemporal fenestra.

342. Bodenaponeurosis, base contact with mandibular fossa (Gao and Norell, 1998; character 98): (0) present; (1) absent, attached only to the caudomesial edge of the coronoid Coding from Gao and Norell, 1998 after Lakjer (1926), Haas (1973), and Rieppel (1980).

343. M. constrictor colli coverage of first ceratobranchials (Gao and Norell, 1998; character 92): (0) absent; (1) present.

344. M. genioglossus lateralis, morphology (Gao and Norell, 1998; character 95): (0) not separate bundles, not inserting on the hyobranchials; (1) separate bundles, some inserting on the hyobranchials.

345. M. rectus abdominis lateralis (Estes et al., 1988; character 134): (0) absent; (1) present.

346. M. episterno-cleido-mastoideus insertion (Gao and Norell, 1998; character 91): (0) mainly on the paroccipital process; (1) extensively on parietal.

347. Urinary bladder (Presch, 1988; character 21): (0) present, complete; (1) present, vestigial; (2) absent.

348. M. levator anguli oris (Abdala and Moro, 2003; character 3): (0) present; (1) absent.

349. M. levator anguli oris, aponeurosis (Abdala and Moro, 2003; character 10): (0) present; (1) absent.

350. Adductor mandibulae externus, tendinous system (Abdala and Moro, 2003; character 12): (0) absent; (1) present.

351. M. adductor mandibulae posterior (Abdala and Moro, 2003; character 32): (0) absent; (1) present.

352. M. pseudotemporalis superficialis (Abdala and Moro, 2003; character 36): (0) absent; (1) present.

353. M. protractor pterygoidei (Abdala and Moro, 2003; character 51): (0) absent; (1) present.

354. M. retractor pterygoidei (Abdala and Moro, 2003; character 53): (0) present; (1) absent.

355. M. intermandibularis anterior superficialis (Abdala and Moro, 2003; character 54): (0) absent; (1) present.

356. M. intermandibularis anterior profundus aponeurosis (Abdala and Moro, 2003; character 55): (0) absent; (1) present.

357. M. depressor mandibulae profundus (Abdala and Moro, 2003; character 65): (0) absent; (1) present.

358. M. mandibulohyoideus II (Abdala and Moro, 2003; character 76): (0) absent; (1) present.

359. M. mandibulohyoideus III (Abdala and Moro, 2003; character 81): (0) absent; (1) present.

360. M. branchiohyoideus aponeurosis (Abdala and Moro, 2003; character 83): (0) absent; (1) present.

361. M. ceratohyoideus (Abdala and Moro, 2003; character 84): (0) absent; (1) present.

362. Muscle "X" (Abdala and Moro, 2003; character 86): (0) absent; (1) present.

363. M. sternohyoideus (Abdala and Moro, 2003; character 92): (0) absent; (1) present.

364. Biogeography: (0) global; (1) Madagascar; (2) South America; (3) North America/Central America; (4) Europe/western Asia; (5) sub-Saharan Africa; (6) northern Africa/Arabia; (7) India; (8) East Asia; (9) Australia.

365. Otooccipitals, contact at the midline (McDowell and Bogert, 1956): (0) absent; (1) present, excludes the supraoccipital from the foramen magum.

366. Splenial, dorsal conrtribution to Meckel's canal (Conrad, 2008): (0) absent; (1) present.

367. Braincase, crista circumfenestralis (Rieppel et al., 2002; character 34): (0) absent; (1) present; (2) present with posterior closure.

368. Angular, forked posterior margin (Norell et al., 2008.): (0) absent; (1) present.

369. Scapula, anterodorsal epicoracoid flange (not a bar) (Conrad, 2008): (0) absent; (1) present.

370. Lacrimal foramen, margins (Rieppel and Grande, 2006): (0) shared between the lacrimal and prefrontal; (1) within the lacrimal; (2) between the prefrontal and maxilla; (3) within the prefrontal; (4) between the prefronal and palatine; (5) between the jugal and prefrontal.

371. Pterygoid, quadrate process (Norell et al., 2006): (0) columnar; (1) medially concave; (2) mediolaterally compressed.

372. External nostril location (Norell et al., 2006): (0) distally placed, near the anterior 1/3 of the snout; (1) middle of snout (middle 1/3); (2) proximally, within the first 1/3 part of snout.

373. Skull, secondary dorsolateral margin to the orbit (modified from Lang, 1989, character 16): (0) absent; (1) present, incomplete; (2) present, complete; (3) present, complete, and mediolaterally broad so that there is no supraorbital fenestra.

374. Zygomatic arch alignment (Sues et al., 1994; character 15): (0) in line with the maxillary tooth row; (1) bowed laterally.

375. Bony external naris, shape of the posterior end (Conrad et al., 2011; character 375): (0) attenuated; (1) broad and rounded.

376. Nasals, breadth relative to the eternal nares (Evans and Barbadillo, 1998; character 130): (0) equal to or greater than the width of the external nares; (1) narrower than the external nares.

377. Nasals, posterior margins (Lang, 1989; character 4): (0) smoothly curved, with the posterior apex located mesially; (1) pointed, with a an attenuated posterior apex; (2) squared posteriorly.

378. Frontal, anterolateral margins exposed as the posteromedial part of the osseous external naris (see discussions of the external nares in mosasauroids and varanids by [11] and [7]): (0) absent; (1) present.

379. Frontal, shape of the anterior ends of the anterolateral processes (Conrad et al., 2011: character 379): (0) anteriorly tapering; (1) parallel sided; (2) anteriorly expanded.

380. Frontal, relative dimensions in length and width (de Queioz, 1987; character 8): (0) frontal longer than wide; (1) frontal wider than long.

381. Frontal, length of the anterolateral extensions of the W-shaped nasofrontal suture (Conrad et al., 2011): (0) short, not extending as far anteriorly as does the midline portion; (1) moderate, extending anteriorly to about the same level as the midline projection of the frontal; (2) elongate, extending anteriorly beyond the level of the midline extension. Note: This character is inapplicable for taxa lacking a W-shaped nasofrontal suture.

382. Parietal, intertemporal width (Sues et al., 1994; character 7): (0) greater than the interorbital width; (1) less than the interorbital width.

383. Maxilla, fossa on the nareal floor anterior to the level of the septomaxilla (Conrad et al., 2011; character 383): (0) absent; (1) present.

384. Maxilla, posterior margin of the anterior nareal fossa (where such a fossa is present) (Conrad et al., 2011; character 384): (0) gently sloping; (1) distinct.

385. Prefrontal, anterolateral overlap of the frontal (Conrad et al., 2011; character 385): (0) does not constrict the exposed frontal margin; (1) with strong dorsomedial flange partly constricting the exposed frontal margin.

386. Prefrontal, posterior extent of the orbital process (Conrad et al., 2011; character 386): (0) terminates within the anterior 1/3 of the orbit; (1) extends for more than 1/3 of the orbit; (2) extends for more than 2/3 of the dorsal orbital margin (approaching the postorbital prefrontal bones). Note that this character was treated as ordered.

387. Postorbital/postorbitofrontal, postorbital extension of the supraorbital ridge (DeBraga and Carooll, 1993; character 14): (0) absent; (1) present.

388. Postfrontal, dorsally overlapping the anterolateral process of the parietal (Conrad et al., 2011; character 388): (0) absent; (1) present as a dorsomedial flange.

389. Jugal, extent of the contact with the squamosal (where a contact is present) (Conrad et al., 2011; character 389): (0) contact narrow, squamosal attenuated near the jugal contact; (1) contact broad, approximately equal to the maximum dorsoventral breadth of the squamosal; (2) extensive contact with the squamosal, such that the jugal anteroventrally underlies the jugal. We treated this as an ordered character.

390 Squamosal, ventral process (de Queiroz, 1987; character 18): (0) present and large; (1) present, without being distinctly offset from the main body of the squamosal; (2) absent.

391. Palpebral, anterior process (Conrad et al., 2011; character 391): (0) absent; (1) present.

392. Palpebral, posterior extent (Conrad et al., 2011; character 392): (0) extends for less than ½ the length of the orbit; (1) extends for ½ the length of the orbit, or more.

393. Palatine, shape (Sues et al., 1994; character 29): (0) tapered posteriorly; (1) squared posteriorly; (2) transversely interdigitating suture.

394. Pterygoid teeth, size of the anterior pterygoid teeth (Bell, 1997; character 43): (0) small, significantly smaller than the marginal teeth; (1) large, similar in size to the marginal teeth.

395. Pterygoid tooth row elevation (Bell, 1997; character 42): (0) absence of a distinctly raised pterygoid tooth ridge; (1) presence of a pronounced, vertical ridge.

396. Prootic, accessory anterostapedial process on the crista prootica (Conrad et al., 2011; character 396): (0) absent; (1) present.

397. Prootic, accessory anterostapedial process shape (Conrad et al., 2011; character 397): (0) posteriorly directed and hook-like; (1) anteroposteriorly elongate tab that is laterally directed; (2) present as a triangular process located dorsolateral to the stapes.

398. Supraoccipital, midline dorsal ridge/keel (Conrad et al., 2011; character 398): (0) absent; (1) present.

399. Otooccipital, posterior development of the crista interfenestralis (Conrad et al., 2011; character 399): (0) crista interfenestralis extends mostly laterally, not hiding the occipital recess in lateral view; (1) crista interfenestralis expanded posterolaterally, partly hiding the occipital recess in lateral view.

400. Quadrate, groove on the external margin of the tympanic crest (DeBraga and Carroll, 1993; character 46): (0) absent; (1) present.

401. Quadrate, position of the infrastapedial arch (if an infrastapedial arch is present) (DeBraga and Carroll, 1993; character 44): (0) ventrally, near the mandibular condyle; (1) dorsally, approximately halfway up long axis of the quadrate.

402. Coronoid, anteromedial prearticular overlap by an anteroventral process of the coronoid (DeBraga and Carroll, 1993; character 60): (0) absent; (1) present; (2) present and conacting the angular. Note that this character is treated as ordered.

403. Angular, dorsolateral extent (de Queiroz, 1987; character 37): (0) angular extends dorsally for more than 1/3 of the lateral surface of the mandible; (1) angular does not extend far up the lateral surface of the mandible and the anterior suture is not visible in lateral view.

404. Dentition, number of tooth crowns (if multiple tooth crowns are present) (de Queiroz, 1987; character 46): (0) bicuspid; (1) tricuspid; (2) four cusps; (3) five or more distinct cusps; (4)serrate tooth crowns with multiple tiny cusps/points.

405. Dentition, marginal teeth increase in size posteriorly (Conrad et al., 2011; character 405): (0) absent; (1) present.

406. Dentition, number of teeth in each premaxilla (number of bilateral premaxillary tooth positions; not including any midline tooth position, if one is present) (modified from Greer, 1970): (0) one; (1) two; (2) three; (3) four; (4) five; (5) six; (6) seven; (7) eight; (8) nine; (9) 10.

407. Dentition, midline premaxillary tooth (Conrad et al., 2011; character 407): (0) absent; (1) present.

408. Dentition, premaxilla-maxilla diastema (Conrad et al., 2011; character 408): (0) absent; (1) present.

409. Coracoid, size of the posterior (secondary) coracoid fenestra/notch (Conrad et al., 2011; character 409): (0) small, similar in size to the glenoid fossa and significantly smaller than the anterior (primary) coracoid fenestra; (1) enlarged, subequal in size to the anterior (primary) coracoid fenestra.

410: Humerus, hook-like and attenuated postglenoid process (Bell, 1997[12]; character 122): (0) absent; (1) present.

411. Ilium, orientation of the dorsal process/iliac blade (DeBraga and Carroll, 1993; character 122): (0) posterodorsally; (1) vertical; (2) anterodorsally.

412. Cross-sectional body shape at midpoint between the skull and vent (Kearney, 2003; character 2): (0) cylindrical; (1) laterally compressed; (2) dorsoventrally depressed; (3) U-shaped.

413. Vertebrae, number of presacral vertebrae (I) (modified from Estes et al., 1988; characters 105 and 106, and from Conrad 2008, character 236): (0) 18; (1) 19; (2) 20; (3) 21; (4) 22; (5) 23; (6) 24; (7) 25; (8) 26; (9) 27. Note that this character and the next (character 414) replace character 236 in this analysis.

414. Vertebrae, number of presacral vertebrae (II) (modified from Estes et al., 1988; characters 105 and 106, and from Conrad, 2008, character 236): (0) 28; (1) 29; (2) 30; (3) 31; (4) 32; (5) 33; (6) 34; (7) 35; (8) 36; (9) 37.

415. Cervical vertebrae, number (modified from Estes et al., 1988; characters 107 and 108, and from Conrad, 2008; character 236): (0) 2; (1) 3; (2) 4; (3) 5; (4) 6; (5) 7; (6) 8; (7) 9; (8) 10; (9) 12. Note that this character replaces character 242 in this analysis.

416. Coloration, caudal bands (Hollingsworth, 1998; character 60): (0) present; (1) absent.

417. Coloration, relative lengths of the caudal color bands ([13]; character 61): (0) subequal for their entire length; (1) increase in length distally such that the more posterior bands are more than 1.5 times the length of the proximal ones.

418. Telencephalon, nucleus sphericus (modified from Northcutt, 1978): (0) absent; (1) present as a poorly/incompletely consolidated group of cells; (2) present as a distinct, cup-shaped body of cells occupying the entire ventricular ridge.

419. Telencephalon, giant cells of the dorsal ventricular ridge (modified from Northcutt, 1978): (0) absent; (1) present.

420: Thalamus, enlargement of the dorsal thalamic nuclei such that the medial dorsal thalamus is displaced (modified from Northcutt, 1978): (0) absent; (1) enlarged nucleus dorsolateralis; (2) enlarged nucleus rotundus, dorsal and ventral thalamic nuclei, and the auditory relay nucleus.

421. Pretectum, pars extensa with distinct and separate subdivisions (modified from Northcutt, 1978): (0) absent; (1) present, divided into dorsal and ventral pretectal nuclei, and the ventral pretectal nucleus.

422. Optic tectum, tectal lamination with subdivision of lamina 7 and hyptertrophy of lamina 14 (modified from Northcutt, 1978): (0) absent, lamina 12 is the main optic fiber layer (the so-called ‘lacertid tectal pattern’); (1) present, lamina 14 is the main optic fiber (the so-called ‘iguanid tectal pattern’).

Additional/new characters added for this analysis include data taken from the studies described above and several additional publications [14-16].

423. Prootic, facial notch in the crista prootica (new): (0) broad and flat, poorly defined because of the absence or weak development of the accessory prootic process; (1) narrow with a rounded apex at the anterolateral border of the accessory prootic process; (2) distinct and distally squared accessory projection.

424. Otooccipital, ventrolateral margin of the exoccipital part of the paroccipital process with accessory lip (new): (0) absent; (1) present.

425. Otooccipital, ‘lip’ ventrolateral margin of the exoccipital part of the paroccipital process (new) (see character 424; scored as “-” for those taxa lacking a ventrolateral paroccipital ‘lip’): (0) expressed as gentle and small ventrolateral expansions at the base of paroccipital process; (1) distinctly offset with a dorsolateral step; (2) present as a distinct ventorolateral process with an independent apex.

426. Otooccipital, distal tip of the paroccipital process (new): (0) dorsal and ventral ends extend to about the same distal level; (1) dorsal tip terminates more proximally than the ventral tip; (2) ventral tip terminates more proximally than the dorsal tip.

427. Integument, convex lateral fold, not associated with dorsal and ventral armor (new): (0) absent; (1) present.

428. Integument, convex lateral body fold extends posterior to the axilla (new): (0) absent; (1) present.

429. Coloration, pale superciliary stripe (new): (0) absent; (1) present.

430. Coloration, dark postocular stripe (new): (0) absent; (1) present.

431. Coloration, pale postorbital stripe (new) : (0) absent; (1) present.

432. Coloration, dorsal color pattern (new): (0) solid; (1) primarily dark with pale speckles; (2) primarily dark, with numerous dark, overlapping sets of pale circles; (3) primarily dark with distinct bands of pale circles; (4) primarily dark with pale transverse stripes; (5) primarily pale with dark spots; (6) pale with dark transverse stripes and speckles; (7) primarily pale with dark circle outlines; (8) primarily pale with dark transverse stripes.

433. Coloration, transverse bands of pale spots (new): (0) solid; (1) with dark centers.

434. Integument, post-aural fold (new): (0) absent; (1) present.

435. Coloration, transverse rows of spots (new): (0) spots separated; (1) lateral rim of spot touches next spot.

436. Coloration, anteroposterior breadth of the transverse color bands (new): (0) similar in size; (1) alternating between broader and narrower bands.

437. Coloration, stripes on neck (new): (0) absent; (1) present.

438. Hemipenis, symmetry (modified from Branch, 1982; Bohme, 1988; Ziegler and Bohme, 1997): (0) present, hemipenial lobes symmetrical; (1) asymmetrical, anterior lobe more robust; (2) asymmetrical, posterior lobe more robust. NOTE: This character replaces character 326.

439. Hemipenis, asulcal hood (modified from Branch, 1982; Bohme, 1988; Ziegler and Bohme, 1997): (0) absent; (1) present, hiding the apex in asulcal view.

440. Hemipenis, asulcal support(s) (Stutsaume) (modified from Bohme, 1988): (0) absent; (1) present.

441. Hemipenis, number of asulcal support(s) (modified from Bohme, 1988): (0) single, midline; (1) divided, paramedian.

442. Hemipenis, retractor muscle (modified from Ziegler and Bohme, 1997): (0) undivided; (1) distally divided.

443. Hemipenis sulcus, location (modified from Branch, 1982; Bohme, 1988; Ziegler and Bohme, 1997): (0) terminates medially; (1) extends onto anterior lobe; (2) extends onto posterior lobe.

444. Hemipenial folds, reach midline (taken from data in Bohme, 1988; Ziegler and Bohme, 1997): (0) absent; (1) present.

445. Paryphasmata (Ziegler and Bohme, 1997): (0) absent; (1) present.

446. Paryphasmata, size (modified from Ziegler and Bohme, 1997): (0) small and numerous; (1) relatively large, equal to more than 12% of the hemipenial breadth.

447. Paryphasmata, bands reach midline (modified from Bohme, 1988; Ziegler and Bohme, 1997): (0) absent; (1) present.

448. Paryphasmata. number of rows reaching midline (modified from Branch, 1982; Bohme, 1988; Ziegler and Bohme, 1997): (0) 1-4; (1) 3-6; (2) 5-8; (3) 7-10; (4) 9-12; (5) 11-14; (6) 13-16; (7) 15-18; (8) 17-20; (9) 19-22.

449. Paryphasmata, lobe-shaped paryphasman patch on pedicel (modified from Ziegler and Bohme, 1997): (0) absent; (1) present.

450. Hemipenis pedicel, transverse folds (modified from Bohme, 1988): (0) present on pedicel; (1) absent from pedicel.

451. Hemipenis trunk, asulcal pit (modified from Branch, 1982; Bohme, 1988; Ziegler and Bohme, 1997): (0) absent; (1) present, midline; (2) present, paired and paramesial.

452. Hemipenis, trunk ornamentation (modified from Branch, 1982; Bohme, 1988; Ziegler and Bohme, 1997): (0) transverse ridges, defining folds; (1) criss-crossing ridges forming a diamond pattern of sulci; (2) multiple, conical apices; (3) grid pattern of ridges, describing rectangular sulci.

453. Hemipenis trunk, number of transverse folds reaching midline (modified from Branch, 1982; Bohme, 1988; Ziegler and Bohme, 1997): (0) 1-4; (1) 3-6; (2) 5-8; (3) 7-10; (4) 9-12; (5) 11-14; (6) 13-16; (7) 15-18; (8) 17-20; (9) 19-22.

454. Hemipenis trunk, bilateral sulcal swellings near the base of the trunk (modified from Bohme, 1988): (0) absent; (1) present.

455. Hemipenis apex, distal subdivision (modified from Branch, 1982; Bohme, 1988; Ziegler and Bohme, 1997): (0) absent, apex unicapitate; (1) present, hemipenis bilobed.

456. Hemipenis apex, ornamentation (modified from Bohme, 1988): (0) naked; (1) with folds; (2) with folds and paryphasmata.

457. Hemipenis apex, distal extent of sperm sulcus (taken from data in Bohme, 1988): (0) ends proximal of the tip; (1) extends onto the tip.

458. Hemipenis apex, shape (from data in Branch, 1982; Bohme, 1988; and Ziegler and Bohme, 1997): (0) rounded/squared; (1) with attenuated/triangular distal projections.

459. Hemibaculum, symmetry (modified from Branch, 1982; Bohme, 1988; Ziegler and Bohme, 1997): (0) symmetrical; (1) asymmetrical, anterior horn more robust; (2) asymmetrical, posterior horn more robust. Note that this differs from the hemipenial symmetry and does not invariably co-vary with the codings of hemipenial symmetry.

460. Hemibaculum, neck (modified from Branch, 1982; Bohme, 1988; Ziegler and Bohme, 1997): (0) absent; (1) present.

461. Hemibaculum, distal concavity on the sulcal surface (modified from Branch, 1982; Bohme, 1988; Ziegler and Bohme, 1997): (0) absent; (1) present on the anterior hook; (2) present on the posterior hook.

462. Hemibacula, anterior horn morphology (modified from Ziegler and Bohme, 1997): (0) simple, attenuated; (1) broad, flattened; (2) with multiple attenuated apices; (3) with 3-4 large hooks; (4) bifurcate.

463. Hemibacula, posterior horn (modified from Ziegler and Bohme, 1997): (0) simple, attenuated; (1) broad, flattened; (2) with multiple attenuated apices; (3) with 3-4 large hooks; (4) bifurcate.

464. Hemiclitoris, symmetry (modified from Branch, 1982; Bohme, 1988; Ziegler and Bohme, 1997): (0) present, hemiclitoral lobes symmetrical; (1) anterior lobe more robust; (2) posterior lobe more robust.

465. Hemiclitoris, asulcal hood (modified from Branch, 1982; Bohme, 1988; Ziegler and Bohme, 1997: (0) absent; (1) present, hiding apex in asulcal view.

466. Hemiclitoral folds, reach midline (taken from data in Bohme, 1988; Ziegler and Bohme, 1997): (0) absent; (1) present.

467. Hemiclitoris trunk, number of transverse folds reaching midline (modified from Branch, 1982; Bohme, 1988; Ziegler and Bohme, 1997): (0) 1-4; (1) 3-6; (2) 5-8; (3) 7-10; (4) 9-12; (5) 11-14; (6) 13-16.

468. Paryphasmata on hemiclitoris, bands reach midline (modified from Bohme, 1988; Ziegler and Bohme, 1997): (0) absent; (1) present.

469. Paryphasmata on hemiclitoris, number of rows reaching midline (modified from Branch, 1982; Bohme, 1988; Ziegler and Bohme, 1997): (0) 1-4; (1) 3-6; (2) 5-8; (3) 7-10; (4) 9-12; (5) 11-14; (6) 13-16.

470. Hemiclitoris trunk, asulcal pit (modified from Branch, 1982; Bohme, 1988; Ziegler and Bohme, 1997): (0) absent; (1) present.

471. Hemibaubella, visible on apex when hemiclitoris extended (from data in Ziegler and Bohme, 1997): (0) present; (1) absent.

472. Hemibaubellum, symmetry (modified from Branch, 1982; Bohme, 1988; Ziegler and Bohme, 1997): (0) symmetrical; (1) asymmetrical, anterior horn more robust; (2) asymmetrical, posterior horn more robust.

473. Hemibaubellum, neck (modified from Branch, 1982; Bohme, 1988; Ziegler and Bohme, 1997): (0) absent; (1) present.

474. Hemibaubellum, distal concavity on the sulcal surface (modified from Branch, 1982; Bohme, 1988; Ziegler and Bohme, 1997): (0) absent; (1) present on anterior horn; (2) present on posterior horn.

475. Hemibaubellum, anterior horn morphology (modified from Ziegler and Bohme, 1997): (0) simple, attenuated; (1) broad, flattened; (2) with multiple, attenuated, apices; (3) squared; (4) mitten shaped; (5) broad, rounded.

476. Hemibaubellum, posterior horn (modified from Ziegler adn Bohme, 1997): (0) simple, attenuated; (1) broad, flattened; (2) with multiple, attenuated, apices; (3) squared; (4) mitten shaped; (5) broad, rounded.

477. Hemiclitoral pedicel, hook-like projections (modified form data in Ziegler and Bohme, 1997): (0) absent; (1) present.

478. Foretongue color (from Sprackland, 1999): (0) pink; (1) yellow; (2) purple or blue/black.

479. Hemipenis, anterior flattened baculum (new): (0) simple; (1) with accessory spur; (2) denticulate.

480. Hemipenis, posterior flattened baculum (new): (0) simple; (1) with accessory spur; (2) denticulate.

481. Hemiclitoris, anterior flattened hemibaubellum (new): (0) simple; (1) with accessory spur; (2) denticulate.

482. Hemiclitoris, posterior flattened hemibaubellum (new): (0) simple; (1) with accessory spur; (2) denticulate.

483. Hemipenis, median folds not reaching midline (new): (0) 1-4; (1) 3-6; (2) 5-8; (3) 7-10; (4) 9-12; (5) 11-14; (6) 13-16; (7) 15-18; (8) 17-20.

484. Paryphasmata, symmetry (new): (0) symmetrical; (1) anterior lobe with more paryphasmata folds; (2) posterior lobe with more paryphasmata folds.

485. Fleshy nostril, shape (Welton et al., 2010): (0) subovate; (1) slit-like.

486. Coloration, snout color (new): (0) similar to the supraorbital region; (1) dark; (2) pale.

487. Coloration, pale supralabial stripe (new): (0) absent; (1) present.

488. Snout, bulbous and fleshy anterodorsal expansions (new): (0) absent; (1) present.

489. Squamation, enlargement of nuchal scales (new): (0) absent, nuchal scales similar in size and morphology to surrounding scales of the dorsum (enlarged tubercles may still be present); (1) present, scales of the nuchal region more-or-less uniformly enlarged as compared to those of the anterior torso.

490. Squamation, enlarged nuchal scales (new***): (0) flat, similar in thickness to other scales of the dorsum; (1) rounded/domed; (2) keeled.

491. Coloration, longitudinal caudal stripes (new): (0) absent; (1) present.

492. Coloration, orientation of nuchal stripes (new): (0) absent; (1) transverse; (2) longitudinal.

493. Coloration, tail tip (new): (0) brown (differing from more proximal portions of tail); (1) notably paler than more proximal tail (yellow/white/orange); (2) notably darker than more proximal tail (black/blue); (3) pattern from the more proximal part of the tail is retained to posterior end of the tail.

**References**

1. Rieppel O (1980) The sound transmitting apparatus of primitive snakes and its phylogenetic significance. Zoomorphology 96: 45-62.

2. Estes R, de Queiroz K, Gauthier J (1988) Phylogenetic relationships within Squamata. In: Estes R, Pregill G, editors. Phylogenetic relationships of the lizard families. Stanford: Stanford University Press. pp. 119-281.

3. Caldwell MW (1999) Squamate phylogeny and the relationships of snakes and mosasauroids. Zoological Journal of the Linnean Society 125: 115-147.

4. Gao K-Q, Norell MA (1998) Taxonomic revision of *Carusia* (Reptilia: Squamata) from the Late Cretaceous of the Gobi Desert and phylogenetic relationships of anguimorphan lizards. American Museum Novitates 3230: 1-51.

5. Rieppel O, Zaher H (2000) The intramandibular joint in squamates, and the phylogenetic relationships of the fossil snake *Pachyrhachis problematicus* Haas. Fieldiana (Geology), new series 43: 1-69.

6. Frost DR, Etheridge R (1989) A phylogenetic analysis and taxonomy of iguanian lizards (Reptilia: Squamata). University of Kansas Museum of Natural History, Miscellaneous Publications 81: 1-65.

7. Conrad JL (2008) Phylogeny and systematics of Squamata (Reptilia) based on morphology. Bulletin of the American Museum of Natural History 310: 1-182.

8. Rieppel O (1984) The structure of the skull and jaw adductor musculature in the Gekkota, with comments on the phylogenetic relationships of the Xantusiidae (Reptilia: Lacertilia). Zoological Journal of the Linnean Society 82: 291-318.

9. Gauthier JA, Estes R, de Queiroz K (1988) A phylogenetic analysis of Lepidosauromorpha. In: Estes R, Pregill G, editors. Phylogenetic relationships of the lizard families. Stanford: Stanford University Press. pp. 15-98.

10. Wiens JJ, Etheridge RE (2003) Phylogenetic relationships of hoplocercid lizards: coding and combining meristic, morphometric, and polymorphic data using step matrices. Herpetologica 59: 375-398.

11. Caldwell MW, Carroll RL, Kaiser H (1995) The pectoral girdle and forelimb of *Carsosaurus marchesetti* (Aigialosauridae), with a preliminary phylogenetic analysis of mosasauroids and varanoids. Journal of Vertebrate Paleontology 15: 516-531.

12. Bell GL, Jr. (1997) A phylogenetic revision of North American and Adriatic Mosasauroidea. In: Callaway JM, Nicholls EL, editors. Ancient marine reptiles. San Diego: Academic Press. pp. 293-332.

13. Hollingsworth BD (1998) The systematics of chuckwallas (*Sauromalus*) with a phylogenetic analysis of other iguanid lizards. Herpetological Monographs 12: 38-191.

14. Branch WR (1982) Hemipeneal morphology of platynotan lizards. Journal of Herpetology 16: 16-38.

15. Ziegler T, Böhme W (1997) Genitalstrukturen un Paarungsbiologie bei squamaten Reptilien, speciell den Platynota, mit Bemerkungen zur Systematik. Mertensiella 8: 1-210.

16. Böhme W (1988) Zur Genitalmorphologie der Sauria; Funktionelle und stammesgeschichtliche Aspekte. Bonner Zoologische Monographien 27: 1-176.
