# Supplementary material for: Earliest Example of a Giant Monitor Lizard (Varanus, Varanidae, Squamata)
Source: PLoS One. 2012 Aug 10;7(8):e41767. doi: 10.1371/journal.pone.0041767 (PMC3416840; doi:10.1371/journal.pone.0041767)
Supplement: TEXT S3 — FULL CONSENSUS TREES FOR THE ANALYSES. Below we include the strict and the Adams consensus results for the results of the TNT [54], [89], [90] analyses as read out by PAUP* [91]. Note that the strict consensus lacks resolution because of the volatile nature of some taxa (e.g., Jebel Qatrani ‘Varanus’). (DOC) [file pone.0041767.s004.doc]

**Earliest example of a giant monitor lizard (*Varanus*, Varanidae, Squamata)**

Jack L. Conrad^1^

Ana M. Balcarcel^2^

Carl M. Mehling^2^

^1^ Anatomy Department, New York College of Osteopathic Medicine, Old Westbury, NY

^2^ Department of Vertebrate Paleontology, American Museum of Natural History, New York, NY

**SUPPORTING INFORMATION**

**TEXT S4: FULL CONSENSUS TREES FOR THE ANALYSES**

Below we include the strict and the Adams consensus results for the results of the TNT [1-3] analyses as read out by PAUP* [4]. Note that the strict consensus lacks resolution because of the volatile nature of some taxa (e.g., Jebel Qatrani ‘*Varanus*’).

Strict consensus tree:

/--------------------------------------------------------------------------------- Shinisaurus

|

+--------------------------------------------------------------------------------- feisti

|

+--------------------------------------------------------------------------------- NecrosaurusCAY

|

+--------------------------------------------------------------------------------- NecrosaurusEUC

|

+--------------------------------------------------------------------------------- Proplatynotia

|

+--------------------------------------------------------------------------------- Paravaranus

|

+--------------------------------------------------------------------------------- Saniwides

|

| /-------------- Cherminotus

+------------------------------------------------------------------+

| \-------------- Lanthanotus

|

+--------------------------------------------------------------------------------- Ovoo

|

+--------------------------------------------------------------------------------- Aiolosaurus

|

+--------------------------------------------------------------------------------- Telmasaurus

|

| /-------------- Dolichosaurus

| /--------------------------+

| | \-------------- ConiasaurusCRAS

| |

+---------------------------------------+ /-------------- Adriosaurus

| | /------------+

| | | \-------------- PontoLES

| \-------------+

| \--------------------------- Aigialosaurus

|

+--------------------------------------------------------------------------------- SaniwaENS

|

+--------------------------------------------------------------------------------- Vacanthurus

|

+--------------------------------------------------------------------------------- Vbaritji

|

| /-------------- Vbeccarii

| |

| +-------------- Vkeithhornei

| /-----------------------------------------------------+

| | +-------------- Vprasinus

| | |

| | \-------------- Vkordensis

| |

| | /-------------- Vdoreanus

+------------+ /------------+

| | | \-------------- Vyuwonoi

| | /--------------------------+

| | | \--------------------------- Vjobiensis

| | |

| | | /--------------------------- VindicusAUS

| \-------------+ |

| | /-------------+ /-------------- VindicusF

| | | \------------+

| \------------+ \-------------- VindicusSI

| |

| \----------------------------------------- Vmelinus

|

+--------------------------------------------------------------------------------- mytilini

|

+--------------------------------------------------------------------------------- Vdracaena

|

+--------------------------------------------------------------------------------- Vbengalensis

|

+--------------------------------------------------------------------------------- Vbivittatus

|

+--------------------------------------------------------------------------------- Vbrevicauda

|

+--------------------------------------------------------------------------------- Vcumingi

|

+--------------------------------------------------------------------------------- Vdumerilii

|

+--------------------------------------------------------------------------------- Veremius

|

+--------------------------------------------------------------------------------- Vflavescens

|

+--------------------------------------------------------------------------------- Vgiganteus

|

+--------------------------------------------------------------------------------- Vgilleni

|

+--------------------------------------------------------------------------------- Vglebopalma

|

+--------------------------------------------------------------------------------- Vgouldii

|

+--------------------------------------------------------------------------------- cf marathonensis

|

+--------------------------------------------------------------------------------- Vgriseus

|

+--------------------------------------------------------------------------------- Vkingorum

|

+--------------------------------------------------------------------------------- Vkomodoensis

|

+--------------------------------------------------------------------------------- Vmarmoratus

|

+--------------------------------------------------------------------------------- Vmertensi

|

+--------------------------------------------------------------------------------- Vmitchelli

|

+--------------------------------------------------------------------------------- Vnebulosus

|

+--------------------------------------------------------------------------------- Vniloticus

|

+--------------------------------------------------------------------------------- Vrudicollis

|

+--------------------------------------------------------------------------------- Valbigularis

|

+--------------------------------------------------------------------------------- Vexanthematicus

|

+--------------------------------------------------------------------------------- Vornatus

|

+--------------------------------------------------------------------------------- Vrusingensis

|

+--------------------------------------------------------------------------------- Volivaceus

|

+--------------------------------------------------------------------------------- Vhooijeri

|

+--------------------------------------------------------------------------------- Vpanopteshorni

|

+--------------------------------------------------------------------------------- Vpanoptespanopte

|

+--------------------------------------------------------------------------------- Vpilbarensis

|

+--------------------------------------------------------------------------------- Vprimordius

|

+--------------------------------------------------------------------------------- Vprisca

|

+--------------------------------------------------------------------------------- Vrosenbergi

|

+--------------------------------------------------------------------------------- Vsalvadorii

|

+--------------------------------------------------------------------------------- Vsalvator

|

+--------------------------------------------------------------------------------- Vscalaris

|

+--------------------------------------------------------------------------------- Vsemiremex

|

+--------------------------------------------------------------------------------- Vspenceri

|

+--------------------------------------------------------------------------------- Vstorri

|

+--------------------------------------------------------------------------------- Vtimorensis

|

+--------------------------------------------------------------------------------- Vtogianus

|

+--------------------------------------------------------------------------------- Vtristis

|

+--------------------------------------------------------------------------------- Vvarius

|

+--------------------------------------------------------------------------------- Vmarathonensis

|

+--------------------------------------------------------------------------------- Yale Quarry

|

+--------------------------------------------------------------------------------- Birket Qarun

|

\--------------------------------------------------------------------------------- Jebel Qatrani

Adams consensus tree:

/--------------------------------------------------------------------------------- Shinisaurus

|

| /----- feisti

| /----+

| | \----- NecrosaurusCAY

| /---+

| | \---------- NecrosaurusEUC

| |

| /-----------------------------------------------+-------------- Saniwides

| | |

| | \-------------- Telmasaurus

| |

| | /--------------------------------------------------------- SaniwaENS

| | |

| | | /----- Vacanthurus

| | | /----+

| | | | \----- Vbaritji

| | | /---+

| | | | \---------- Vstorri

| | | /----+

| | | | | /----- Vkingorum

| | | | \--------+

| | | /----+ \----- Vprimordius

| | | | |

| | | | \------------------- Vgilleni

| | | /----+

| | | | | /----- Vbrevicauda

| | | | \------------------+

| | | | \----- Veremius

| | | |

| | | | /----- Vglebopalma

| | | | /-------------+

| | | | | \----- Vpilbarensis

| | | | |

| | | /---+ | /----- Vmitchelli

| | | | +---------+ /----+

| | | | | | | \----- Vtimorensis

| | | | | | /---+

| | | | | | | \---------- Vscalaris

| | | | | \----+

| | | | | \-------------- Vtristis

| | | | |

| | | | \----------------------------- Vsemiremex

| | | |

| | | | /----- Vgiganteus

| | | /----+ /-------------+

| | | | | | \----- Vmertensi

| | | | | |

| | | | | | /---------- Vgouldii

| | | | | /----+ |

| | | | | | | /---+ /----- Vpanopteshorni

| | | | | | | | \----+

| | | | | | \----+ \----- Vpanoptespanopte

| | | | | | |

| | | | | | \-------------- Vrosenbergi

| | | | | |

| | | | \--------+ /---------- Vkomodoensis

| | | | | |

| | | | | /---+ /----- Vprisca

| | | | | | \----+

| | | | | /----+ \----- Vvarius

| | | | | | |

| | | | \----+ \-------------- Vsalvadorii

| +----+ | |

| | | | \------------------- Vspenceri

| | | |

| | | | /-------------- mytilini

| | | /----+ |

| | | | | | /----- Vbengalensis

| /----+ | | | /----+ /----+

| | | | | | | | | \----- Vnebulosus

| | | | | | | \---+

| | | | | | /---------+ \---------- Vdumerilii

| | | | | | | |

| | | | | | | \------------------- Vflavescens

| | | | | | |

| | | | | | | /----- Vbivittatus

| | | | | | | /----+

| | | | | | | | \----- Vsalvator

| | | | | +--------+ /---+

| | | | | | | | \---------- Vtogianus

| | | | | | | /----+

| | | | | | | | \-------------- Vmarmoratus

| | | | | | | /----+

| | | | | | | | \------------------- Vcumingi

| | | | | | \----+

| | | | | | \------------------------ Vrudicollis

| | | | /----+ |

| | | | | | \-------------------------------------- Vmarathonensis

| | | | | |

| | | | | | /----- Vbeccarii

| | | | | | /----+

| | | | | | | \----- Vkeithhornei

| | | | | | |

| | | | | | /-------------+---------- Vprasinus

| | | | | | | |

| | | | | | | \---------- Vkordensis

| | | | | | |

| | | | | | | /----- Vdoreanus

| | | | | | | /----+

| | | | | | /----+ | \----- Vyuwonoi

| | | | | | | | /--------+

| | | | /---+ | | | | \---------- Vjobiensis

| | | | | | | | | |

| /---+ | | | | | | | | /---------- VindicusAUS

| | | | | | | | | \----+ |

| | | | | | | | | | /---+ /----- VindicusF

| | | | | | | \-------------+ | | \----+

| | | | | | | | \----+ \----- VindicusSI

| | | | | | | | |

| | | | | | | | \-------------- Vmelinus

| | | | | | | |

| | | | | | | | /----- Volivaceus

| | | | | | | \-----------------------+

| | | | \----+ | \----- Vhooijeri

| | | | | |

| | | | | \------------------------------------------------ Vgriseus

| | | | |

| | | | +---------------------------------------------------- Vdracaena

| | | | |

| | | | | /---------- Vniloticus

| | | | | |

| | | | | /---+ /----- Valbigularis

| | | | | | \----+

| /----+ | | | /----+ \----- Vexanthematicus

| | | | | | | |

| | | | | \--------------------------------+ \-------------- Vornatus

| | | | | |

| | | | | \------------------- Vrusingensis

| | | | |

| | | | \-------------------------------------------------------------- Jebel Qatrani

| | | |

| | | | /----- Cherminotus

| | | +-------------------------------------------------------------+

| | | | \----- Lanthanotus

| | | |

| | | +------------------------------------------------------------------- Ovoo

+----+ | |

| | | \------------------------------------------------------------------- Aiolosaurus

| | |

| | +----------------------------------------------------------------------- Proplatynotia

| | |

| | +----------------------------------------------------------------------- cf marathonensis

| | |

| | \----------------------------------------------------------------------- Yale Quarry

| |

| | /----- Dolichosaurus

| | /--------+

| | | \----- ConiasaurusCRAS

| | |

| \-------------------------------------------------------------+ /----- Adriosaurus

| | /----+

| | | \----- PontoLES

| \---+

| \---------- Aigialosaurus

|

+--------------------------------------------------------------------------------- Paravaranus

|

\--------------------------------------------------------------------------------- Birket Qarun

**References**
